# Targeting EGFR-binding protein SLC7A11 enhancing antitumor immunity of T cells via inducing MHC-I antigen presentation in nasopharyngeal carcinoma

**DOI:** 10.1038/s41419-024-07327-9

**Published:** 2025-01-16

**Authors:** Haihua Wang, Songqing Fan, Yuting Zhan, Yue Xu, Yao Du, Jiadi Luo, Hongjing Zang, Shuping Peng, Weiyuan Wang

**Affiliations:** 1 Department of Pathology, The Second Xiangya Hospital, Central South University, 410011 Changsha, Hunan China; 2 Department of Gastroenterology, The Second Affiliated Hospital of Hainan Medical University, 570216 Haikou, Hainan China; 3 Hunan Clinical Medical Research Center for Cancer Pathogenic Genes Testing and Diagnosis, 410011 Changsha, Hunan China; 4 Cancer Research Institute, School of Basic Medical Science, Central South University, 410078 Changsha, Hunan China; 5 Department of Pathology, The Xiangya Hospital, Central South University, 410008 Changsha, Hunan China

**Keywords:** Oncogenesis, Predictive markers

## Abstract

Approximately 80% of nasopharyngeal carcinoma (NPC) patients exhibit EGFR overexpression. The overexpression of EGFR has been linked to its potential role in modulating major histocompatibility complex class I (MHC-I) molecules. We discovered that EGFR, operating in a kinase-independent manner, played a role in stabilizing the expression of SLC7A11, which subsequently inhibited MHC-I antigen presentation. This mechanism, in turn, provided protection to NPC cells against T cell-mediated cytotoxicity. The underlying molecular processes revealed that the high and stable expression of SLC7A11 hindered the nuclear entry of GR, thereby suppressing TAP1 transcription and the presentation of MHC-I molecules. Additionally, elevated SLC7A11 expression led to an increase in FAF2 expression and triggered ERAD-dependent degradation of MHC-I, resulting in a reduction of MHC-I molecules on the cell membrane. The NPC patients exhibiting high EGFR and low MHC-I expression, combined with a scarcity of CD8^+^ T cells (EGFR^high^MHC-I^low^CD8^few^ phenotype), experienced considerably shorter overall survival times compared to other situations. What is more, our study demonstrated that sorafenib had the capability to enhance the MHC-I antigen presentation process, thereby facilitating T cell-mediated killing of NPC cells via targeting SLC7A11. Consequently, targeting SLC7A11 with sorafenib emerges as a promising therapeutic strategy for the treatment of NPC.

## Introduction

Nasopharyngeal carcinoma (NPC), a malignancy originating from the epithelial cells of the nasopharyngeal mucosa, is a complex disease with its multifaceted etiological and pathogenic factors, encompassing and pathogenesis linked to genetic, racial, and environmental influences [1]. While patients diagnosed with early-stage NPC often respond positively to conventional treatments such as radiotherapy and chemotherapy, the prognosis takes a more ominous turn for approximately 90% of patients diagnosed at advanced stages [2]. Moreover, a substantial 20-30% of patients experience metastasis following standard radiotherapy and chemotherapy, leading to a pronounced reduction in median survival time [3].

Although immune checkpoint inhibitors (ICIs) have shown promise in treating some recurrent or metastatic NPC cases, a significant proportion of patients (60-70%) fail to achieve enduring relief [4]. To mount an effective T cell response, it is imperative to have immunogenic tumor antigens [5]. In the case of NPC, the loss of this antigen is more pronounced at metastatic sites as compared to primary tumors [6]. Antigen peptides must associate with MHC class I molecules to form complexes that can activate immune cells when presented on the cell membrane. Diminished or absent MHC-I expression restricts the presentation of tumor antigen, impeding the activation and recruitment of CD8^+^T lymphocyte. This can lead to immune evasion by the tumor and render immunotherapy less effective [7]. Patients with NPC who exhibit aberrations in MHC-I expression tend to have poorer survival outcomes [8]. Thus, unraveling the intricate mechanisms of MHC-I antigen presentation in NPC cells is of paramount importance for the development of effective treatment strategies.

Epidermal growth factor receptor type I receptor (EGFR or HER 1), a member of the ErbB/HER family with intracellular tyrosine kinase activity, is recognized as an oncogene [9]. EGFR plays a multifaceted role in NPC, where it not only enhances Epstein-Barr virus (EBV) infection but also exerts its influence on cell proliferation, cycle progression, angiogenesis, invasion, and metastasis [10]. Additionally, the EGFR pathway is known to be involved in the downregulation of MHC-I expression in human monocytes [11]. Notably, the anti-EGFR antibody, Nimotuzumab, has been found to increase the expression of HLA Class I molecules on tumor cells [12]. In patients with head-neck squamous carcinoma, STAT1-induced upregulation of HLA class I enhances immunogenicity and improves the clinical response to anti-EGFR mAb Cetuximab therapy [13]. Furthermore, in non-small cell lung cancer, mutational activation of EGFR has been linked to the downregulation of MHC class I expression through extracellular signal-regulated kinase pathway [14]. Despite these findings, the research on the role of EGFR in the MHC-I antigen presentation process in NPC remains limited. Consequently, investigating the interaction mechanism between EGFR, MHC-I, and T cell holds significant promise for advancing our understanding of NPC.

It is worth noting that the cytoplasmic domain of EGFR (Δ N-term) interacts with the central region of SLC7A11 (amino acids 45-470), resulting in the stabilization of its expression and an augmentation in cysteine uptake and GSH synthesis [15]. SLC7A11, a component of the Xc^-^ system, serves as a plasma membrane amino acid transporter, facilitating the exchange of extracellular cystine with intracellular glutamate [16]. The expression of SLC7A11 has been found to have a negative correlation with the infiltration of CD8^+^T cells in several types of cancers, including DLBC, ESCA, HNSC, LUAD, LUSC, TGCT, and THCA [17]. This transporter plays a crucial role in modulating intracellular metabolism and contributes to the tumor immune response [18]. Notably, cancer immunotherapy has shown the most promise in patients exhibiting low SLC7A11 expression [17]. Inhibition of antiporter cystine uptake disrupts MHC class I cross-presentation [19]. Therefore, comprehending the interplay between EGFR and SLC7A11 in the context of NPC, and their impact on the MHC-I antigen presentation, is vital for the development of SLC7A11-targeted therapies aimed at enhancing the immune response of CD8^+^T cells.

In this study, we have made several significant findings. We discovered that EGFR, operating in a kinase-independent manner, plays a pivotal role in stabilizing the expression of SLC7A11. This stabilization process has far-reaching consequences, including the limitation of GR nuclear entry, which in turn inhibits TAP1 transcription and the subsequent presentation of MHC-I molecules. Simultaneously, elevated and stable SLC7A11 expression enhances the expression of FAF2 and triggers ERAD-dependent degradation of MHC-I, ultimately resulting in reduced MHC-I on the cell membrane. Additionally, our investigation delved into the therapeutic potential of sorafenib, particularly in the context of targeting SLC7A11 in NPC. We identify that sorafenib has the ability to enhance the MHC-I antigen presentation process. This enhancement, in turn, facilitates T cell-mediated killing of NPC cells both in vitro experiments and in vivo settings. Collectively, our findings emphasize the critical importance of augmenting MHC-I antigen presentation as a promising avenue in the treatment of NPC. Furthermore, our results highlight the potential of targeting SLC7A11 as a therapeutic strategy with the capacity to enhance the immune response in the context of NPC treatment.

## Materials and methods

### Ethical statement

The protocols for the immunohistochemistry experiment were approved by the Xiangya Hospital of Central South University Ethics Review Board (Scientific and Research Ethics Committee, No. 2023030266). All research procedures were conducted in strict compliance with relevant guidelines/regulations. Written informed consent were obtained from all research participants. In the case of juvenile, written consent was signed by their caretakers or legal guardians on their behalf.

NCG (NOD/ShiLtJGpt-Prkdcem26Cd52Il2rgem26Cd22/Gpt) and nude male mices (20 g/per) were purchased from gempharmatech and were provided with a quality certificate for experimental animals. These mice were housed in the SPF level barrier system of Hunan Yuantai Biotechnology Company. The animal experiment program was approved by the Xiangya Hospital of Central South University Ethics Review Board (Scientific and Research Ethics Committee, No. 2023030266), ensuring full adherence to ethical regulations.

### Patient cohorts

We collected 443 patients with NPC who were diagnosed at the Xiangya Hospital, Central South University, between 2011 and 2017. Expert pathologists assessed all tumor samples using the WHO Nasopharyngeal carcinoma histological classification. The staging classification for the present analysis was determined in accordance with the criteria outlined in the 8^th^ edition of the AJCC/UICC TNM staging system for NPC. Inclusion and exclusion criteria were described previously [20].

### Immunohistochemistry and scores

The immunohistochemistry experiment was conducted following the protocol our former study [21]. The primary antibody against EGFR was diluted in a 1:1000 ratio (Rabbit polyclonal antibody, Catalogue 26864-1-AP; Proteintech Group). The dilution of other primary antibodies was as follows: MHC-I, 1:8500 (Rabbit polyclonal antibody, Catalogue 15240-1-AP; Proteintech Group); GR, 1:8000 (Mouse Monoclonal antibody, Catalogue 66904-1-Ig; Proteintech Group); TAP1, 1:8000 (Rabbit polyclonal antibody, Catalogue 11114-1-AP; Proteintech Group); FAF2, 1:5000 (Rabbit polyclonal antibody, Catalogue 16251-1-AP; Proteintech Group); CD8, 1:5000 (Rabbit Monoclonal antibody, Catalogue MAB-0514, MXB Biotechnologies). Proteins expression was evaluated independently by FSQ and WWY, who were blinded to the clinicopathological data. The immunohistochemical staining was assessed and scored as described previously [21]. Briefly, the staining intensity for each antibody was scored as 0 (negative), 1 (weak), 2 (moderate), and 3 (strong). The percentage of stained cells was scored as 0 (no staining), 1 (1–25%), 2 (26–50%), 3 (51–75%), and 4 (76–100%). All of them were both based on the observation of tumor cells. The expression scores were calculated by multiplying the intensity score by the extent of tumor staining (0, 1, 2, 3, 4, 6, 8, 9, and 12). Staining scores <9 and ≥9 indicated low and high expression, respectively, for an optimal cut‐off level for EGFR protein. The cut‐off level of other primary antibodies was as follows: MHC-I protein, staining scores <4 and ≥4 indicated low and high expression, respectively; GR protein, staining scores ≤3 and >3 indicated low and high expression, respectively; TAP1 protein, staining scores ≤4 and >4 indicated low and high expression, respectively; FAF2 protein, staining scores ≤4 and >4 indicated low and high expression, respectively. For CD8^+^T cells infiltration, we focused on the areas of cancers with immune infiltrates. Few was < 10%/HPF (hot pots). More was ≥10%/HPF (hot pots).

### Microarray data

We downloaded GSE53819 from TCGA database (https://tcga-data.nci.nih.gov/tcga/). Based on the Affymetrix GPL6480 platform, GSE53819 consisted of 18 NPC samples and 18 normal nasopharyngeal tissues [22].

### Cell lines and cell culture

The immortalized nasopharyngeal epithelial cell line NP69 was maintained in keratinocyte serum-free medium (Invitrogen) supplemented with 5% heat-inactivated fetal calf serum, 25 μg/mL bovine pituitary extract, and 0.2 ng/mL recombinant epidermal growth factor according to the previous study [23]. Human NPC cell lines (5-8F, 6-10B, and SUNE1) were cultured in DMEM (Biological Industries, Israel) supplemented with 10% fetal bovine serum (Gibco, USA). The culture dishes were placed in the incubator at 37 °C, 5% CO_2_. All cell lines were recently authenticated using short tandem repeat (STR) profiling using the Microread Gene Technology (Beijing, China).

### Small interfering RNAs and transfection

Small interfering RNAs (siRNAs) and a control siRNA (siNC) were obtained from

RiboBio Technology, Guangzhou, China. The following siRNAs were used: siEGFR-1: GAGGAAATATGTACTACGA; siEGFR-2: GGAGCGAATTCCTTTGGAA. siSLC7A11-1: GGAAGAGATTCAAGTATTA; siSLC7A11-2: CTTGCAATATGTATATCCA. siGR-2: TAGAAGTCCATCACATCTC; siGR-3: TTTCTCTTGCTTAATTACC. siTAP1-1: CCGATACCTTCACTCGAAA; siTAP1-2: GTGACGGGATCTATAACAA. siFAF2-2: GGCATGCTCTACAAACAAA; siFAF2-3: GTGTCAGAACGCCTAGAAA. The sequence of siNC was confidential and not disclosed by company. Transfection was carried out with 50 nM siRNAs per sample using polyplus jetPRIME transfection reagent Polyplus in France.

### RNA sequencing

The differentially expressed gene (DEG) analysis was reported in our former study [24]. The results of GO enrichment analyses for DEGs in siSLC7A11-treated SUNE1 cells contrasted to those obtained for the negative control SUNE1 cells. Pathway analyses were performed using an R package “clusterProfiler.” Terms in GO and KEGG with *P* value < 0.05 were considered significantly enriched and were visualized using the R package “enrichplot” and “ggplot2”.

### CCK8 assay and colony formation

Cell viability and colony formation experiments were conducted using the CCK-8 assay (Bimike, USA) as well as the previously described methods in a 24 well plate [21].

### Apoptosis analysis by flow cytometry

A propidium iodide (PI)/Annexin V-FITC kit was used to detect apoptosis in SUNE1 cells grouped by the treatment of control, sorafenib alone, control combined coculture with tumor-specific T cells, and sorafenib combined coculture with tumor-specific T cells. Cells were plated in 6-well cell culture plates at 1 × 10^5^ cells/well and incubated to achieve adherence. The SUNE1 cells were treated by methods mentioned above and collected to accept flow cytometry tests. Flow cytometry samples were stained following the manufacturer’s protocol. Apoptotic NPC cells were identified, categorized, and quantified with a Beckman flow cytometer (CA, USA).

### qRT-PCR

The mRNA expression was assessed from whole-cell RNA as previously outlined [21]. The primers were as follows: β-actin, forward CTGGGACGACATGGAGAAAA; reverse AAGGAAGGCTGGAAGAGTGC; SLC7A11, forward CGTCCTTTCAAGGTGCCACT; reverse GGCAGATTGCCAAGATCTCAAG. EGFR, forward CAGACCGGACGACAGGC; reverse CCACCTCCTGGATGGTCTTT; GR, forward AAGAGCAGTGGAAGGACAGC；reverse CCTGTAGTGGCCTGCTGAAT. TAP1, forward TGTGACAAGGTTCCCACTGCTTAC; reverse GGCTGTGGCCTATGCAGTCA. FAF2, forward CGACCCCTGCAGGTTAATACA; reverse CATCGTTAAGTGCCTGGCTG.

### Western blot analysis

Western blotting was conducted in accordance with a previously established protocol [25], using the following antibodies: SLC7A11 (1:1000, ab175186, Abcam, 35-55kD; 1:1000, 26864-1-AP, Proteintech Group, 55kD; 1:1000, 382036, Zenbio, 55kD), EGFR (1:2000, 26864-1-AP, Proteintech Group), p-EGFR (1:1000, R24173, ZenBioScience), Flag (1:2000, 66008-4-Ig,Proteintech Group), GR (1:5000, 66904-1-Ig, Proteintech Group), TAP1 (1:3000, 11114-1-AP, Proteintech Group), FAF2 (1:3000, 16251-1-AP, Proteintech Group), MHC-I (1:2000, 15240-1-AP, Proteintech Group), SYVN1 (1:1000, 13473-1-AP, Proteintech Group), OS-9 (1:1000, 10061-1-AP, Proteintech Group), VCP (1:2000, 10736-1-AP, Proteintech Group), HERP (1:1000, 10813-1-AP, Proteintech Group), Ubiquitin (1:1000, #3936, CST), β-actin (1:3000, AF7018, Affinity), Tubulin (1:10000, AC021, Abclonal), Histone H3 (1:2000, 68345-1-Ig, Proteintech Group).

### Chromatin immunoprecipitation (CHIP)

Candidate binding sites for the GR and TAP1 near the TAP1 promoter start site were predicted using Jaspar and PROMO. Primer pair was designed for a CHIP assay targeting TAP1 promoter. The CHIP assay was conducted for GR and TAP1 in NPC cells using the Magna CHIP A/G kit (Thermo Fisher Scientific), following the manufacturer’s instructions. Sheared chromatin was immunoprecipitated using control Rabbit IgG and an anti-GR antibody (Proteintech, Wuhan, China) overnight at 4 °C. The interaction between GR or TAP1 was quantified using quantitative PCR with SYBR Green (Bimike, USA) and the CFX96 system (Bio-Rad).

### Immunoprecipitation (IP)

In brief, total proteins were extracted and quantified. A total of 1000 μg protein was incubated with 2.5 μg anti-EGFR, anti-Flag, or anti-IgG antibodies for 12 h at 4 °C. Beads were washed, eluted in sample buffer, and boiled for 10 min at 100 °C. Immune complexes were then subjected to western blot analysis, with anti-IgG serving as a negative control.

### Dual Luciferase Reporter Gene Experiment

The binding sites of transcription factor on the TAP1 promoters were analyzed using the CHIP-Altas, PROMO, and Jaspar website. Cells were seeded into 96-well plates and cultured overnight. After 48 h of plasmid transfection (GenScript), the dual luciferase reporter gene experiment was conducted using the Duo-Lite Luciferase Assay System from Vazyme, following the manufacturer’s protocol. The luminescence was measured using the Varioskan LUX instrument from Thermo Scientific.

### Immunofluorescence (IF)

FFPE sections were baked at 60 °C for 1 h to improve sample adhesion to the slide. And then, the FFPE sections were deparaffinized by fresh xylene twice for 10 min each at room temperature. Rehydration was performed with a series of grade EtOH washes (100%, 95%, 85%, 75%) for 5 min, respectively. EDTA antigen retrieval solution (50×) (Abcarta, PB002) was diluted with dH_2_O for epitope recovery. Heat slides in EDTA antigen retrieval solution (1×) until boiling, then sub-boil for 20 min (95–98 °C). PBS was used to wash sections twice for 3 min after cooling down. Wash buffer was then removed, and sections were blocked with peroxidase-blocking reagent for 15 min. While blocking, MHC-I antibody (Proteintech, 15240-1-AP) was diluted (dilution: 1:500) in antibody dilution buffer (Abcarta, PB001). Then, the diluted primary antibody was incubated for 1 h at 37°C in a humidified chamber, and secondary antibody incubation was performed for 30 min at 37°C by covering sections with 1-3 drops of HRP-conjugated goat anti-rabbit & mouse reagent (Abcarta, PK014). Signal amplification was achieved with 1× fluorophore 650-conjugated TSA amplification reagent for 5 min. PBS was used to wash sections twice for 3 min between each step. Slides were counterstained with DAPI and mounted with antifade mounting medium. Simultaneously, stained slides were scanned and imaged using the HEIDSTAR automatic digital fluorescent scanning system (v23.10.30). Acquired image files were subsequently opened and fused using the HALO image analysis platform (v3.6.4134.362). We established intensity thresholds to distinguish among strong, moderate, and weak fluorescence signals, calculating the percentage of MHC-I expression intensity across different cell membranes to assess differences between among groups.

### Immunoprecipitation-mass spectrometry (IP-MS)

Purified protein complex eluates were concentrated using IP beads (Bimake), resolved by SDS-PAGE, and stained with Coomassie Brilliant Blue (Beyotime). Stained bands that differed from the control were excised, subjected to in-gel reduction, alkylated, and digested with Trypsin (mass ratio 1:50) at 37 °C for 20 h. After desalination, the enzymatic hydrolysate is freeze-dried and redissolved in a 0.1% FA solution, stored at −20 °C for future use. A liquid is a 0.1% formic acid aqueous solution, while B liquid is a 0.1% formic acid acetonitrile aqueous solution (84% acetonitrile). After the chromatographic column is equilibrated with 95% liquid A, the sample is loaded into the Trap column through an automatic sampler. Collect peptide and peptide fragment mass to charge ratio by collecting 20 fragment maps (MS2 scan) after each full scan. Finally, the raw file of mass spectrometry testing was retrieved from the corresponding database using ProteomeDiscoverer1.4 software, and the identified protein results were obtained.

### Detection of glutathione (GSH) content

The intracellular GSH levels were measured using a Reduced Glutathione (GSH) Content Assay Kit (Solarbio, BC1175). Collect 5 million nasopharyngeal carcinoma cells after treatment, wash the cells twice with PBS, resuspend the cells with 200ul of reagent, perform ultrasound (200w, ultra 3 s, stop for 10 seconds, repeat 30 times), centrifuge 8000 *g* for 10 min, and collect the supernatant. Measure the absorbance of distilled water and samples at 412 nm, and then calculate the GSH content.

### Generation tumor-specific T cells

Production of tumor-peptide-specific T cells was adapted from Wang. Based on the manufacturer’s instructions and previous report [26], peripheral blood mononuclear cells (PBMCs) were obtained from the peripheral blood of healthy donors using the Ficoll-Hypaque method (tbdscience). For the generation of DCs, the isolated PBMCs were added to 10% FBS-1640 medium containing 50 ng/mL GM-CSF and 20 ng/mL IL-4 (PeproTech), and were cultured for 5 days. Cell differentiation was monitored using light microscopy. To promote DC maturation, 25 ng/mL interferon γ (IFN-γ; PeproTech) was added for incubation for 1 day and co-culture with SUNE1 cell lysates was performed for a period of 1 day. T cells were subjected to expansion in vitro by adding CD3/CD28 MACSiBead (Miltenyi, Germany) and 15 ng/mL IL-2, 5 ng/mL IL-7, and 10 ng/mL IL-15 (PeproTech) to PBMCs and by incubating for 8 days. To generate tumor-specific T cells, the prepared DCs and the expanded T cells were co-cultured at a 1:5 ratio for 5 days in the medium supplemented with IL-2, IL-7, and IL-15. Fresh medium and cytokines were replaced with the fresh medium every 2 days during the experimental period.

### Label-free Quantification Proteomics

Protein was extracted from cell samples using SDT lysis buffer (4% SDS, 100 mM DTT, 100 mM Tris-HCl pH 8.0). Protein (200 μg for each sample) digestion was performed with FASP method described by Wisniewski, Zougman et al. [27]. LC-MS/MS were performed on a Q-Exactive HF-X mass spectrometer coupled with Easy 1200 nLC (Thermo Fisher Scientific).The MS data were analyzed using Proteome Discoverer software version 2.4. The “LFQ intensity” of each protein in different samples was calculated as the best estimate, satisfying all of the pairwise peptide comparisons, and this LFQ intensity was almost on the same scale of the summed-up peptide intensities. The quantitative protein ratios were weighted and normalized by the median ratio in Proteome Discoverer software. Only proteins with fold change ≥1.5-fold and a p-value < 0.05 were considered for significantly differential expressions.

### Experiment of Label-free Phosphoproteomics

Cell samples were dissolved with 200 μL lysis buffer (4% SDS, 100 mM DTT, 150 mM Tris-HCl pH 8.0). The samples were boiled and further ultrasonicated. Digestion of protein was performed according to the FASP procedure described by Wisniewski, Zougman et al. [27]. For phosphopeptide enrichment, High-Select™ TiO2 Phosphopeptide Enrichment kit from Thermo Scientific were used for digested peptide mixtures from each sample. Liquid chromatography-mass spectrometry (nanoLC-MS/MS) was performed on a Q Exactive HF-X (Thermo Scientific) coupled with Easy nLC 1200 system for chromatographic separation.Mass spectrometry was performed on a nano electrospray ion source (nESI). The MS data were analyzed for data interpretation and protein identification against the homo database from Uniprot. The MS spectra were searched using MSFragger version 2.4 and FragPipe version 13.1 with mass calibration and parameter optimization enabled. Tryptic cleavage specificity was applied, along with variable methionine oxidation (M), variable protein N-terminal acetylation, variable phosphorylation on serine (S), threonine (T) and tyrosine (Y), and fixed carbamidomethyl cysteine modifications. The allowed peptide length and mass ranges were 6–50 residues and 500–5000 Da, respectively.

### Enzyme-linked immunosorbent assay (ELISA)

The transfected tumor cells were co-cultured with activated T cells for 24 h. The culture supernatant was collected to detect IFN-γ by the ELISA kit (Elabscience). The results were analyized by plate reader with SoftMax® Pro 7 software, version 7.1.0 (MolecularDevices). All experiments were performed as per the manufacturer’s instructions.

### Animal studies

For the subcutaneous tumor model, 6-10B (5 × 10^6^ cells) and SUNE1(5 × 10^6^ cells) were suspended in a 100 μl mixed of normal saline and Matrigel (1:1) and subcutaneously injected into male NCG mices and nude mices respectively. When the tumor volume reached 50-100 mm^3^, we numbered the mices one by one, and then used a random number table to group them. The mices were randomly assigned to four groups (n = 6 per group in the NCG mice subcutaneous tumor models, n = 5 per group in the nude mice subcutaneous tumor models), which were named Ctrl group, Ctrl + T cell group, sorafenib group and sorafenib + T cell group. The sorafenib group was treated with sorafenib (15 mg/kg, administered via oral gavage; 5 days of continuous treatment followed by a pause for 2 days and a new round of treatment; conducted for two cycles), while the Ctrl group received the solvent. T cell group received a tail vein injection of T cells (5 × 10^7^ cells) after one week of Sorafenib treatment. Tumor size and mouse weight were monitored every two days. After 3 weeks, the mice were euthanized, and the tumors were collected. The tumor volume was calculated using the formula: V = 1/2 ×length × width^2^. Subsequently, all tumors were excised, weighed, fixed in 10% neutral buffered formalin for 24 h, the paraffin sections, and subjected to hematoxylin/eosin staining and immunohistochemistry for MHC-I.

### Statistical methods

Statistical analyses were conducted using the log-rank test, Chi-square test, multivariate Cox regression analysis, and Student’s t-test as appropriate, utilizing SPSS for Windows (18.0; SPSS, Inc.) and GraphPad Prism (Prism 8.0; GraphPad Software Inc.) software packages. The *P* value less than 0.05 was considered statistically significant. Error bars in all Figs represent the standard deviation. Significance levels were indicated as follows: *P < 0.05, **P < 0.01, ***P < 0.001 based on two-tailed t-tests.

## Results

### EGFR stabilizes SLC7A11 protein expression in NPC via a kinase-independent mechanism

**Fig. 1:**
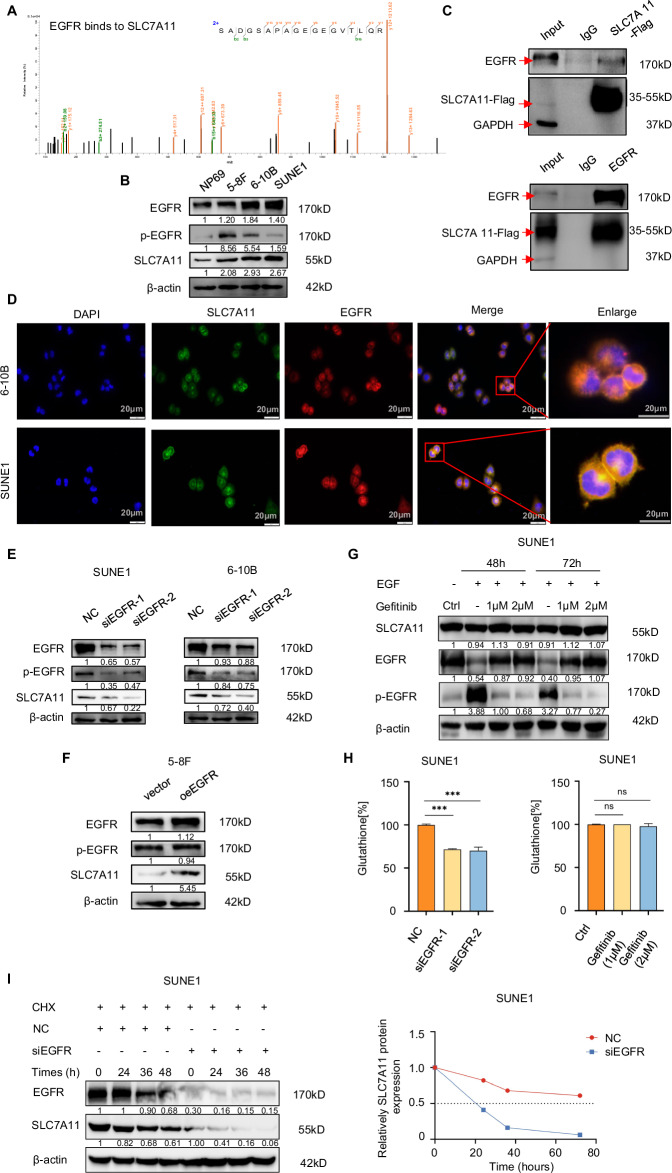
EGFR stabilizes SLC7A11 protein expression in NPC via a kinase-independent mechanism. **A** To identify EGFR-interacting proteins, we employed immunoprecipitation (IP) followed by mass spectrometry (MS), where the SLC7A11 peak was discerned. **B** In the NP69 cell line and NPC cell lines (5-8F, 6-10B, SUNE1), the protein expression of EGFR, p-EGFR, and SLC7A11 was confirmed via Western blotting. β-actin served as a loading control. **C** Co-immunoprecipitation (Co-IP) experiments validated the interaction between EGFR and SLC7A11 in SUNE1 cells. **D** Immunofluorescence microscopy revealed the co-localization of EGFR and SLC7A11 in SUNE1 and 6-10B cells (scale bar: 20 μm). **E**, **F**. The impact of either EGFR knockdown or overexpression on p-EGFR and SLC7A11 protein levels was confirmed using Western blotting, with β-actin as the loading control. **G** Immunoblotting was used to analyze SLC7A11, and both total and phosphorylated EGFR in SUNE1 cells. These cells were incubated with or without EGF (50 ng/ml) or gefitinib (1 or 2 μM) for the specified durations. **H** In SUNE1 cells, glutathione levels were assessed post-EGFR knockdown (left) or after gefitinib treatment (right). **I** In SUNE1 cells transfected with control or EGFR siRNAs for 48 h and subsequently exposed to cycloheximide (CHX, 100 μg/ml), immunoblot analysis was conducted for SLC7A11 (left). The SLC7A11/β-actin band intensity ratios revealed the protein half-life of SLC7A11 (right).

Through immunoprecipitation (IP) and mass spectrometry (MS), we identified proteins interacting with EGFR, with a particular interest in SLC7A11 (Fig. 1A) due to its role in regulating CD8^+^T lymphocyte recruitment [28]. In subsequent in vitro experiments, we aimed to explore the regulatory relationship between EGFR and SLC7A11. Comparing EGFR and SLC7A11 expression levels in human immortalized nasopharyngeal epithelial cells NP69 to NPC cell lines revealed higher expression in NPC cell lines than in NP69 cells. And consistent trends of EGFR and SLC7A11 have been observed in different NPC cells (Fig. 1B). The SUNE1 and 6-10B cells displayed higher EGFR coupled with elevated SLC7A11. The binding of SLC7A11 and EGFR in the SUNE1 cell line was confirmed through IP (Fig. 1C). Immunofluorescence staining revealed co-localization (yellow) of EGFR (red) and SLC7A11 (green) in SUNE1 and 6-10B cell lines (Fig. 1D). Knocking down EGFR accompanied by decreased p-EGFR led to declinded SLC7A11 protein levels (Fig. 1E), while overexpressing EGFR with no significant changes in p-EGFR resulted in increased SLC7A11 protein expression (Fig. 1F).

EGFR interacts with SLC7A11, preventing EGFR intracellular segment dimerization and thereby stabilizing SLC7A11 protein independently of kinase activity [15]. To investigate whether EGFR regulates the SLC7A11 expression in NPC in a manner linked to tyrosine kinase activation, SUNE1 and 6-10B cells were pre-treated with epidermal growth factor (EGF) and then exposed to varied concentrations of EGFR-tyrosine kinase inhibitor gefitinib. While 1 or 2 μM gefitinib effectively blocked EGF-induced EGFR activation, the abundance of SLC7A11 had minor decline in SUNE1 and 6-10B cells (Fig. 1G and Supplementary Fig. 1A). Despite the known role of SLC7A11 in importing cystine for glutathione synthesis, we observed that EGFR knockdown inhibited intracellular glutathione synthesis, whereas treatment with the TKI gefitinib did not affect it (Fig. 1H and Supplementary Fig. 1B). This suggests that EGFR promotes SLC7A11 expression and activity mainly independently of its kinase activity. Notably, SLC7A11 protein exhibited a half-life of >70 h and ~60 h in SUNE1 and 6-10B cells, respectively, which was reduced to ~20 h and ~30 h, after EGFR knockdown (Fig. 1I and Supplementary Fig. 1C), underscoring the role of EGFR in stabilizing SLC7A11 protein.

### SLC7A11 shields NPC cells from T cell-induced cytotoxicity

**Fig. 2:**
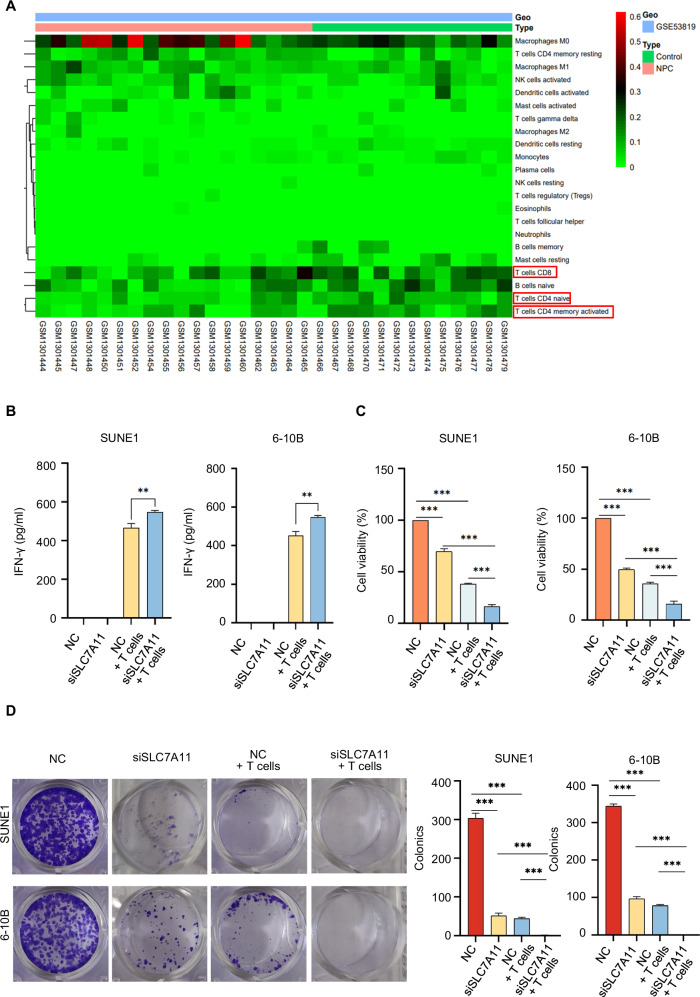
SLC7A11 shields NPC cells from T cell-induced cytotoxicity. **A**.Heatmap of predicted immune cell infiltrate levels in the GSE53819 dataset which with higher SLC7A11 levels in NPC tissues. **B** IFN-γ production by T cells was measured by ELISA as indicated treatment. **P < 0.01. The one-way analysis of variance (ANOVA). Error bars, mean ± *SD*. **C** CCK8 assays were performed to detect the cell viability as indicated treatment. ***P < 0.001. The one-way analysis of variance (ANOVA). Error bars, mean ± *SD*. **D** Colony formation assays were conducted to determine the colony numbers of SLC7A11-downregulated SUNE1 and 6-10B cells which were co-cultured with T cells or not. ***P < 0.001. The one-way analysis of variance (ANOVA). Error bars, mean ± *SD*.

Analysis of the GSE53819 dataset revealed lower various T cell infiltration, including CD8^+^T cells, T cells CD4 naïve, and T cells CD4 memory activated (Fig. 2A and Supplementary Fig. 2), in NPC tissues with higher SLC7A11 levels compared to controls [29]. To investigate whether SLC7A11 enables NPC cells to evade T cell immune surveillance, we downregulated SLC7A11 in NPC cells and performed co-cultures with human T cells. Results showed increased IFN-γ production (Fig. 2B), along with reduced cell survival (Fig. 2C) and diminished colony formation (Fig. 2D) in SUNE1 and 6-10B cells with downregulated SLC7A11when co-cultured with human T cells, in contrast to the other groups.

### Glucose-dependent properties of high SLC7A11 expression inhibit TAP1 transcription through GR, weakening MHC-I membrane expression in NPC cells

**Fig. 3:**
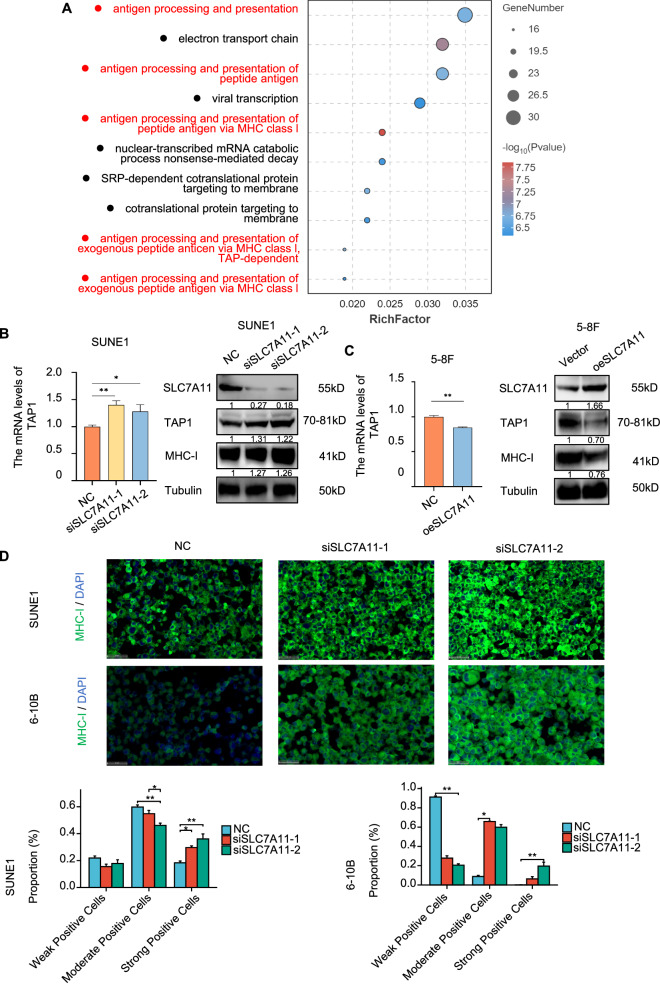
High SLC7A11 expression inhibit TAP1, weakening MHC-I membrane expression in NPC cells. **A** Bubble chart of GO enrichment analysis for upregulated genes in SUNE1 cells post-SLC7A11 knockdown. **B** Left: qPCR analysis of TAP1 mRNA post-SLC7A11 knockdown. Right: Western blot verifying effects of SLC7A11 knockdown on TAP1 and MHC-I protein expression. Tubulin were used as loading controls. **C** Left: qPCR analysis of TAP1 mRNA following SLC7A11 overexpression. The effect of overexpressed SLC7A11 on the protein expression of TAP1 and MHC-I were verified by Western blot. Tubulin were used as loading controls. **D** Immunofluorescence assessment of MHC-I in SUNE1 and 6-10B cells post-SLC7A11 knockdown. Scale bars, 50 μm. *P < 0.05, **P < 0.01. The one-way analysis of variance (ANOVA) and Tukey’s post hoc test in SUNE1 cells, Kruskal-Wallis Test and Dunn’s test in 6-10B cells. Error bars, mean ± *SEM*.

**Fig. 4:**
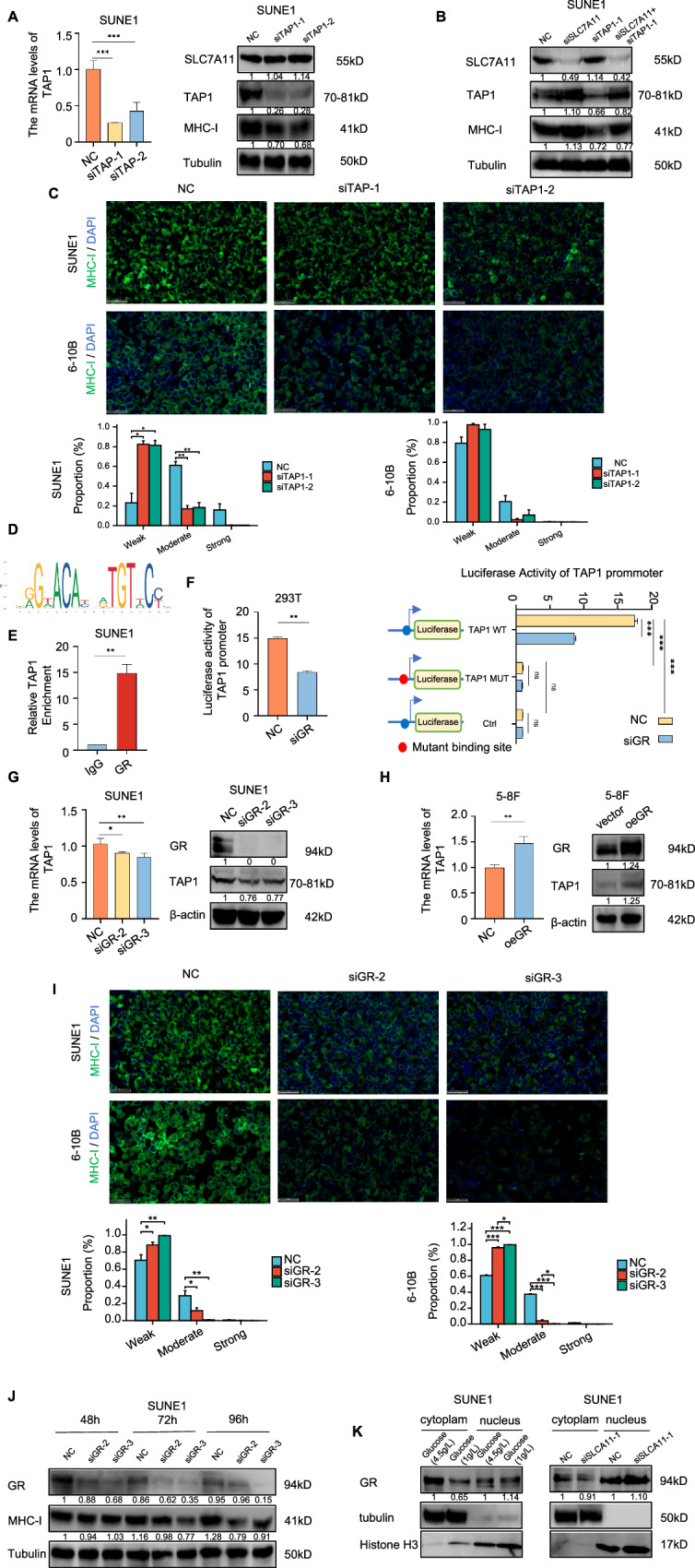
Glucose-dependent properties of high SLC7A11 expression inhibit TAP1 transcription through GR, weakening MHC-I membrane expression in NPC cells. **A** Left: qPCR confirmation of TAP1 mRNA knockdown using siTAP1-1/2. Right: The effect of TAP1 knockdown on the protein expression of SLC7A11, TAP1 and MHC-I were verified by Western blot. Tubulin were used as loading controls. **B** Immunoblot assessment of SLC7A11, TAP1, and MHC-I in SUNE1 cells post-transfection: control, siSLC7A11, siTAP1, or combined siSLC7A11 and siTAP1. Tubulin were used as loading controls. **C** Immunofluorescence assessment of MHC-I in SUNE1 and 6-10B cells post-TAP1 knockdown. Scale bars, 50 μm. *P < 0.05, **P < 0.01. The Welch one-way ANOVA test and Games-Howell test in SUNE1 cells, Kruskal–Wallis test and Dunn’s test in 6-10B cells. Error bars, mean ± *SEM*. **D** DNA binding domain sequence for GR. **E** ChIP assay confirmed the binding of GR to the TAP1 promoter in SUNE1 cells. Student’s two-tailed *t* test, **P < 0.01. **F** Luciferase reporter assay further confirmed direct binding of GR to TAP1 promoter in 293T cells. Student’s two-tailed *t* test, **P < 0.01, ***P < 0.001. **G** Left: qPCR analysis of TAP1 mRNA post-GR knockdown. Right: The effect of GR knockdown on the protein expression of TAP1 was verified by Western blot. β-actin were used as loading controls. **H** Left: qPCR analysis of TAP1 mRNA following GR overexpression. Right: The effect of GR overexpression on the protein expression of TAP1 was verified by Western blot. β-actin were used as loading controls. **I** Immunofluorescence assessment of MHC-I in SUNE1 and 6-10B cells post-GR knockdown. Scale bars, 50 μm. *P < 0.05, **P < 0.01, ***P < 0.001. The one-way analysis of variance (ANOVA) and Tukey’s post hoc test in SUNE1 and 6-10B cells. Error bars, mean ± *SEM*. **J** Immunoblot assessment of MHC-I protein expression in SUNE1 cells at specified times post-GR knockdown. Tubulin were used as loading controls. **K** Left: Immunoblot assessment of GR location in SUNE1 cells subjected to high/low glucose concentrations at specified times. Tubulin was used as loading controls of cytoplasm. Histone H3 was used as loading controls of nucleus. Right: Immunoblot assessment of GR location in SUNE1 cells at specified times post-SLC7A11 downregulation. Tubulin was used as loading controls of cytoplasm. Histone H3 was used as loading controls of nucleus.

In SLC7A11-knockdown SUNE1 cells, combined with RNA-seq analysis, we focused on the antigen processing and presentation processes (Fig. 3A). The observed reduction in T cell infiltration in tumors could result from a shortage of tumor antigens available to recruit and activate T cells, thereby shielding tumor cells from recognition and subsequent attack [7]. Transporter associated with antigen processing 1 (TAP1), a critical component of the stable peptide MHC-I complex, plays a pivotal role in antigen presentation. Proteins are continuously converted to peptides by cytosolic proteasomes, which are subsequently loaded onto MHC-I molecules by TAP [30]. Knocking down SLC7A11 resulted in elevated TAP1 mRNA and protein levels, as well as an increase in MHC-I expression in SUNE1 and 6-10B cells (Fig. 3B and Supplementary Fig. 3A). Conversely, SLC7A11 overexpression led to decreased TAP1 mRNA, protein levels, and MHC-I expression (Fig. 3C). Fluorescence analysis demonstrated enhanced membrane localization of MHC-I (green) following SLC7A11 knockdown (Fig. 3D). While MHC-I protein levels were downregulated in TAP1-knockdown SUNE1 and 6-10B cells (Fig. 4A and Supplementary Fig. 3B), co-transfecting siSLC7A11 and siTAP1 into these cells revealed that TAP1 knockdown could counteract the MHC-I upregulation caused by SLC7A11 knockdown (Fig. 4B and Supplementary Fig. 3C). And the fluorescence analysis demonstrated decreased membrane localization of MHC-I (green) following TAP1 knockdown (Fig. 4C) underscoring the role of TAP1 in regulating MHC-I expression under the influence of SLC7A11 in SUNE1 and 6-10B cells. In summary, SLC7A11 modulates MHC-I expression through its effect on TAP1.

We then aimed to identify the transcription factors that regulate TAP1 expression under the control of SLC7A11. Candidate transcript factors of TAP1 (Supplementary Fig. 3D) were identified using CHIP-Altas (https://chip-atlas.org/) and PROMO (https://alggen.lsi.upc.es/cgi-bin/promo_v3/promo/promoinit.cgi?dirDB=TF_8.3). The glucocorticoid receptor (GR), a member of the nuclear hormone receptor family, is a crucial nuclear transcription factor known to translocate from the cytoplasm to the nucleus, influencing the expression of various genes [31]. We predicted the transcription factor-binding sites of GR to the promotor of TAP1 (Fig. 4D) using JASPAR (https://jaspar.genereg.net/matrix/MA0007.1/). Subsequently, we designed and constructed a TAP1 promoter expression vector and crafted primers using the predicted GR binding domain within the TAP1 promoter region (specifically at NC_000006.12: c32855704-32853704:1912-1928, with the sequence AGGATCAGCCTGTTCCT). CHIP qPCR experiments revealed GR pulled down the TAP1 promoter binding domain fragment (Fig. 4E). Dual luciferase experiments demonstrated reduced luciferase activity of the TAP1 promoter vector in 293 T cells following GR knockdown (Fig. 4F, left). We then mutated the binding sequence within the TAP1 promoter and repeated the dual luciferase experiment. As anticipated, post-mutation, firefly fluorescence was undetectable (Fig. 4F, right). GR knockdown led to a reduction in TAP1 mRNA and protein expression in SUNE1 and 6-10B cells (Fig. 4G and Supplementary Fig. 3E), while GR overexpression elevated these levels (Fig. 4H). Furthermore, we observed a reduced membrane localization of MHC-I upon GR knockdown (Fig. 4I). Additionally, a time-gradient GR knockdown resulted in a notable decline in MHC-I protein levels, with the most pronounced effect observed at 96 h in SUNE1 and 6-10B cells (Fig. 4J and Supplementary Fig. 3F). These findings suggest that GR acts as a transcription factor to enhance TAP1 expression, consequently stabilizing the MHC-I protein in NPC.

High SLC7A11 expressed cancer cells show a glucose dependency to survive. High glucose can generate NADPH via the pentose phosphate pathway for reducing cystine to cysteine [32]. We proposed that increased intracellular glucose levels as a result of SLC7A11 might hinder nuclear entry of GR. Nuclear-plasma protein separation indicated that under high glucose conditions, GR protein predominantly localized to the cytoplasm rather than the nucleus in contrast to low glucose conditions in SUNE1 and 6-10B cells (Fig. 4K, left and Supplementary Fig. 3G). However, upon SLC7A11 knockdown, GR exhibited a preference for nuclear localization compared to the control group in SUNE1 and 6-10B cells (Fig. 4K, right and Supplementary Fig. 3H). Fluorescence analysis also demonstrated enhanced nuclear localization of GR (red), and membrane localization of MHC-I (green) following SLC7A11 knockdown (Supplementary Fig. 3I).

### SLC7A11 enhances FAF2 expression, activating the ERAD pathway implicated in the ubiquitin-mediated degradation of MHC-I

**Fig. 5:**
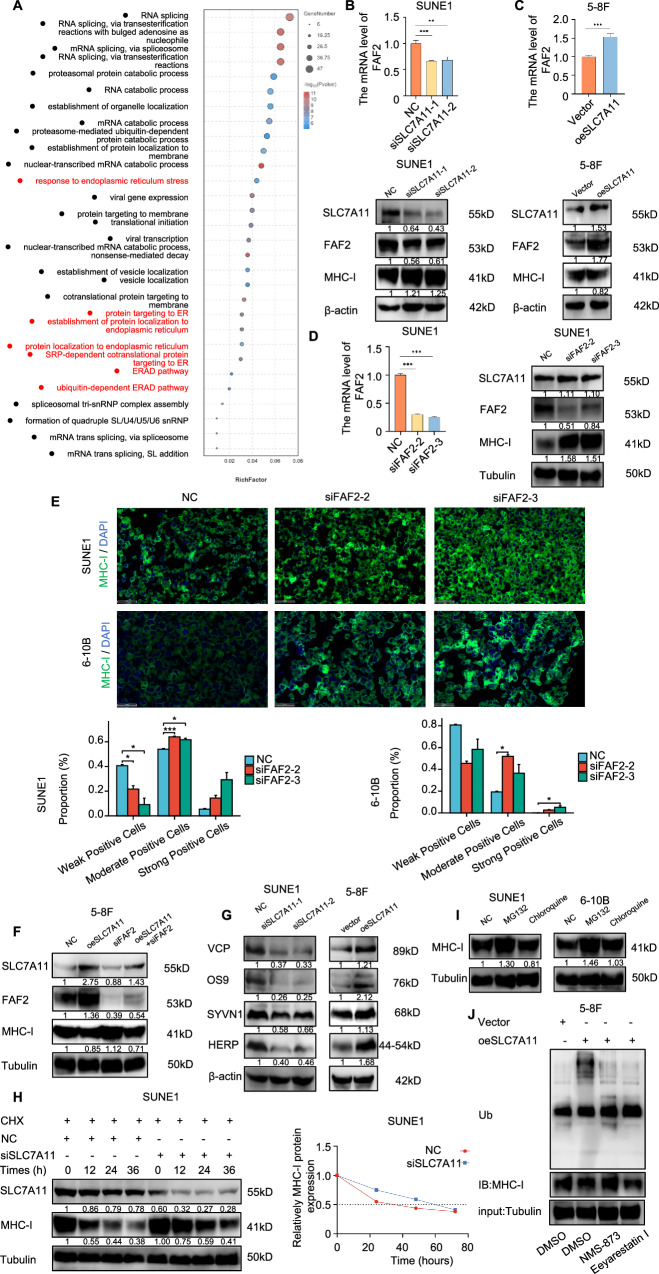
SLC7A11 enhances FAF2 expression, activating the ERAD pathway implicated in the ubiquitin-mediated degradation of MHC-I. **A** Bubble chart of GO enrichment analysis for downregulated genes in SUNE1 cells post-SLC7A11 knockdown. **B** Upper: qPCR analysis of FAF2 mRNA post-SLC7A11 knockdown. Lower: the effect of knockdown SLC7A11 on the protein expression of FAF2 and MHC-I were verified by Western blot. β-actin were used as loading controls. **C** Upper: qPCR analysis of FAF2 mRNA following SLC7A11 overexpression. Lower: the effect of SLC7A11 overexpression on the protein expression of FAF2 and MHC-I were verified by Western blot. β-actin were used as loading controls. **D** Left: qPCR confirmation of FAF2 mRNA knockdown using siFAF2-2/3. Right: the effect of FAF2 knockdown on the protein expression of SLC7A11 and MHC-I were verified by Western blot. Tubulin were used as loading controls. **E** Immunofluorescence assessment of MHC-I in SUNE1 and 6-10B cells post-FAF2 knockdown. Scale bars, 50 μm. *P < 0.05, ***P < 0.001. The Welch one-way ANOVA test and Games-Howell test in SUNE1 cells, Kruskal–Wallis Test and Dunn’s test in 6-10B cells. Error bars, mean ± *SEM*. **F** Immunoblot assessment of SLC7A11, FAF2, and MHC-I in SUNE1 cells post-transfection: control, siSLC7A11, siFAF2, or combined siSLC7A11 and siFAF2. Tubulin were used as loading controls. **G** Left: Immunoblot of ERAD pathway proteins (VCP, OS9, SYVN1, HERP) in SUNE1 cells post-SLC7A11 knockdown (left), and in 5-8F cells post-SLC7A11 overexpression (right). **H** Left: Immunoblot of MHC-I in SUNE1 cells transfected with control or FAF2 siRNA for 36 h, then treated with cycloheximide (CHX, 100 μg/ml) for specified durations. Tubulin were used as loading controls. Right: The half-life of the MHC-I protein was calculated. **I** Immunoblot assessment of MHC-I in SUNE1 and 6-10B cells under control, MG132, or Chloroquine treatments. Tubulin were used as loading controls. **J** In 5-8F cells overexpressing SLC7A11, IP complexes of MHC-I were immunoblotted for ubiquitination (Ub) under control, VCP inhibitors (NMS-873, 10 μM, MERCK, SML1128), or ERAD inhibitors (Eeyarestatin I, 1.25 μM, MERCK, E1286) treatments. β-actin was used as loading controls.

In addition to its role in TAP1-dependent transcriptional regulation, differentially expressed genes (DEGs) after SLC7A11 knockdown in SUNE1 cells were primarily enriched in processes related to the endoplasmic reticulum (ER) (Fig. 5A). Among these processes, the ER-associated protein degradation (ERAD) signaling pathway, which is involved in the ubiquitin-mediated degradation of MHC-I [33], caught our attention. We focused on *FAF2*, a DEG in the ERAD signaling pathway, following SLC7A11 knockdown in SUNE1 cells. In the SLC7A11 knockdown group, FAF2 mRNA and protein levels were reduced, while MHC-I levels were elevated compared to the control group in SUNE1 and 6-10B cells (Fig. 5B and Supplementary Fig. 4A). Conversely, SLC7A11 overexpression led to increased FAF2 mRNA and protein levels in 5-8F cells (Fig. 5C). Further experiments revealed that FAF2 knockdown had no effect on SLC7A11 protein levels but increased MHC-I protein expression in SUNE1 and 6-10B cells (Fig. 5D and Supplementary Fig. 4B). Additionally, we observed an increased membrane localization of MHC-I (green) in the FAF2 knockdown group (Fig. 5E). In 5-8F cells, overexpressed SLC7A11 combined with FAF2 downregulation reversed the inhibitory effect of SLC7A11 on MHC-I (Fig. 5F). These findings suggest elevated SLC7A11 can also suppress membrane MHC-I expression in NPC cells through FAF2. FAF2, an endoplasmic reticulum membrane-associated protein, participates in the ubiquitin-dependent degradation of substrates on the endoplasmic reticulum membrane. It can also act as a VCP adapter, reducing the VCP threshold and facilitating ERAD [34]. We further investigated SLC7A11’s impact on the ERAD pathway. SLC7A11 knockdown significantly reduced the expression levels of core molecules in the ERAD pathway, such as VCP, OS9, SYVN1, and HERP in SUNE1 and 6-10B cells (Fig. 5G left, and Supplementary Fig. 4C). Conversely, overexpression of SLC7A11 elevated their expression levels (Fig. 5G right), suggesting that elevated SLC7A11 might activate the ERAD pathway in NPC cells.

Further analysis revealed that SLC7A11 knockdown considerably extended the half-life of MHC-I protein from almost ~35 h to ~60 h in SUNE1 cells (Fig. 5H), and from almost ~25 h to ~65 h in 6-10B cells (Supplementary Fig. 4D). After MG132 treatment, we observed a substantial accumulation of MHC-I, signifying its degradation mainly via the proteasome pathway rather than autophagic degradation pathway (Fig. 5I). We next probed SLC7A11’s impact on MHC-I ubiquitination. Overexpressed SLC7A11 increased MHC-I ubiquitination, whereas VCP inhibitor (NMS-873, a noncompetitive allosteric inhibitor of p97 [35]) and the ERAD inhibitor (Eeyarestatin I, which inhibits some deubiquitination reactions associated with the dislocase p97 [36]) normalized its ubiquitination level (Fig. 5J). These findings suggest that SLC7A11 potentially facilitates MHC-I degradation through the ubiquitination-dependent ERAD pathway.

### Association among the EGFR, MHC-I, GR, TAP1 and FAF2 expressions, CD8^+^ T cells infiltration, and clinicopathological characteristics of NPC

**Fig. 6:**
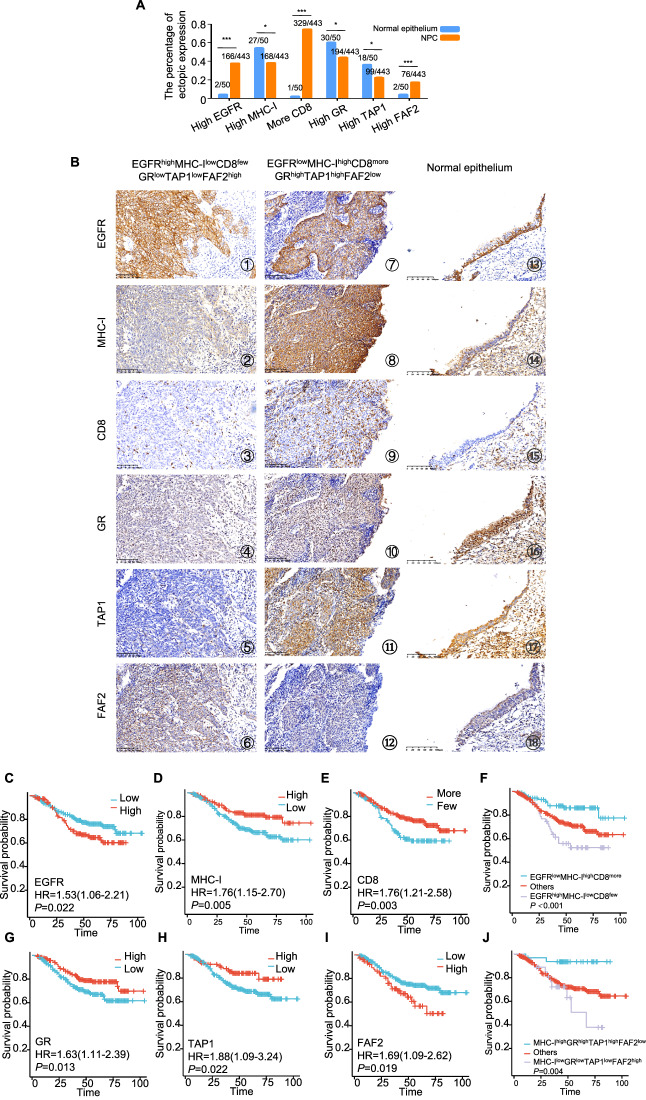
Association among the EGFR, MHC-I, GR, TAP1 and FAF2 expressions, CD8^+^ T cells infiltration, and clinicopathological characteristics of NPC. **A** Immunohistochemical staining revealed expression levels of EGFR, MHC-I, GR, TAP1, and FAF2, and the infiltration level of CD8^+^T cells in normal epothelium and NPC tissues. Chi-square test, statistically significant difference. *P < 0.05, ***P < 0.001. **B** Immunohistochemical staining revealed expression levels of EGFR, MHC-I, GR, TAP1, and FAF2, and the infiltration level of CD8^+^T cells in NPC tissues and normal epothelium tissues. EGFR (①, ⑦ and ⑬) and MHC-I (②, ⑧ and ⑭) proteins predominantly localized to the cell membranes. The dominant expression of EGFR in normal nasopharyngeal mucosal tissue was located near the basal side (⑬). CD8^+^T cells were also assessed in all samples (③, ⑨ and ⑮). GR protein was mainly localized in the cell nucleus (④, ⑩ and ⑯). TAP1 (⑤, ⑪ and ⑰) and FAF2 (⑥, ⑫ and ⑱) proteins primarily localized in the cell cytoplasm. Fig.s ①-⑥ depict a NPC case with EGFR^high^MHC-I^low^CD8^few^GR^low^TAP1^low^FAF2^high^ expression. Figs ⑦-⑫ illustrate a NPC case with EGFR^low^MHC-I^high^CD8^more^GR^high^TAP1^high^FAF2^low^ expression. Figs ⑬-⑱ showed a normal nasopharyngeal mucosal tissue with EGFR expression near the basal side, moderate MHC-I, no CD8^+^ T cells infiltration, strong GR and TAP1 expression, and weak FAF2. (Scale bars, 100 μm). C to J. Kaplan–Meier survival curves for 443 NPC patients, categorized by expressions of EGFR (**C**), MHC-I (**D**), CD8^+^T cells (**E**), combined EGFR, MHC-I, and CD8^+^T cells (**F**), GR (**G**), TAP1 (**H**), FAF2 (**I**), and a combination of MHC-I, GR, TAP1, and FAF2 (**J**). *P*-values were calculated using the log-rank test. Error bars represent mean ± *SD*.

The study examined the localization and expression of various markers in nasopharyngeal mucosal tissues and NPC tissues, and assessed their correlation with clinicopathological characteristics. We found that the expression of high EGFR, more CD8^+^ T cells infiltration, and high FAF2 was more likely to occur in NPC tissues than nasopharyngeal mucosal tissues. But the expression of high MHC-I, high GR and high TAP1 was easier to be observed in nasopharyngeal mucosal tissues rather than the NPC tissues (Fig. 6A and Fig. 6B ⑬-⑱). The results also shown that EGFR was predominantly localized in the cell membrane and cytoplasm (Fig. 6B ① and ⑦), and the dominant expression of EGFR in normal nasopharyngeal mucosal tissue was located near the basal side (⑬), while MHC-I was primarily observed in the cell membrane (Fig. 6B ② and ⑧). CD8^+^ T cell infiltration varied across different tissues (Fig. 6B ③ and ⑨). GR predominantly localized in the nucleus (Fig. 6B ④ and ⑩). TAP1 (Fig. 6B ⑤ and ⑪) and FAF2 (Fig. 6B ⑥ and ⑫) were primarily observed in the cytoplasm. The correlation analysis revealed several significant associations with clinicopathological characteristics: The findings are detailed in Table 1. Elevated expression of MHC-I (*P* = 0.005), TAP1 (*P* = 0.041), and increased CD8^+^T cell infiltration (*P* = 0.039) was predominantly observed in the lower T group. Elevated EGFR levels (*P* < 0.001, *P* = 0.019), reduced MHC-I (*P* = 0.045, *P* = 0.001), decreased CD8^+^T cell infiltration (*P* = 0.012, *P* = 0.003), decreased GR (*P* = 0.019, *P* = 0.029), and lower TAP1 (P = 0.013, P = 0.006) were associated with distant metastases and poorer survival in NPC patients. Higher FAF2 (*P* = 0.025) was associated with distant metastases in NPC patients. Reduced MHC-I (*P* = 0.004) and TAP1 (*P* = 0.002) levels correlated with advanced clinical stages. However, there was no significant correlation with age and gender. These findings provide insights into the relationships between these markers and clinicopathological characteristics in NPC, shedding light on potential prognostic factors and therapeutic targets.

**Table 1:** Analysis of the association between EGFR, MHC-I, CD8, GR, TAP1, and FAF2 expression and clinicopathological features of NPC (n = 443).

Clinicopathological features (n)	EGFR			MHC-I			CD8^+^T			GR			TAP1			FAF2		
	Low (%)	High (%)	*P*-value	Low (%)	High (%)	*P*-value	Few (%)	More(%)	*P*-value	Low (%)	High (%)	*P*-value	Low (%)	High (%)	*P*-value	Low (%)	High (%)	*P*-value
**Age (years)**																		
≤55 (*n* = 337)	212 (62.9)	125 (37.1)	0.818	209 (62.0)	128 (38.0)	1.000	83 (24.6)	254 (75.4)	0.373	190 (56.4)	147 (43.6)	0.911	262 (77.7)	75 (22.3)	1.000	277 (82.2)	60 (17.8)	0.558
> 55 (n = 106)	65 (61.3)	41 (38.7)		66 (62.3)	40 (37.7)		31 (29.2)	75 (70.8)		59 (55.7)	47 (44.3)		82 (77.4)	24 (22.6)		90 (84.9)	16 (15.1)	
**Gender**																		
Male (*n* = 318)	199 (62.6)	119 (37.4)	1.000	204 (64.2)	114 (35.8)	0.159	78 (24.5)	240 (75.5)	0.398	181 (56.9)	137 (43.1)	0.671	251 (78.9)	67 (21.1)	0.313	257 (80.8)	61 (19.2)	0.092
Female (*n* = 125)	78 (62.4)	47 (37.6)		71 (56.8)	54 (43.2)		36 (28.8)	89 (71.2)		68 (54.4)	57 (45.6)		93 (74.4)	32 (25.6)		110 (88.0)	15 (12.0)	
**T stages**																		
T_1-2_ (*n* = 228)	150 (65.8)	78 (34.2)	0.169	127 (55.7)	101 (44.3)	0.005^*^	49 (21.5)	179 (78.5)	0.039^*^	123 (53.9)	105 (46.1)	0.339	168 (73.7)	60 (26.3)	0.041^*^	190 (83.3)	38 (16.7)	0.802
T_3-4_ (*n* = 215)	127 (59.1)	88 (40.9)		148 (68.8)	67 (31.2)		65 (30.2)	150 (69.8)		126 (58.6)	89 (41.4)		176 (81.9)	39 (18.1)		177 (82.3)	38 (17.7)	
**LN status**																		
No LNM (*n* = 37)	24 (64.9)	13 (35.1)	0.860	20 (54.1)	17 (45.9)	0.295	6 (16.2)	31 (83.8)	0.237	19 (51.4)	18 (48.6)	0.605	26 (70.3)	11 (29.7)	0.302	30 (81.1)	7 (18.9)	0.819
LNM (*n* = 406)	253 (62.3)	153 (37.7)		255 (62.8)	151(37.2)		108 (26.6)	298 (73.4)		230 (56.7)	176 (43.3)		318 (78.3)	88 (21.7)		337 (83.0)	69 (17.0)	
**Metastasis**																		
No (*n* = 372)	247 (66.4)	125 (33.6)	<0.001^***^	223 (59.9)	149 (40.1)	**0.045**	87 (23.4)	285 (76.6)	0.012^*^	200 (53.8)	172 (46.2)	0.019^*^	281 (75.5)	91 (24.5)	0.013^*^	315 (84.7)	57 (15.3)	0.025^*^
Yes (*n* = 71)	30 (42.3)	41 (57.7)		52 (73.2)	19 (26.8)		27 (38.0)	44 (62.0)		49 (69.0)	22 (31.0)		63 (88.7)	8 (11.3)		52 (73.2)	19 (26.8)	
**Clinical Stages**																		
I-II (*n* = 60)	40 (66.7)	20 (33.3)	0.567	27 (45.0)	33 (55.0)	0.004^**^	12 (20.0)	48 (80.0)	0.341	30 (50.0)	30 (50.0)	0.328	37 (61.7)	23 (38.3)	0.002^**^	53 (88.3)	7 (11.7)	0.272
III-IV (*n* = 383)	237 (61.9)	146 (38.1)		248 (64.8)	135 (35.2)		102 (26.6)	281 (73.4)		219 (57.2)	164 (42.8)		307 (80.2)	76 (19.8)		314 (82.0)	69 (18.0)	
**Survival status**																		
Alive (*n* = 328)	216 (65.9)	112 (34.1)	0.019^*^	189 (57.6)	139 (42.4)	0.001^**^	72 (22.0)	256 (78.0)	0.003^**^	174 (53.0)	154 (47.0)	0.029*	244 (74.4)	84 (25.6)	0.006^**^	278 (84.8)	50 (15.2)	0.084
Dead (*n* = 115)	61 (53.0)	54 (47.0)		86 (74.8)	29 (25.2)		42 (36.5)	73 (63.5)		75 (65.2)	40 (34.8)		100 (87.0)	15 (13.0)		89 (77.4)	26 (22.6)	

*Chi-square test, statistically significant difference (*P < 0.05, **P < 0.01, ***P < 0.001).

*H* high expression, *L* low expression, *LNM* lymph node metastasis.

The study found several significant correlations between the expressions of various markers in NPC tissues. Here are the key findings (Table 2): EGFR expression inversely correlated with CD8^+^ T cell infiltration (*r* = −0.302, *P* < 0.001) and TAP1 levels (*r* = −0.113, *P* = 0.017) in NPC. MHC-I expression in NPC showed a positive correlation with CD8^+^ T cell infiltration (*r* = 0.098, *P* = 0.039), GR (*r* = 0.229, *P* < 0.001), and TAP1 (*r* = 0.262, *P* < 0.001), but it had a negative correlation with FAF2 (*r* = −0.109, *P* = 0.022). Besides, TAP1 expression was positively correlated with the GR (*r* = 0.171, *P* < 0.001). In terms of survival analysis: NPC patients with higher EGFR levels (Fig. 6C, *P* = 0.022), decreased MHC-I (Fig. 6D, *P* = 0.005), or reduced CD8^+^T cell infiltration (Fig. 6E, *P* = 0.003) had shorter survival spans. Patients with NPC with specific marker profiles had significantly lower total survival times than other profiles. For example, patients with NPC having both high EGFR levels, low MHC-I levels, and few CD8^+^T cell infiltrations had significantly shorter survival times (Fig. 6F, *P* < 0.001). The patients with lower GR (Fig. 6G, *P* = 0.013), lower TAP1 (Fig. 6H, *P* = 0.022), or higher FAF2 (Fig. 6I, *P* = 0.019) had shorter survival times than patients with higher GR, higher TAP1 or lower FAF2, and patients having MHC-I^low^GR^low^TAP1^low^FAF2^high^ expression had significantly lower total survival times (Fig. 6J, *P* = 0.004).

**Table 2:** The pairwise association among expression of EGFR, MHC-I, CD8^+^T, GR, TAP1 and FAF2 in 443 cases of NPC.

	EGFR	MHC-I	CD8^+^T	GR	TAP1	FAF2
**EGFR**	1.000	0.848 (*r* = −0.009)	<0.01^**^(*r* = **−0.302)**	0.101 (*r* = 0.078)	0.017^*^(*r* = **−0.113)**	0.090 (*r* = 0.081)
**MHC-I**	0.848 (*r* = -0.009)	1.000	0.039^*^ **(*r*** = **0.098)**	<0.01^**^ **(*r*** = **0.229)**	<0.01^**^ **(*r*** = **0.262)**	0.022^*^ **(*r*** = **−0.109)**
**CD8**^**+**^**T**	<0.01^**^ **(*r*** = **−0.302)**	0.039^*^ **(*r*** = **0.098)**	1.000	0.195 (*r* = 0.062)	0.091 (*r* = 0.080)	0.482 (*r* = −0.033)
**GR**	0.101 (*r* = 0.078)	<0.01^**^ **(*r*** = **0.229)**	0.195 (*r* = 0.062)	1.000	<0.01^**^ **(*r*** = **0.171)**	0.745 (*r* = −0.015)
**TAP1**	0.017^*^ **(*r*** = **−0.113)**	**<0.01** ^**^(*r* = **0.262)**	0.091 (*r* = 0.080)	<0.01^**^ **(*r*** = **0.171)**	1.000	0.132 (*r* = −0.072)
**FAF2**	0.090 (*r* = 0.081)	0.022^*^ **(*r*** = **−0.109)**	0.482 (*r* = −0.033)	0.745 (*r* = −0.015)	0.132 (*r* = −0.072)	1.000

^*^Spearman’s rank correlation test, statistically significant difference (*P < 0.05, **P < 0.01).

Cox multivariate regression analysis revealed advanced clinical stages (*P* = 0.004), and the phenotype of EGFR^high^MHC-I^low^CD8^few^ (*P* = 0.001) were independent prognostic indicators of NPC patients, as tabulated in Table 3. Additionally, the prognosis of NPC patients was unaffected by EGFR, MHC-I, CD8^+^T, GR, TAP1, FAF2 and MHC-I/GR/TAP1/FAF2 (all *P* > 0.05). These findings highlight the prognostic significance of these markers in NPC, emphasizing the potential clinical relevance of understanding their interactions and expressions for patient outcomes.

**Table 3:** Summary of multivariate of Cox proportional regression for overall survival in 443 cases of NPC.

Parameter	B	SE	Wald	Sig.	Exp (B)	95.0% CI for Exp (B)	
						Lower	upper
**Clinical stages**	2.860	1.005	8.107	0.004^**^	17.463	2.438	125.073
**EGFR**	0.345	0.197	3.062	0.080	1.412	0.959	2.077
**MHC-I**	−0.373	0.222	2.815	0.093	0.689	0.446	1.065
**CD8**^**+**^**T**	−0.218	0.249	0.768	0.381	0.804	0.494	1.310
**GR**	−0.376	0.197	3.643	0.056	0.686	0.466	1.010
**TAP1**	−0.297	0.285	1.086	0.297	0.743	0.424	1.300
**FAF2**	0.306	0.228	1.803	0.179	1.358	0.869	2.122
**EGFR/ MHC-I /CD8**^**+**^**T**	−0.553	0.173	10.222	0.001^**^	0.575	0.410	0.807
**MHC-I /GR/TAP1/FAF2**	0.068	0.371	0.033	0.855	1.070	0.517	2.212

*CI* confidence interval, *LNM* lymph node metastasis, *NPC* nasopharyngeal carcinoma.

multivariate analysis of Cox regression, **P < 0.01.

### Sorafenib targets SLC7A11 to induce NPC Cells to be killed by T-cells in vitro and in vivo

**Fig. 7:**
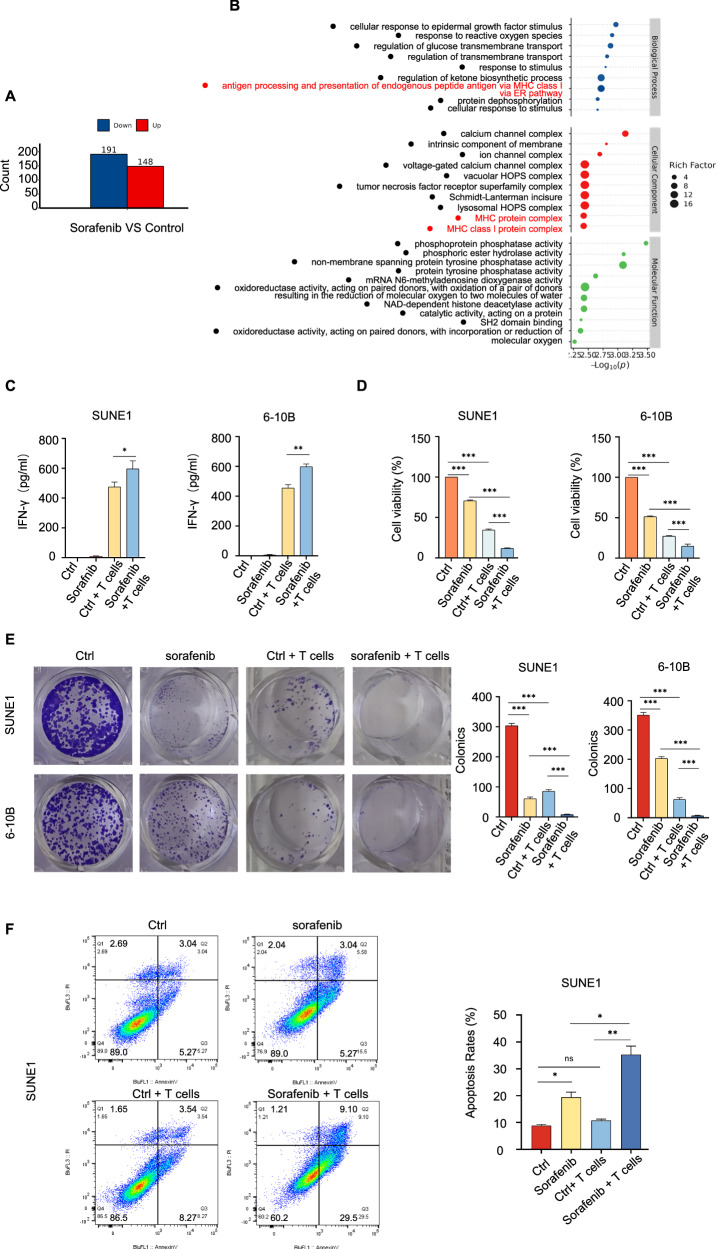
Sorafenib targets SLC7A11 to induce NPC Cells to be killed by T-cells in vitro. **A** Differentially expressed proteins (DEPs) in SUNE1 cells post-sorafenib treatment versus control. **B** Bubble chart of GO enrichment analysis for DEPs in SUNE1 cells post-sorafenib treatment. **C** IFN-γ production by T cells, measured using ELISA after specified treatments. *P < 0.05, **P < 0.01. The one-way analysis of variance (ANOVA). Error bars, mean ± *SD*. **D** CCK8 assays determined cell viability post specified treatments. ***P < 0.001. The one-way analysis of variance (ANOVA). Error bars, mean ± *SD*. **E** Colony formation assays measured colony counts in SUNE1 cells treated with sorafenib/control, and co-cultured with or without T cells. ***P < 0.001. The one-way analysis of variance (ANOVA). Error bars, mean ± *SD*. **F** SUNE1 cells, treated with or without sorafenib, were co-cultured with or without activated T cells. Apoptotic cells were analyzed by flow cytometry using Annexin V/ PI staining. Columns, means of three replicate determinations. The one-way analysis of variance (ANOVA). Error bars, mean ± SD. *P < 0.05, **P < 0.01.

Sorafenib, an FDA-approved novel oral multi-target tyrosine kinase inhibitor, is primarily used to treat tumors, including hepatocellular carcinoma and renal cell carcinoma [37]. Sorafenib can restrict cystine absorption through targeting SLC7A11 [38]. Earlier studies indicated that SLC7A11 predominantly functioned as an amino acid transporter, regulating intracellular metabolism and influencing tumor immune responses [18]. We put forward previously that an impaired antigen presentation process attributed to SLC7A11 in NPC cells. And sorafenib limited GSH synthesis in NPC cells [24]. Thus, the study investigated the potential therapeutic effects of sorafenib, a multi-target tyrosine kinase inhibitor, in NPC, focusing on its ability to target SLC7A11 and its impact on T-cell recognition and cytotoxicity. The findings are as follows: Sorafenib treatment resulted in proteomic alterations, with the downregulation of 191 proteins and upregulation of 148 proteins in SUNE1 cells (Fig. 7A). Biological enrichment analysis revealed that sorafenib treatment affected endogenous peptide processing and the MHC class I process complex (Fig. 7B). No angiogenesis-related pathways were identified. Despite we observed the cellular response enrichment to epidermal growth factor stimulation, no changes in the phosphorylation of Raf/MEK/ERK pathway was detected (Supplementary Table 1). ELISA results showed that sorafenib treatment in combination with T-cells co-cultivation led to the highest IFN-γ levels, indicating an ability to activate T cells (Fig. 7C). Sorafenib in combination with T-cell co-cultivation resulted in the most substantial reduction in NPC cell survival (Fig. 7D) and colony formation activity (Fig. 7E). Additionally, the combined sorafenib treatment and T-cell co-cultivation group displayed the highest levels of apoptosis of SUNE1 cells (Fig. 7F). These findings suggest that sorafenib, by targeting SLC7A11, has the potential to enhance T-cell recognition and cytotoxicity in NPC cells, making it a promising therapeutic option for NPC.

**Fig. 8:**
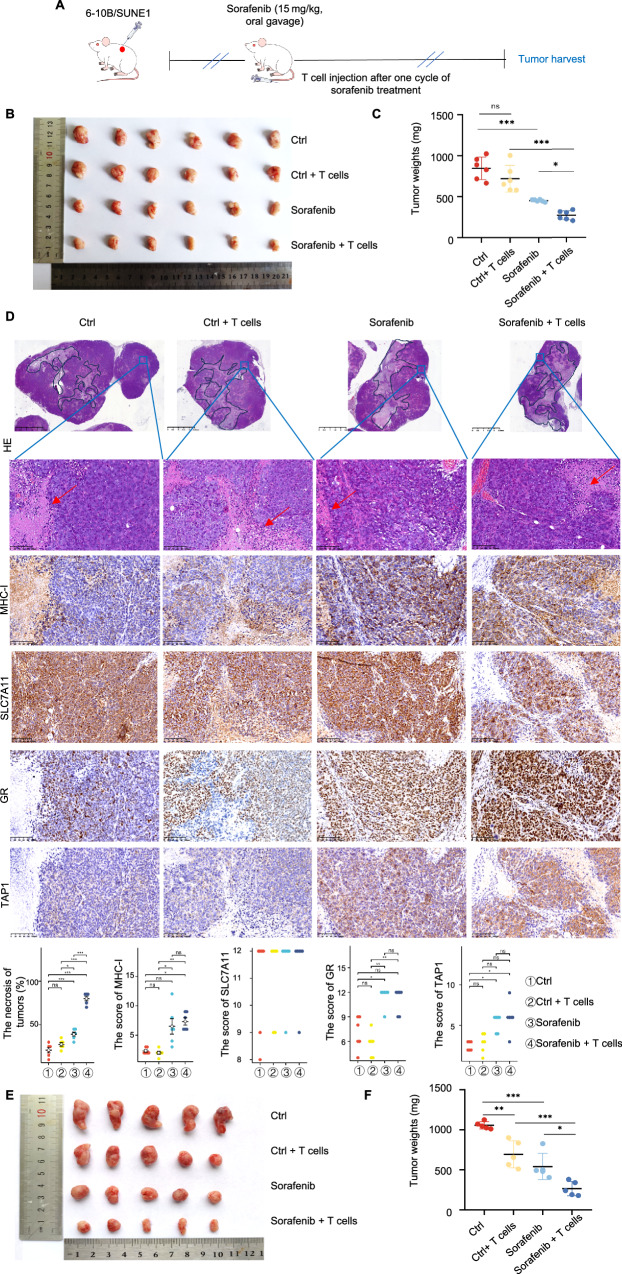
Sorafenib targets SLC7A11 to induce NPC Cells to be killed by T-cells in vivo. **A** Schematic of sorafenib administration and T-cell injection in NCG immunodeficient mouse and and nude mices xenograft models. **B** Tumor size comparison in NCG immunodeficient mouse receiving subcutaneous 6-10B cells injections with specific treatments. **C** Tumor weight comparison in NCG immunodeficient mouse following subcutaneous 6-10B cells injections with specified treatments. **D** Subcutaneous xenograft tumor sections stained with hematoxylin and eosin (H&E). Upper panel: the low magnification of subcutaneous xenografted tumor tissues (Scale bars: 2.5 mm). Black solid lines show necrotic core area. Lower panel: the higher magnification of subcutaneous xenografted tumor tissues (Scale bars: 100 µm). Red arrow indicates necrotic area. Immunohistochemical staining and statistical analysis the expression of MHC-I, SLC7A11, GR and TAP1 in tumor sections (Scale bars: 100 µm). **E** Tumor size comparison in the nude mices receiving subcutaneous SUNE1 cells injections with specific treatments. **F** Tumor weight comparison in the nude mices following subcutaneous SUNE1 cells injections with specified treatments.

The study utilized a combined approach involving NCG mouse and nude mices subcutaneous tumor formation and an adoptive T-cell model to investigate whether sorafenib could promote cell immunogenic cell death in NPC (Fig. 8A). The findings are as follows: NCG immunodeficient mouse xenograft models using 6-10B cells were established (Fig. 8B). The combined treatment group, consisting of sorafenib and T-cell co-cultivation, led to a significant decrease in tumor weight compared to control groups (Fig. 8C). Histological examination of tumor tissues revealed increased necrosis in the combined treatment group (Fig. 8D). Immunohistochemical staining for MHC-I showed enhanced MHC-I, GR and TAP1 expression, but not SLC7A11 expression, in the sorafenib treatment alone group and the combined treatment group (Fig. 8D). These results suggest that sorafenib treatment, in combination with T-cell co-cultivation, promotes immunogenic cell death in NPC tumors, indicating its potential as a therapeutic strategy for NPC. The BALB/C (nu/nu) nude mice have a congenital thymic defect that prevents T lymphocyte production, making them an ideal animal model for T-cell immunodeficiency [39]. This allows us to control the injection volume of exogenous human T-cells to nude mice. We established the subcutaneous tumor formation models using SUNE1 cells (Fig. 8E). The results indicated a significant decrease in tumor weight in the sorafenib combined with T-cell injection compared to control groups (Fig. 8F).

## Discussion

The mechanisms underlying tumor immune escape encompass loss of immunogenicity, an immunosuppressive microenvironment, and decreased antigenicity [40]. Impaired antigen presentation contributes to reduced tumor antigenicity [41]. Antigen peptides form a complex with MHC class I molecules, which then translocate to the cell membrane, activating immune cells. MHC-I levels are indicative of cell antigenicity, and aberrations in MHC-I correlate with unfavorable survival rates in NPC patients [8]. Investigating MHC-I expression regulation in NPC cells is crucial.

Dysregulated EGFR signaling propels tumor development. The overexpressed EGFR correlates with poor prognoses of NPC [9]. EGFR has been identified as a negative regulator of MHC-I expression and antigen presentation in various cancers [42]. Anti-EGFR therapy in head and neck squamous cell carcinoma can initiate adaptive immune response by promoting NK cells mediated significantly higher HLA-DR expression on DC to present tumor antigens to CD8^+^T cells [43]. However, studies on EGFR-mediated T cell recruitment in NPC are sparse. Our findings suggest a negative association between elevated EGFR expression and patient prognosis. At the same time, the expression level of EGFR is negatively correlated with the infiltration of CD8^+^T cells in tumor tissue and TAP1, which is a transporter to represent tumor antigen in the MHC-I complex. This suggests that the high expression of EGFR in NPC may be involved in the recruitment of CD8^+^T cells in NPC cells.

The epidermal growth factor receptor, a transmembrane entity, binds specific ligands via its extracellular domain. This binding induces dimerization and cytoplasmic domain autophosphorylation, initiating carcinogenic signaling cascades [44]. The main pathogenic process of EGFR in most tumors displayed EGFR tyrosine kinase domain activating mutations [45]. Not only the EGFR mutations are seldom observed in NPC, but also tyrosine kinase inhibitors don’t seem beneficial [9], it raises questions about the carcinogenicity of EGFR might be independent of its tyrosine kinase activity in NPC [46]. In further mass spectrometry analysis, we focused on the binding protein SLC7A11 to EGFR. It was found that EGFR can bind to SLC7A11 and stabilize SLC7A11 in a kinase independent manner. Previous studies have also supported that SLC7A11 binds to EGFR to inhibit the kinase activity of it, as the cytoplasmic domain of EGFR interacts with the central part of SLC7A11, inhibiting dimerization of the EGFR intracellular segment, inactivating EGFR kinase activity, and stabilizing SLC7A11 protein in a kinase independent manner [15]. We used the first-generation EGFR tyrosine kinase inhibitors (TKI) gefitinib, reversibly binds to the ATP-binding pocket, thereby inhibiting EGFR autophosphorylation. Second-generation EGFR inhibitors, including afatinib and dacomitinib, also inhibit EGFR but irreversibly bind to C797 through their side chains, preventing autophosphorylation. The T790M mutation reduces drug binding within the ATP pocket and enhances ATP affinity, leading to resistance to first- and second-generation TKIs. Third-generation TKIs, such as osimertinib, are irreversible kinase inhibitors targeting EGFR T790M and effectively inhibit EGFR autophosphorylation [47]. Although our study focused on gefitinib, we thought its short-term inhibitory effects on EGFR autophosphorylation to be comparable to those of other TKIs. As for the slight changes in SLC7A11 following EGF stimulation, we believe that it may be due to excessive stimulation intensity, which broke the binding between EGFR and SLC7A11. This needs to be further confirmed in future research.

SLC7A11 expression inversely correlates with CD8^+^T cell infiltration in various tumors, such as head and neck squamous cell carcinoma. Only patients with reduced SLC7A11 expression seem to benefit from immunotherapy [17]. Our research indicates that elevated SLC7A11 expression can shield NPC cells from T-cell-mediated cytotoxicity. When using RNA-seq analysis to knock down SLC7A11 in NPC cells, upregulation of downstream transcriptome levels can be enriched in TAP1 mediated antigen presentation. TAP1 is an important component of the stable peptide MHC-I complex. The absence of MHC-I on the cell membrane is a frequent observation in diverse tumors. The loss of any component of the MHC-I peptide complex can result in diminished MHC-I membrane expression, as seen with deletions of MHC-I heavy chain, β2m, immune protease subunits, TAP, Tapasin, and ERAP1. In both mouse and human cells, *TAP* gene deletion leads to a 30-70% reduction in MHC-I levels across most MHC-I alleles [48]. In colorectal cancer, cervical cancer, melanoma, and esophageal cancer, the loss of TAP1 expression ranges from 10% to 80.4%, and the loss of TAP1 expression suggests poor prognosis in tumor patients [49–52]. Our research corroborates that TAP1 promotes MHC-I membrane expression in NPC. Moreover, SLC7A11 modulates MHC-1 protein membrane levels by suppressing TAP1 expression. This phenomenon is attributed to SLC7A11 fostering heightened glucose dependence in tumors, which in turn prevents the transcription factor GR from nuclear entry, thereby downregulating TAP1 transcription. For the first time, the regulatory interplay between SLC7A11 and GR, as well as the transcription factor relationship between GR and TAP1, has been elucidated.

Persistent UPR activation typically leads to cell death pathways, eliminating potentially malignant cells. Invasive cancers often survive under chronic stress [53], with some tumors co-opting ERAD to maintain endoplasmic reticulum homeostasis [54]. VCP can directly or via adapters like UBXD7/UBX5 interact with E3 ubiquitin ligases to enhance ubiquitination of ERAD substrates [55, 56], further promoting the return translocation of misfolded proteins for proteasomal degradation. In HER2-positive breast cancers, there’s an upregulation of genes associated with the ERAD pathway [57]. Lin et al. observed elevated VCP levels in human breast cancer tissues, especially in the CSC population, when compared to non-CSC cells. This elevation also correlated with factors such as histological grade, tumor size, and lymph node metastasis. Notably, VCP-mediated protein regulation and ERAD play vital roles in breast cancer CSCs [58]. Michael Boutros determined that FAF2 acts as a ubiquitin adapter for VCP, facilitating the transfer of ubiquitinated substrates to proteasomes [59]. This suggests that FAF2 amplifies VCP-mediated ubiquitin-dependent ERAD pathways. We also analyzed the enrichment of downregulated genes post-SLC7A11 knockdown and discerned a potential link between the ERAD pathway enrichment and MHC-I protein expression in NPC. Normally, if MHC-I HCs don’t bind with β2m or acquire peptides, they misfold. This aberrant MHC-I heavy chain is identified by EDEM1, OS-9, and XTP3-B within the ER, relying on a glycan-dependent process, and is subsequently degraded via the ERAD pathway [60, 61]. A newly identified ER protein, SND1, redirects nascent MHC-I HC towards ERAD-proteasomal degradation, obstructing HC and β2m assembly in the ER. This aids tumor cells in evading CD8^+^T cell immunity [33]. Studies indicate that targeting the ERAD pathway, particularly VCP, offers a novel therapeutic avenue for cancers. Specific ERAD inhibitors, like Eeyarestatin I, NMS-97, and DBEQ, have been shown to hinder esophageal cancer cell activity and induce pancreatic cancer cell apoptosis [62, 63]. We observed that SLC7A11 downregulated MHC-I protein membrane expression, also dependent on FAF2. Silencing SLC7A11 enhanced MHC-I protein stability. Moreover, ERAD inhibitors like Eeyarestatin I and VCP inhibitors such as NMS-873 re-established ubiquitination levels, suggesting SLC7A11-driven MHC-I degradation via the ubiquitin-dependent ERAD pathway. This is the inaugural confirmation of MHC-1 membrane expression regulation through SLC7A11-mediated ERAD degradation, contingent upon FAF2. FAF2, also known as UBXD8, is dual-localized to the mitochondria and ER [64]. It forms complexes with mitochondrial and ER ubiquitin E3 ligases, recruiting VCP substrates to ER for degradation [65]. Additionally, FAF2 is an important component of mitochondria-associated degradation (MAD). It mediates MAD to inhibit apoptosis and mitophagy [66]. Research has revealed close crosstalk between MAD and ERAD. This collaboration may promote the degradation of subsets of mitochondrial and ER substrates that are localized to or diffusing to contact sites [66]. Our research found that upregulation of SLC7A11 promotes FAF2 expression, facilitating ERAD and inducing MHC-I degradation in NPC. We speculate that the possible mechanism of FAF2 expression depends on the antioxidant activity of SLC7A11. It is a cystine/glutamate antiporter that transports cystine into cells, a rate-limiting precursor of glutathione, one of the most abundant cellular antioxidants [67]. Therefore, high expression of SLC7A11 can limit reactive oxygen species (ROS) accumulation in NPC cells [24], reducing damage to the endoplasmic reticulum [68] and mitochondria [69]. which promotes the expression of FAF2, localized to the ER and mitochondria.

Building upon prior studies highlighting SLC7A11’s oncogenic role in NPC, we evaluated the therapeutic potential of its functional inhibitor, sorafenib. Earlier constrained clinical trials showcased sorafenib’s tolerability and its moderate anticancer efficacy in treating NPC, either as a monotherapy [70] or in combination with other agents [71]. Aiming to further understand how targeting the EGFR-binding protein SLC7A11 influences MHC-I antigen presentation and induces T-cell mediated cytotoxicity in NPC cells, we sought to elucidate the therapeutic mechanisms of sorafenib in NPC. Recognizing sorafenib acts as a multi-kinase inhibitor and to rule out off-target effects, we examined proteomic and phosphoproteomic alterations in sorafenib-treated NPC cells. Our findings revealed heightened antigen presentation and MHC-I complex peptide enrichment, with no discernible increase in other pathways like Raf and VEGFR. Post co-culture with T cells, sorafenib treatment was observed to bolster T-cell IFN-γ release, triggering cytotoxic effects on NPC cells and fostering tumor cell apoptosis. Concurrently, in vivo studies demonstrated the potential of sorafenib in promoting NPC cells death. Immunohistochemical staining showed enhanced expression of TAP1, GR, and MHC-I, while SLC7A11 expression remained unchanged. This change is related to the mechanism of sorafenib. Sorafenib inhibits SLC7A11 transporter activity by blocking SLC7A11-mediated cystine uptake, rather than regulating SLC7A11 expression [72]. In the future, we will further investigate the value and mechanism of sorafenib in the treatment of NPC.

**Fig. 9:**
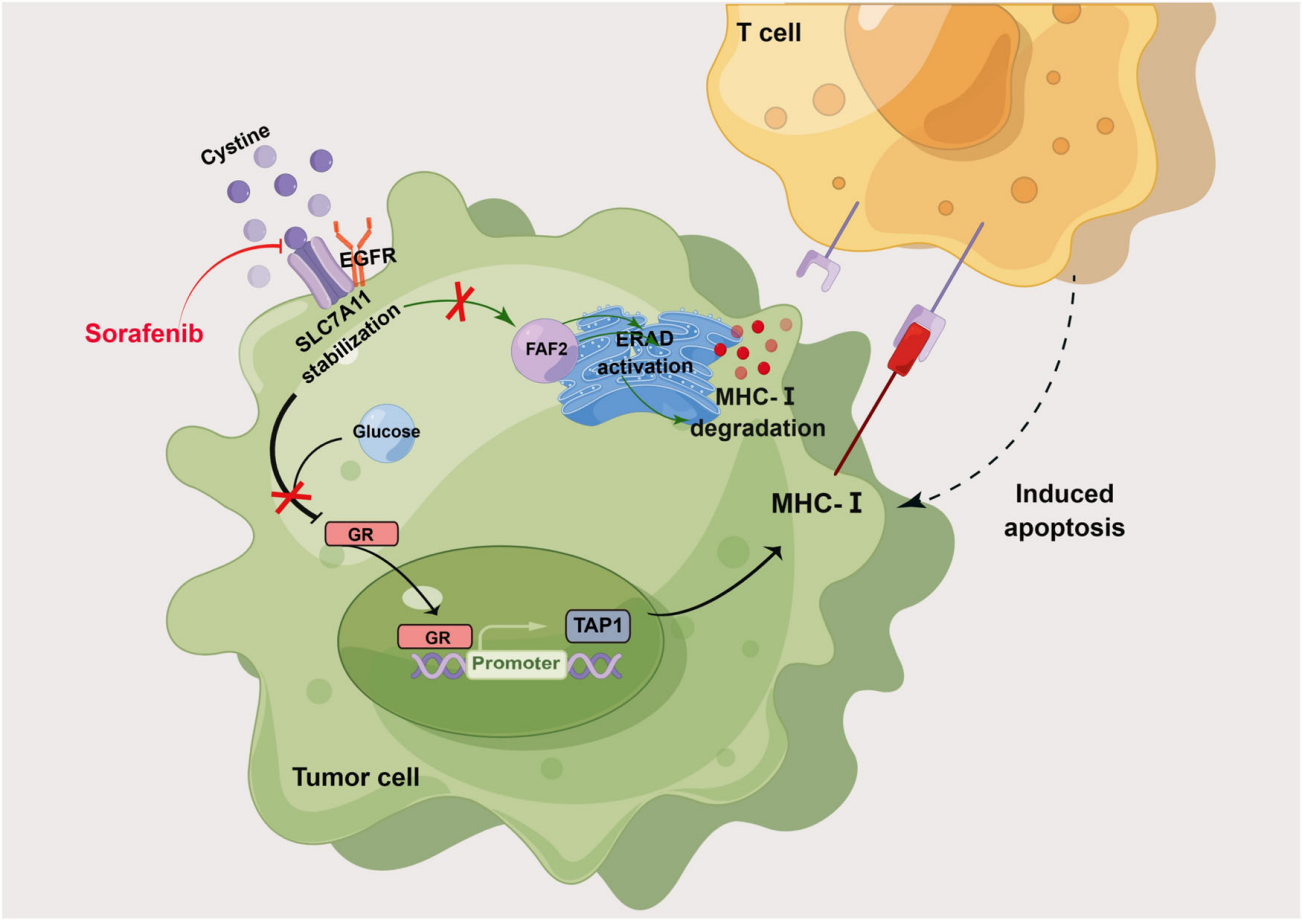
Mechanism of Targeting EGFR-binding protein SLC7A11 enhancing antitumor immunity of T cells via inducing MHC-I antigen presentation in nasopharyngeal carcinoma. EGFR stabilizes SLC7A11 expression. The high and stable SLC7A11 expression not only limits GR nuclear entry, inhibiting TAP1 transcription and MHC-I presentation, but also enhances FAF2 expression and activates ERAD-dependent MHC-I degradation, leading to reduced MHC-I on the cell membrane. Furthermore, the targeting of SLC7A11 by sorafenib is considered a potential therapeutic strategy for NPC treatment. Schematic Fig. was drawn by Figdraw (www.figdraw.com).

In closing, we revealed several key findings related to the role of SLC7A11 in NPC (Fig. 9). SLC7A11, which is stabilized by EGFR, was found to protect NPC cells from T cell-induced cytotoxicity. High SLC7A11 levels in NPC cells led to reduced MHC-I antigen presentation by limiting TAP1 transcription and activating ERAD-dependent MHC-I degradation, which can hinder the recognition of cancer cells by the immune system. Importantly, the study identified the targeting of SLC7A11 by sorafenib as a potential therapeutic strategy for NPC treatment. This research provides new insights into the molecular mechanisms underlying NPC progression and highlights the potential of sorafenib to enhance NPC cell antigen presentation and stimulate T-cell immune responses. These findings have implications for the development of novel treatment approaches for NPC and shed light on the role of SLC7A11 in the context of this cancer.

## Supplementary information

The raw data of WB

EGFR stabilizes SLC7A11 protein expression in NPC via a kinase-independent mechanism

Histogram of predicted immune cell infiltrate levels in the GSE53819 dataset which with higher SLC7A11 levels in NPC tissues

Glucose-dependent properties of high SLC7A11 expression inhibit TAP1 transcription through GR, weakening MHC-I membrane expression in NPC cells

SLC7A11 enhances FAF2 expression, activating the ERAD pathway implicated in the ubiquitin-mediated degradation of MHC-I

The phosphorylated protein changes post-sorafenib

## Data Availability

The datasets generated during and/or analyzed during the current study are available from the corresponding author upon reasonable request. PRO-Seq data were aquired from the Gene Expression Omnibus database under accession number GSE53819 and are available at the following URL: https://www.ncbi.nlm.nih.gov/geo/query/acc.cgi?acc=GSE53819.
