# Supplementary material for: Targeting EGFR-binding protein SLC7A11 enhancing antitumor immunity of T cells via inducing MHC-I antigen presentation in nasopharyngeal carcinoma
Source: Cell Death Dis. 2025 Jan 16;16(1):21. doi: 10.1038/s41419-024-07327-9 (PMC11739652; doi:10.1038/s41419-024-07327-9)
Supplement: Supplementary file 1 — The raw data of WB [file 41419_2024_7327_MOESM1_ESM.pptx]

## Slide 1
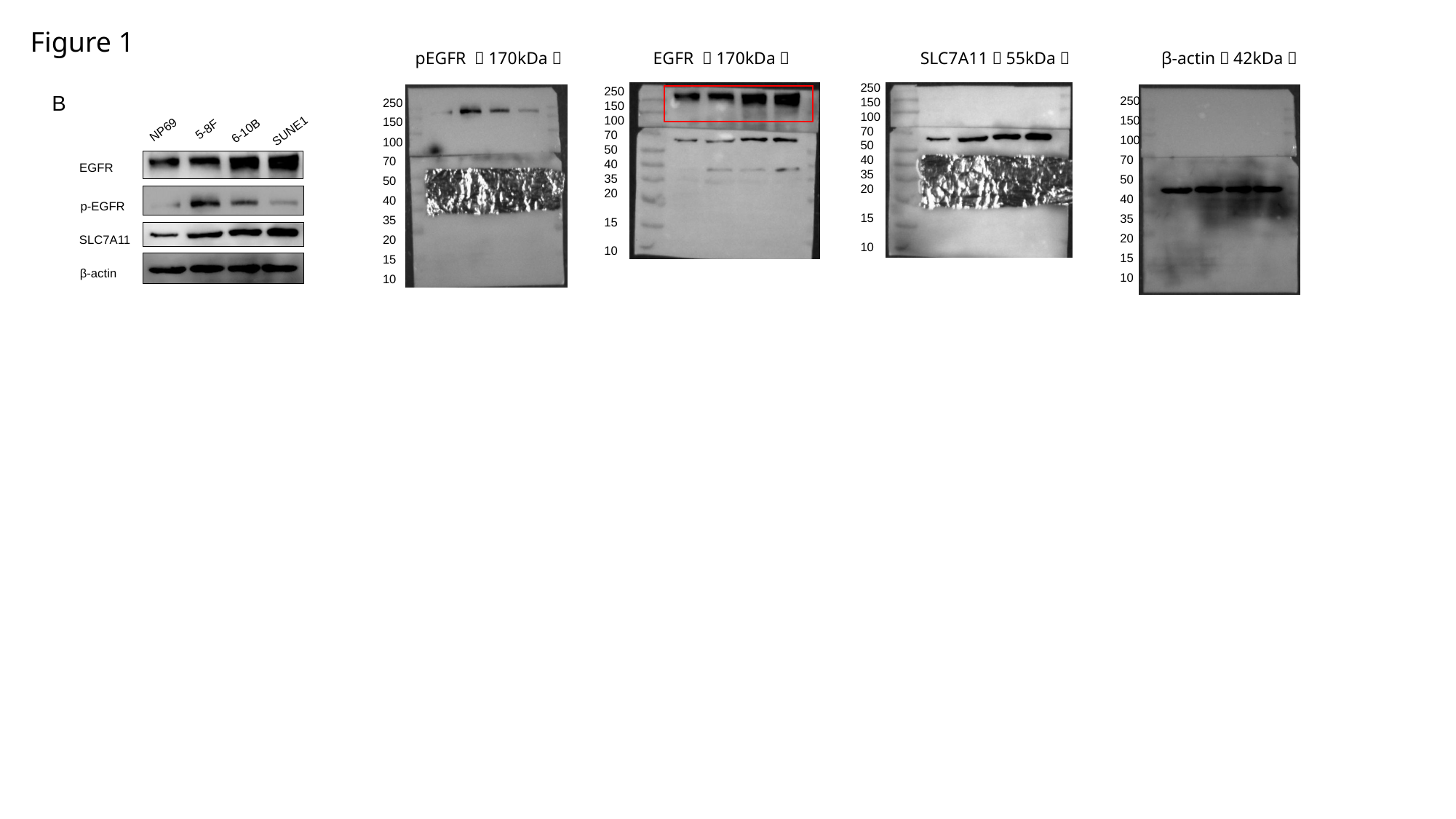

Figure 1
pEGFR （170kDa） EGFR （170kDa） SLC7A11（55kDa） β-actin（42kDa）
250
150
100
70
50
40
35
20
15
10
250
150
100
70
50
40
35
20
15
10
250
150
100
70
50
40
35
20
15
10
250
150
100
70
50
40
35
20
15
10
B
6-10B
NP69
SUNE1
5-8F
EGFR
SLC7A11
β-actin
p-EGFR

## Slide 2
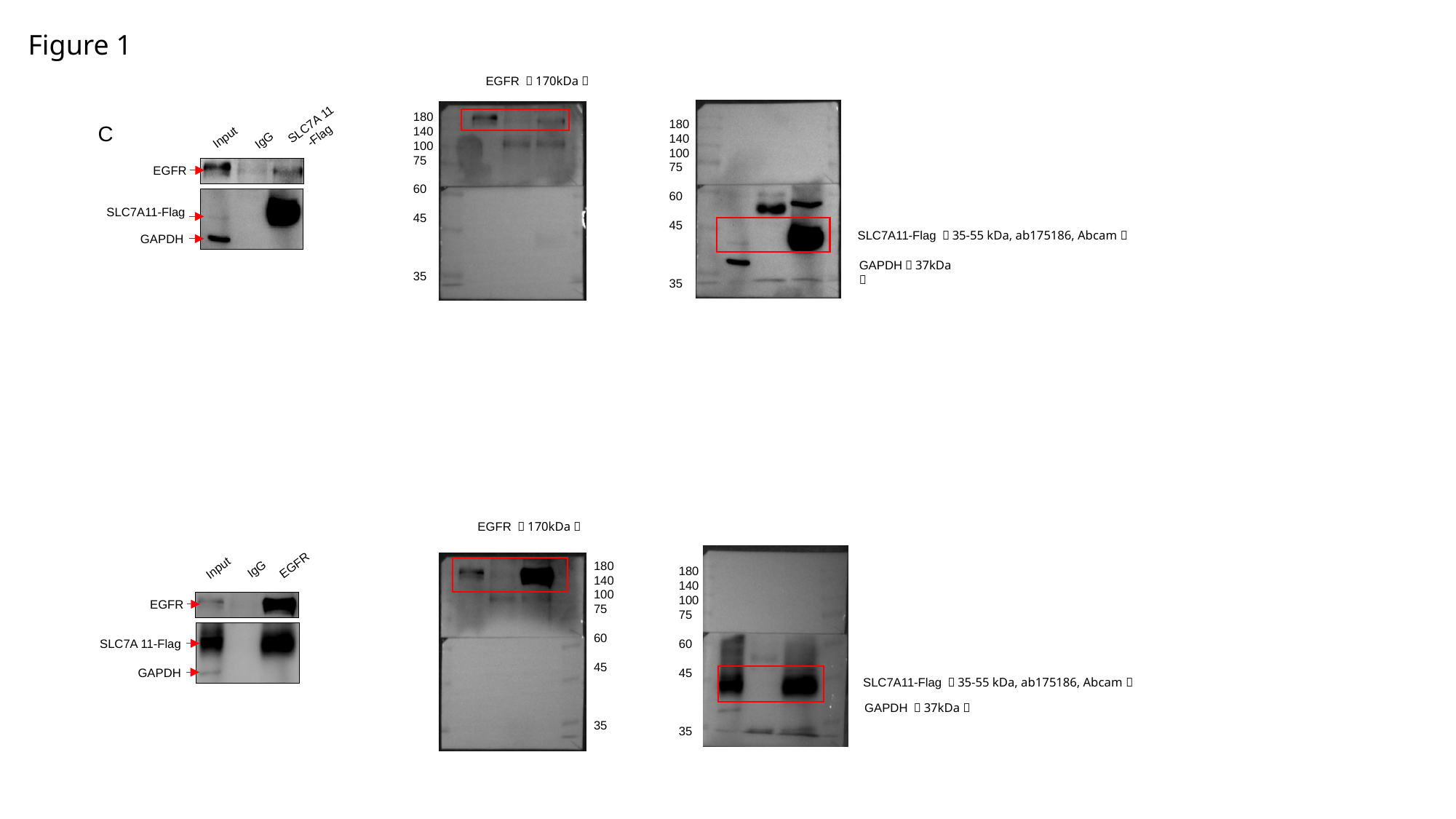

Figure 1
EGFR （170kDa）
180
140
100
75
60
45
35
SLC7A 11
-Flag
Input
IgG
EGFR
SLC7A11-Flag
GAPDH
180
140
100
75
60
45
35
C
SLC7A11-Flag （35-55 kDa, ab175186, Abcam）
GAPDH（37kDa）
EGFR （170kDa）
180
140
100
75
60
45
35
SLC7A11-Flag （35-55 kDa, ab175186, Abcam）
GAPDH （37kDa）
EGFR
Input
IgG
EGFR
SLC7A 11-Flag
GAPDH
180
140
100
75
60
45
35

## Slide 3
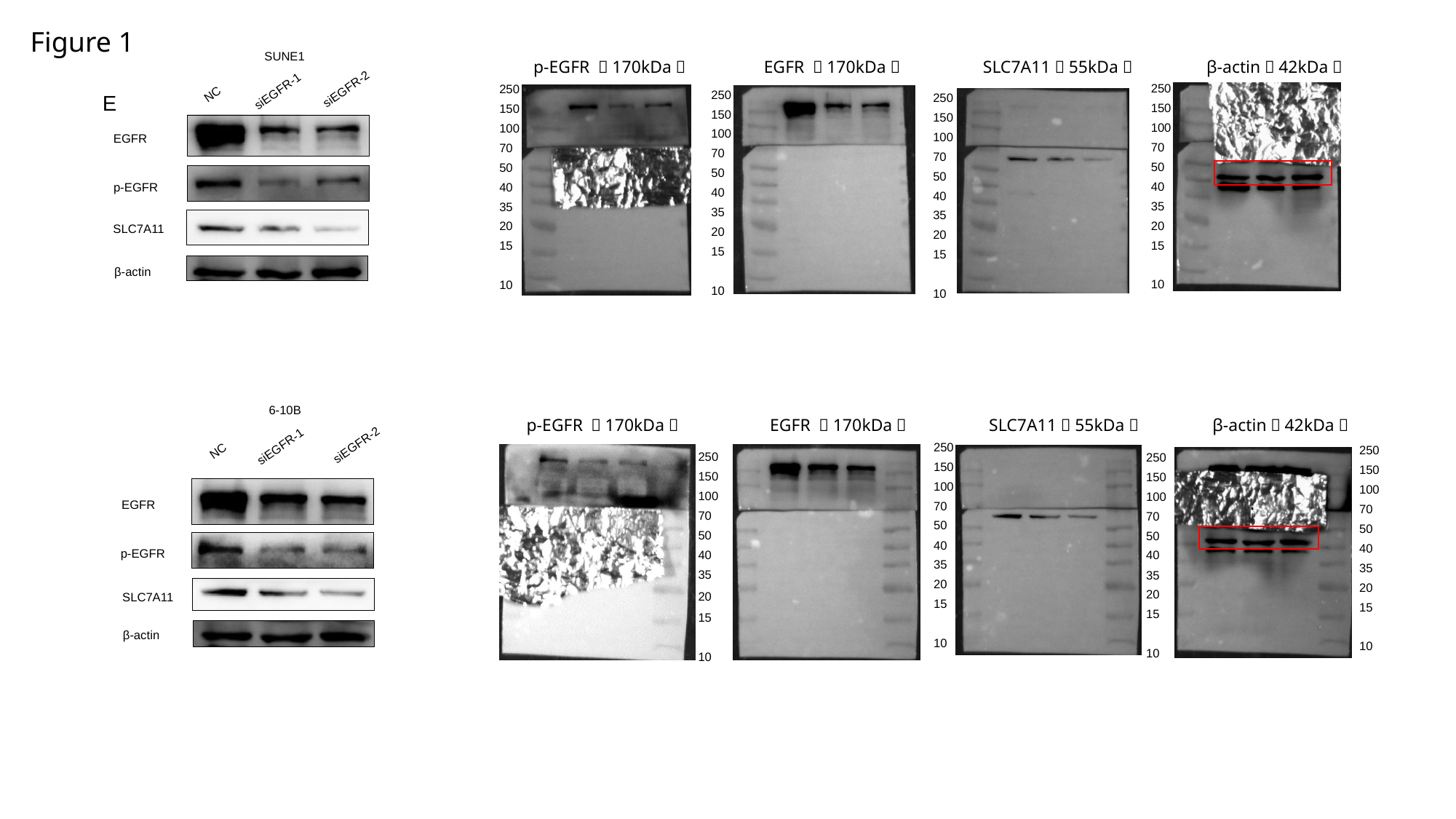

Figure 1
SUNE1
siEGFR-2
siEGFR-1
NC
EGFR
SLC7A11
β-actin
p-EGFR
p-EGFR （170kDa） EGFR （170kDa） SLC7A11（55kDa） β-actin（42kDa）
250
150
100
70
50
40
35
20
15
10
250
150
100
70
50
40
35
20
15
10
250
150
100
70
50
40
35
20
15
10
250
150
100
70
50
40
35
20
15
10
E
6-10B
siEGFR-2
siEGFR-1
NC
EGFR
SLC7A11
β-actin
p-EGFR
p-EGFR （170kDa） EGFR （170kDa） SLC7A11（55kDa） β-actin（42kDa）
250
150
100
70
50
40
35
20
15
10
250
150
100
70
50
40
35
20
15
10
250
150
100
70
50
40
35
20
15
10
250
150
100
70
50
40
35
20
15
10

## Slide 4
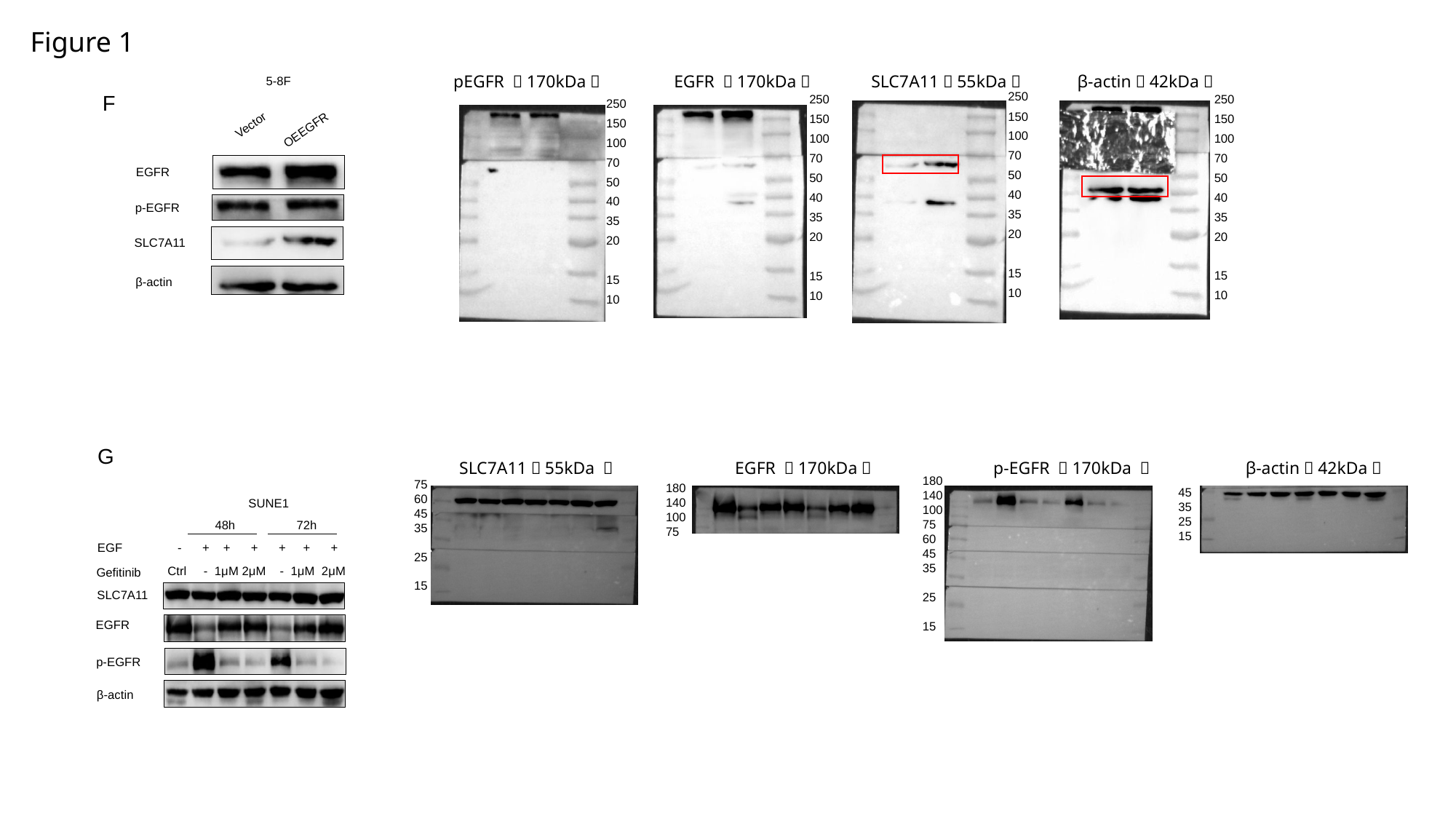

Figure 1
pEGFR （170kDa） EGFR （170kDa） SLC7A11（55kDa） β-actin（42kDa）
5-8F
250
150
100
70
50
40
35
20
15
10
Vector
OEEGFR
EGFR
p-EGFR
SLC7A11
β-actin
250
150
100
70
50
40
35
20
15
10
250
150
100
70
50
40
35
20
15
10
F
250
150
100
70
50
40
35
20
15
10
G
SLC7A11（55kDa ） EGFR （170kDa） p-EGFR （170kDa ） β-actin（42kDa）
180
140
100
75
60
45
35
25
15
75
60
45
35
25
15
180
140
100
75
45
35
25
15
SUNE1
48h
72h
EGF
Gefitinib
SLC7A11
EGFR
p-EGFR
β-actin
 - + + + + + +
Ctrl - 1μM 2μM - 1μM 2μM

## Slide 5
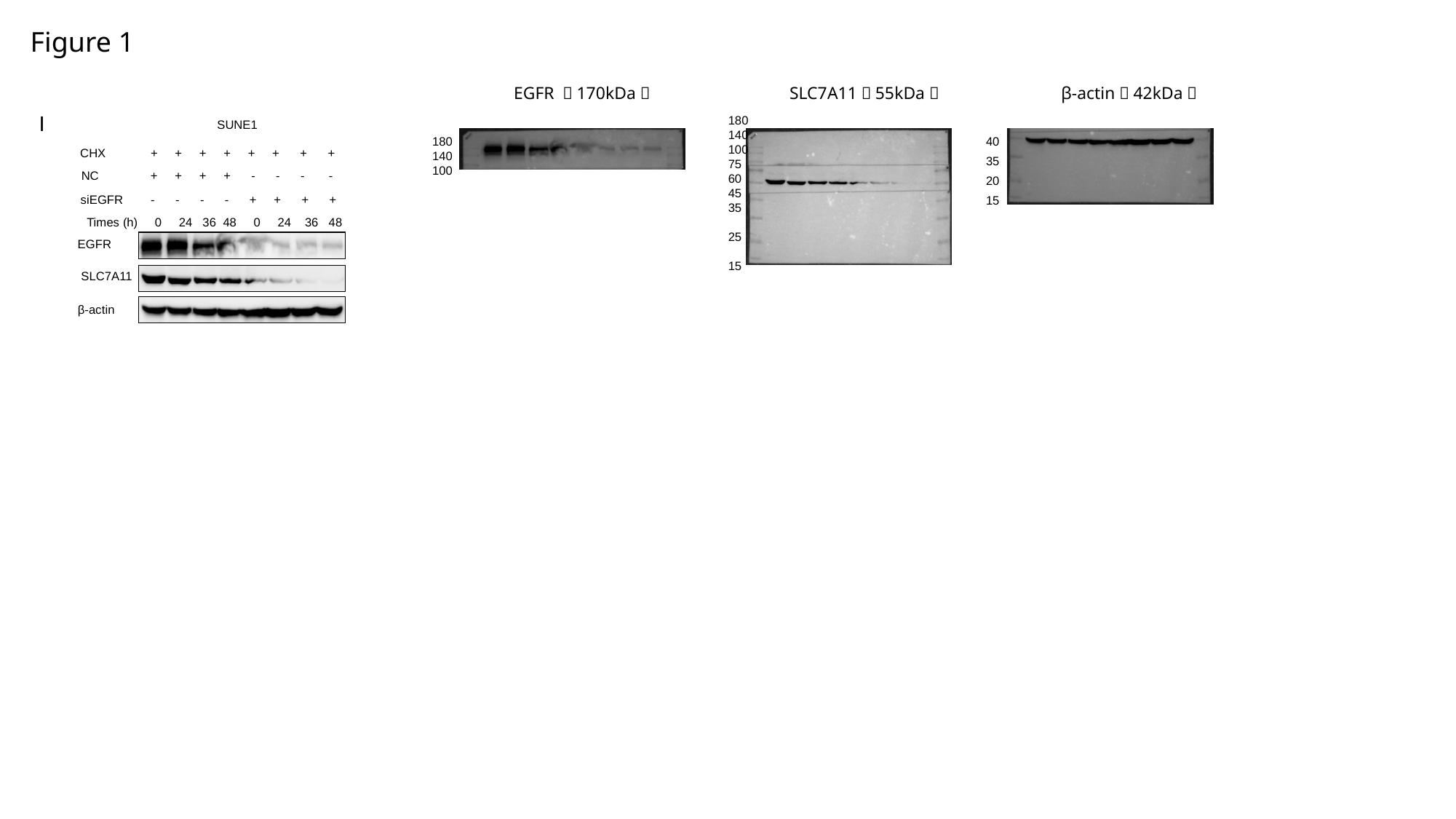

Figure 1
EGFR （170kDa） SLC7A11（55kDa） β-actin（42kDa）
I
180
140
100
75
60
45
35
25
15
SUNE1
siEGFR - - - - + + + +
EGFR
SLC7A11
β-actin
CHX + + + + + + + +
NC + + + + - - - -
Times (h) 0 24 36 48 0 24 36 48
40
35
20
15
180
140
100

## Slide 6
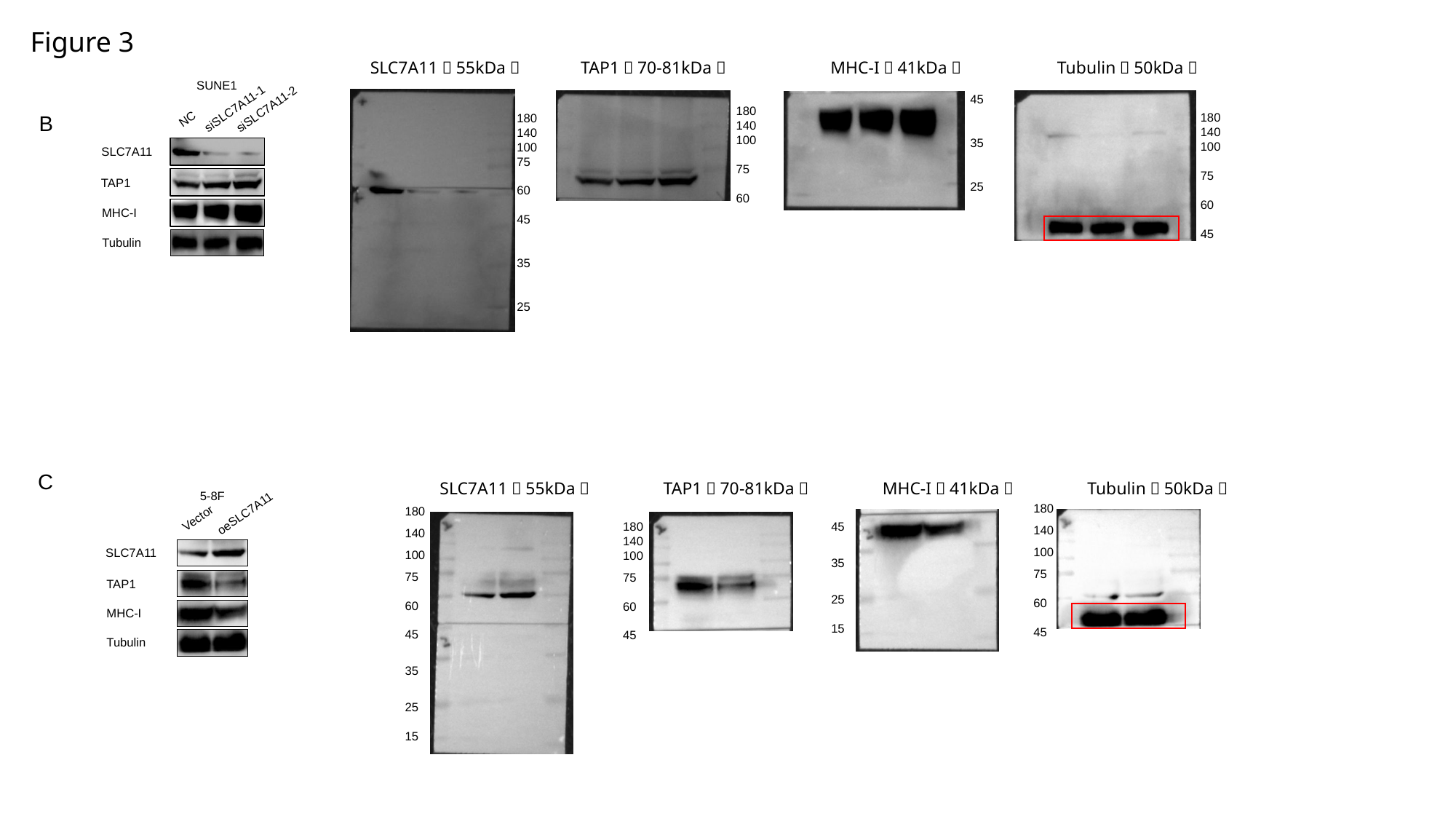

Figure 3
SLC7A11（55kDa） TAP1（70-81kDa） MHC-I（41kDa） Tubulin（50kDa）
45
35
25
SUNE1
siSLC7A11-1
siSLC7A11-2
NC
SLC7A11
TAP1
MHC-I
Tubulin
180
140
100
75
60
180
140
100
75
60
45
180
140
100
75
60
45
35
25
B
C
SLC7A11（55kDa） TAP1（70-81kDa） MHC-I（41kDa） Tubulin（50kDa）
5-8F
oeSLC7A11
Vector
SLC7A11
TAP1
Tubulin
MHC-I
180
140
100
75
60
45
180
140
100
75
60
45
35
25
15
45
35
25
15
180
140
100
75
60
45

## Slide 7
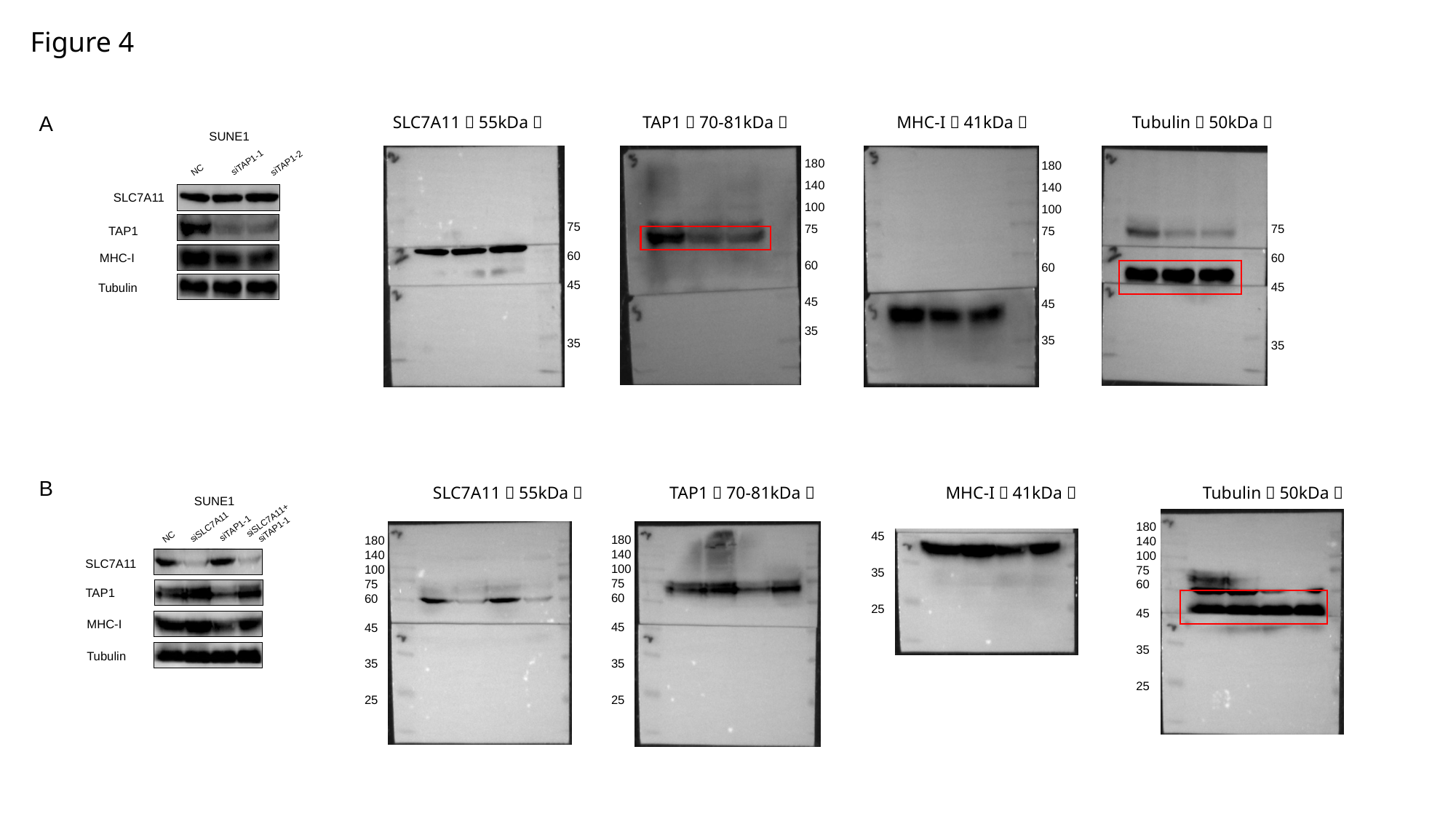

Figure 4
A
SLC7A11（55kDa） TAP1（70-81kDa） MHC-I（41kDa） Tubulin（50kDa）
SUNE1
siTAP1-1
siTAP1-2
NC
TAP1
MHC-I
Tubulin
SLC7A11
180
140
100
75
60
45
35
180
140
100
75
60
45
35
75
60
45
35
75
60
45
35
B
SLC7A11（55kDa） TAP1（70-81kDa） MHC-I（41kDa） Tubulin（50kDa）
SUNE1
siSLC7A11+
siTAP1-1
siTAP1-1
siSLC7A11
NC
TAP1
MHC-I
Tubulin
SLC7A11
45
35
25
180
140
100
75
60
45
35
25
180
140
100
75
60
45
35
25
180
140
100
75
60
45
35
25

## Slide 8
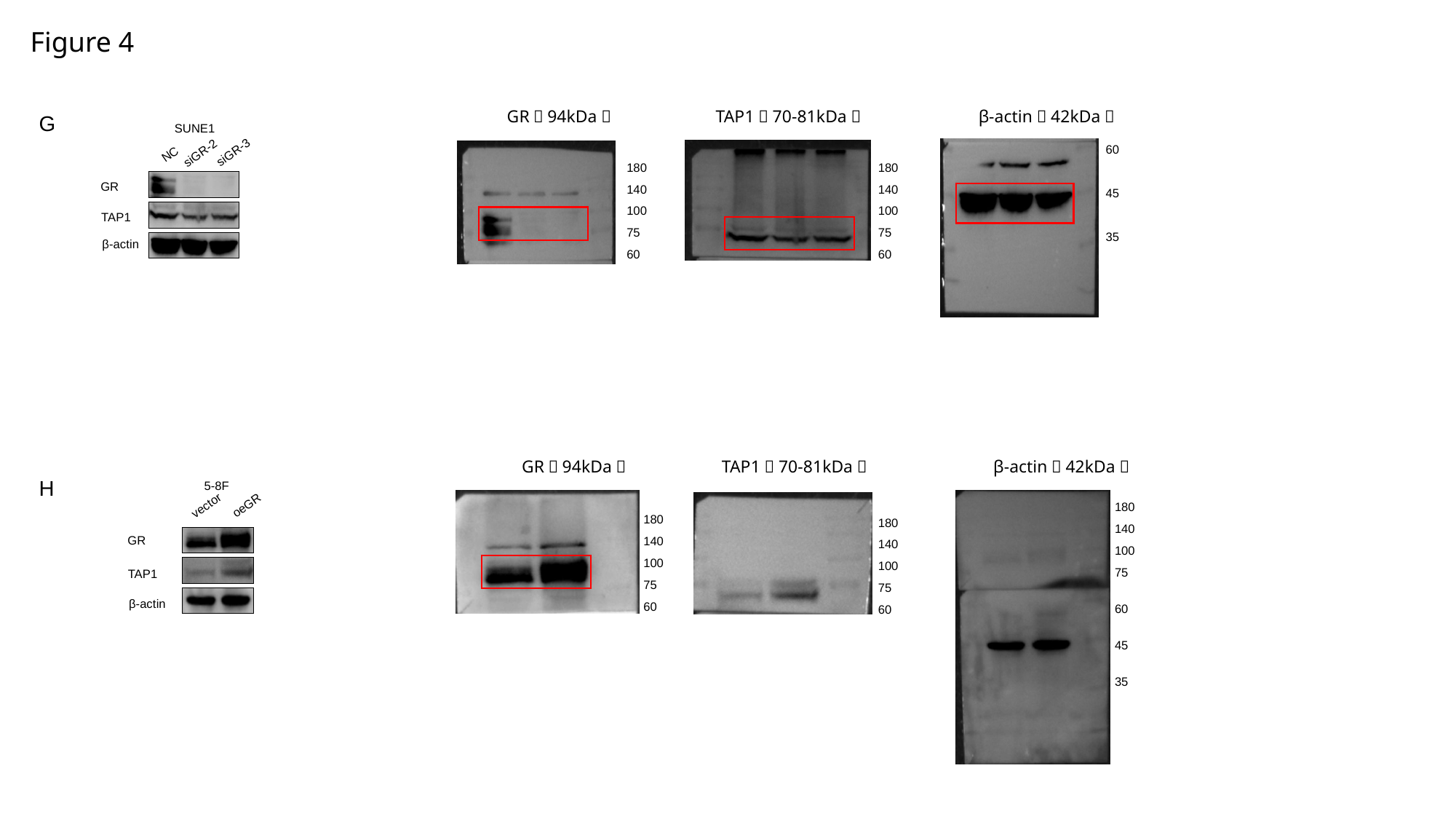

Figure 4
GR（94kDa） TAP1（70-81kDa） β-actin（42kDa）
G
60
45
35
SUNE1
siGR-3
siGR-2
NC
GR
TAP1
β-actin
180
140
100
75
60
180
140
100
75
60
GR（94kDa） TAP1（70-81kDa） β-actin（42kDa）
H
5-8F
vector
oeGR
GR
TAP1
β-actin
180
140
100
75
60
45
35
180
140
100
75
60
180
140
100
75
60

## Slide 9
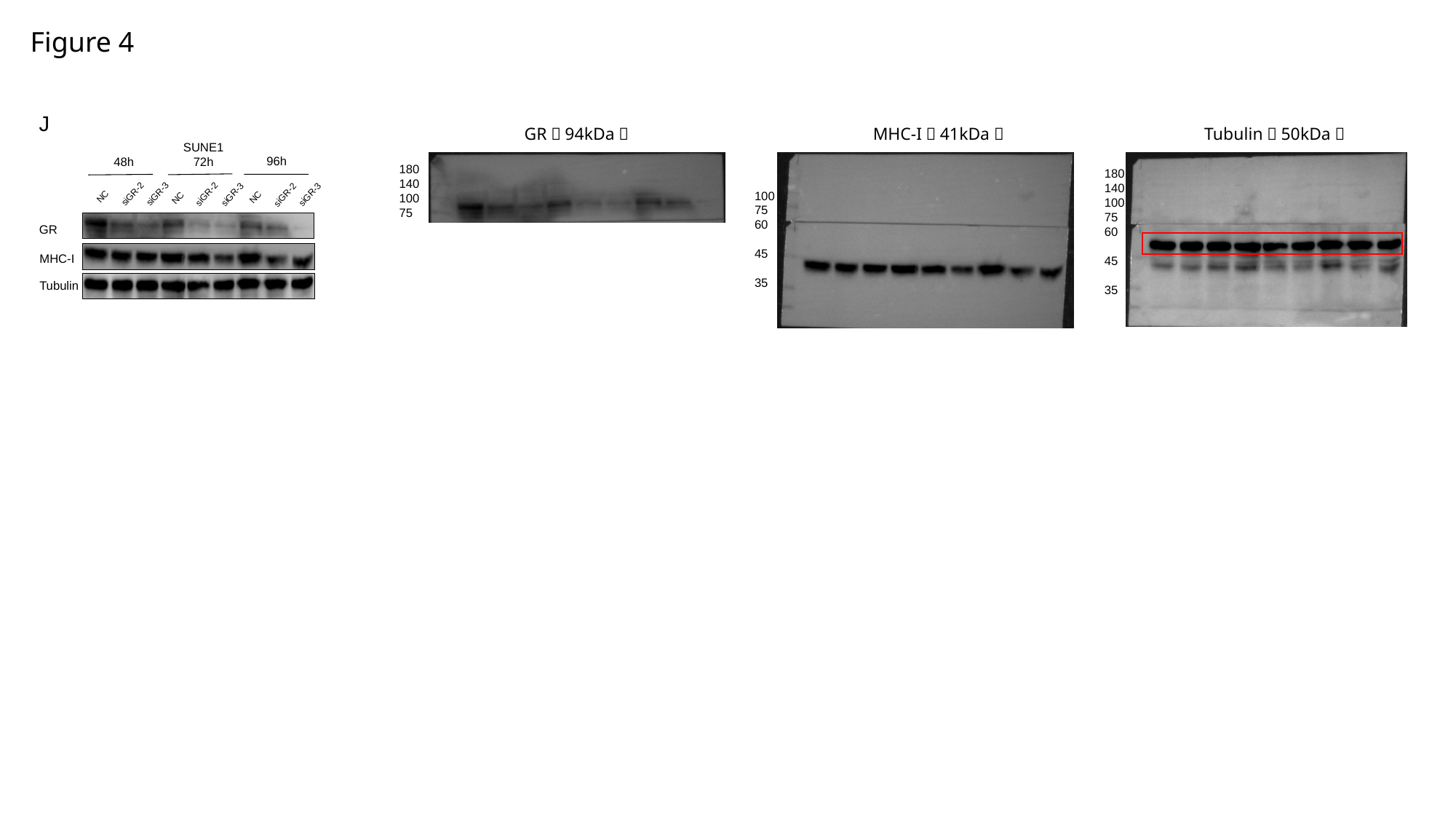

Figure 4
J
GR（94kDa） MHC-I（41kDa） Tubulin（50kDa）
SUNE1
96h
48h
72h
siGR-3
siGR-2
siGR-2
siGR-3
siGR-3
siGR-2
NC
NC
NC
GR
MHC-I
Tubulin
180
140
100
75
180
140
100
75
60
45
35
100
75
60
45
35

## Slide 10
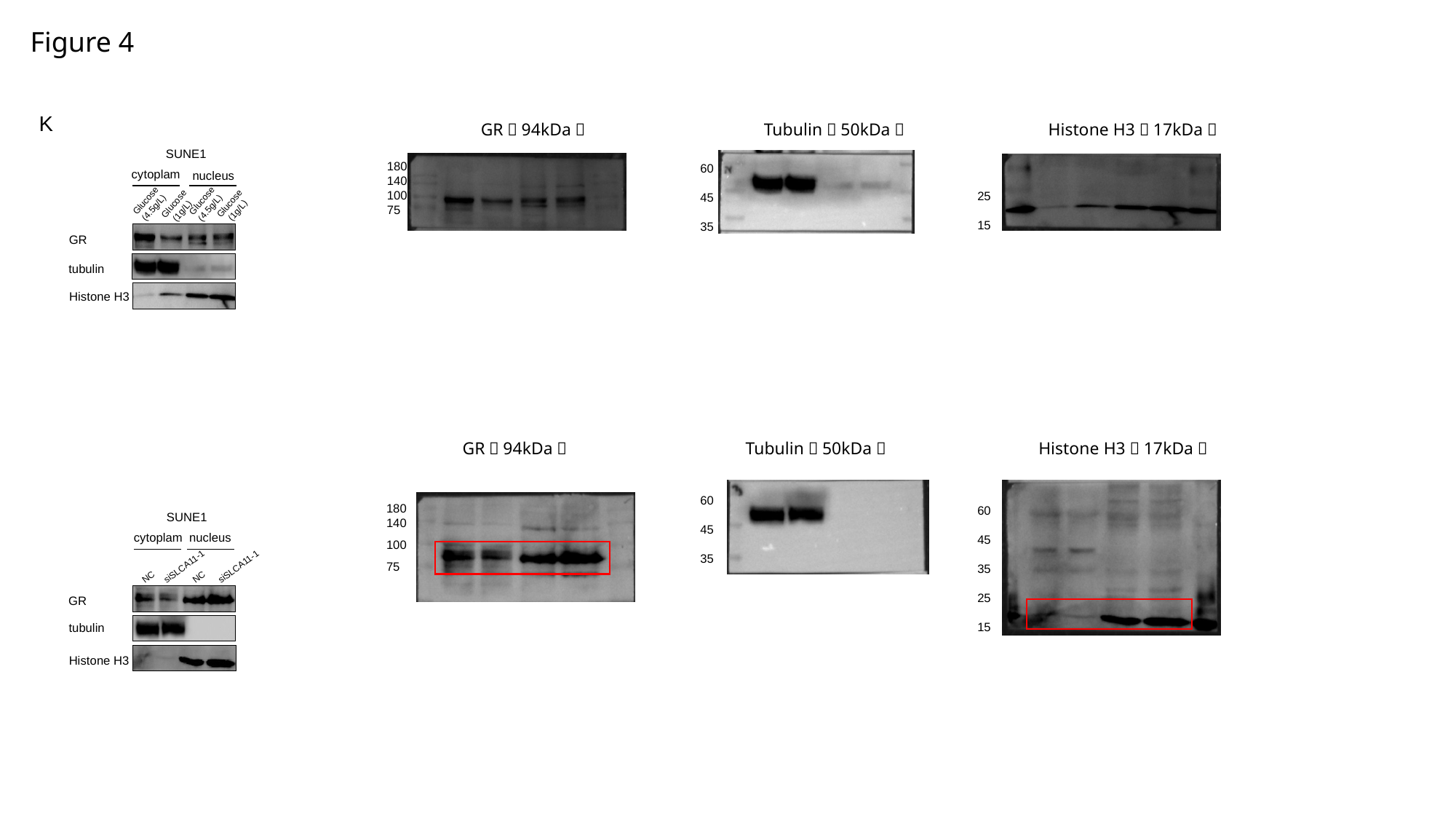

Figure 4
K
GR（94kDa） Tubulin（50kDa） Histone H3（17kDa）
60
45
35
SUNE1
cytoplam
nucleus
Glucose
(4.5g/L)
Glucose
(4.5g/L)
Glucose
(1g/L)
Glucose
(1g/L)
GR
tubulin
Histone H3
180
140
100
75
25
15
GR（94kDa） Tubulin（50kDa） Histone H3（17kDa）
60
45
35
60
45
35
25
15
180
140
100
75
SUNE1
cytoplam
nucleus
siSLCA11-1
siSLCA11-1
NC
NC
GR
tubulin
Histone H3

## Slide 11
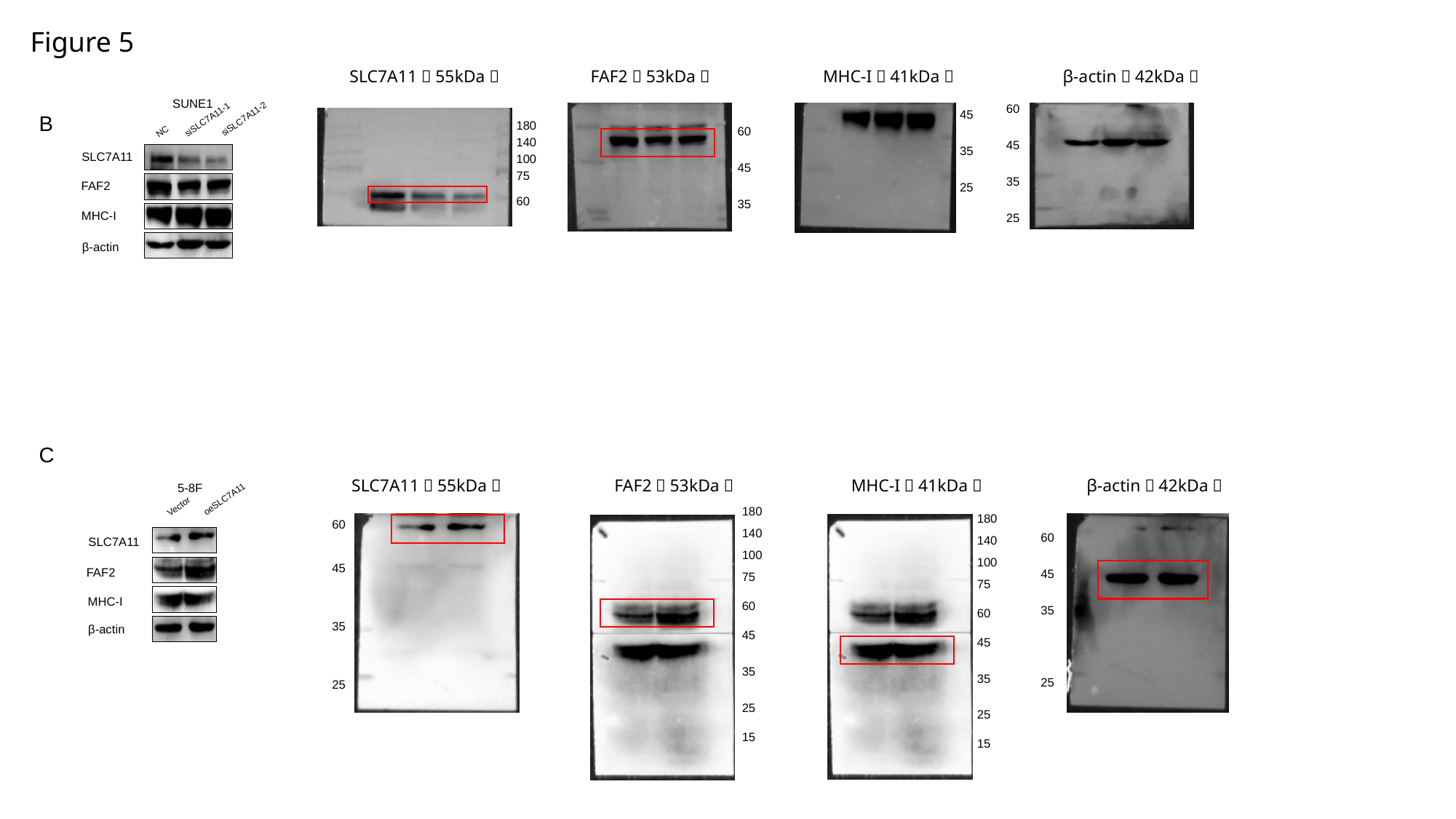

Figure 5
SLC7A11（55kDa） FAF2（53kDa） MHC-I（41kDa） β-actin（42kDa）
60
45
35
25
45
35
25
SUNE1
siSLC7A11-2
siSLC7A11-1
NC
SLC7A11
FAF2
MHC-I
β-actin
60
45
35
B
C
180
140
100
75
60
SLC7A11（55kDa） FAF2（53kDa） MHC-I（41kDa） β-actin（42kDa）
5-8F
oeSLC7A11
Vector
SLC7A11
FAF2
β-actin
MHC-I
60
45
35
25
180
140
100
75
60
45
35
25
15
180
140
100
75
60
45
35
25
15
60
45
35
25

## Slide 12
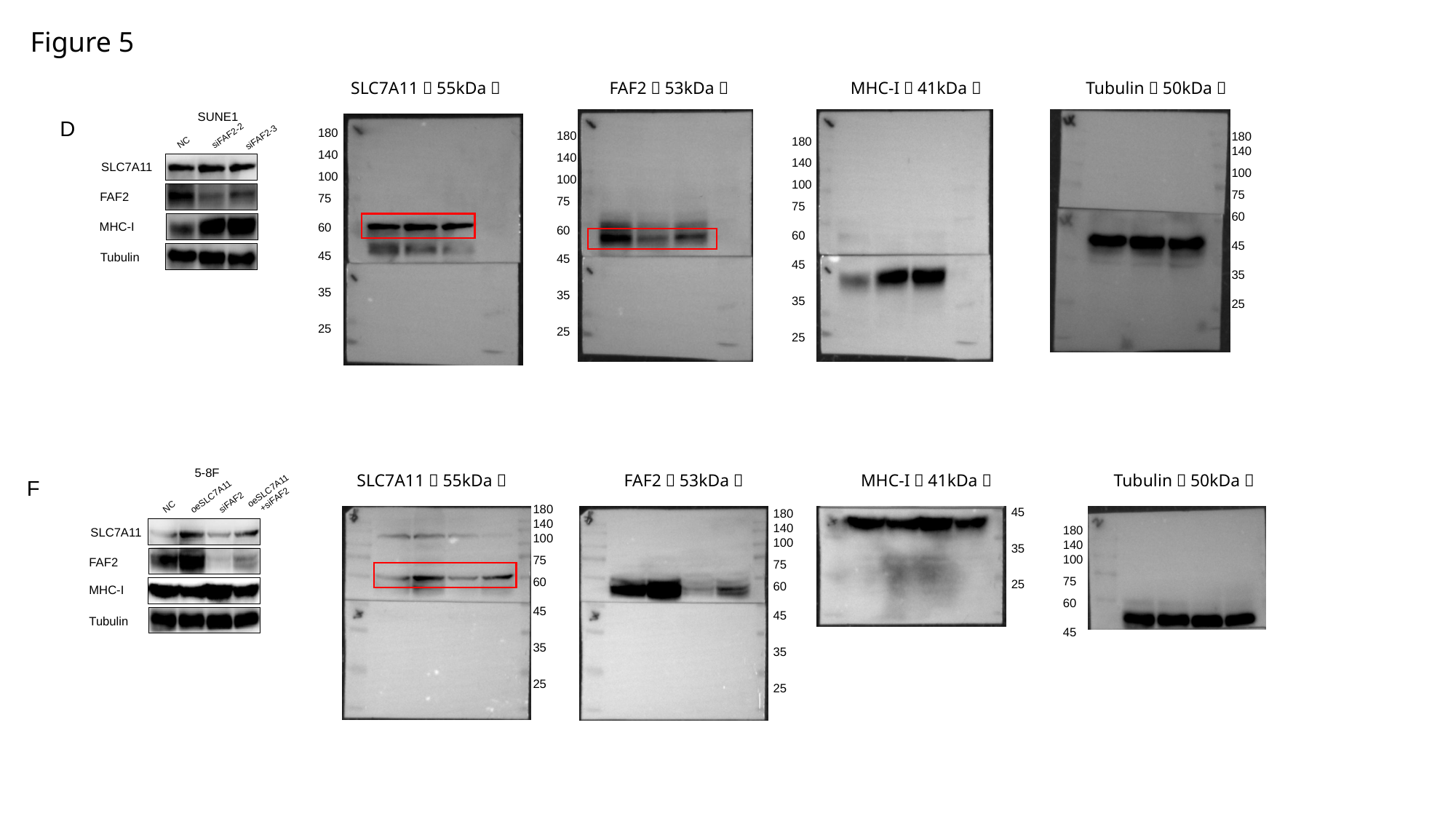

Figure 5
SLC7A11（55kDa） FAF2（53kDa） MHC-I（41kDa） Tubulin（50kDa）
SUNE1
FAF2
MHC-I
Tubulin
siFAF2-2
siFAF2-3
NC
SLC7A11
D
180
140
100
75
60
45
35
25
180
140
100
75
60
45
35
25
180
140
100
75
60
45
35
25
180
140
100
75
60
45
35
25
5-8F
oeSLC7A11
+siFAF2
siFAF2
oeSLC7A11
NC
FAF2
MHC-I
Tubulin
SLC7A11
F
45
35
25
180
140
100
75
60
45
35
25
180
140
100
75
60
45
35
25
180
140
100
75
60
45
SLC7A11（55kDa） FAF2（53kDa） MHC-I（41kDa） Tubulin（50kDa）

## Slide 13
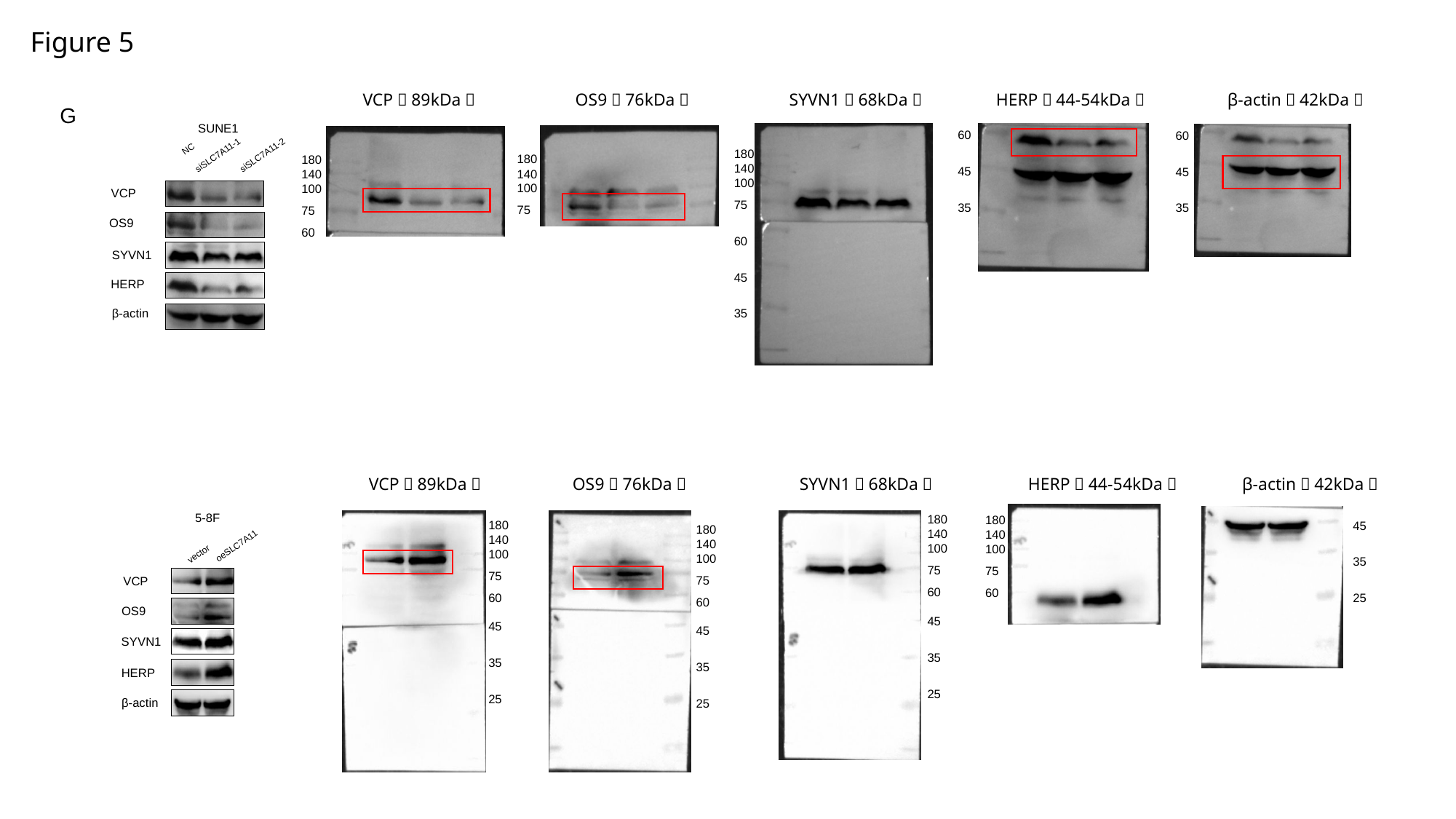

Figure 5
VCP（89kDa） OS9（76kDa） SYVN1（68kDa） HERP（44-54kDa） β-actin（42kDa）
G
60
45
35
60
45
35
SUNE1
NC
siSLC7A11-2
siSLC7A11-1
VCP
OS9
SYVN1
HERP
β-actin
180
140
100
75
60
45
35
180
140
100
75
180
140
100
75
60
VCP（89kDa） OS9（76kDa） SYVN1（68kDa） HERP（44-54kDa） β-actin（42kDa）
45
35
25
5-8F
oeSLC7A11
vector
VCP
OS9
SYVN1
HERP
β-actin
180
140
100
75
60
45
35
25
180
140
100
75
60
180
140
100
75
60
45
35
25
180
140
100
75
60
45
35
25

## Slide 14
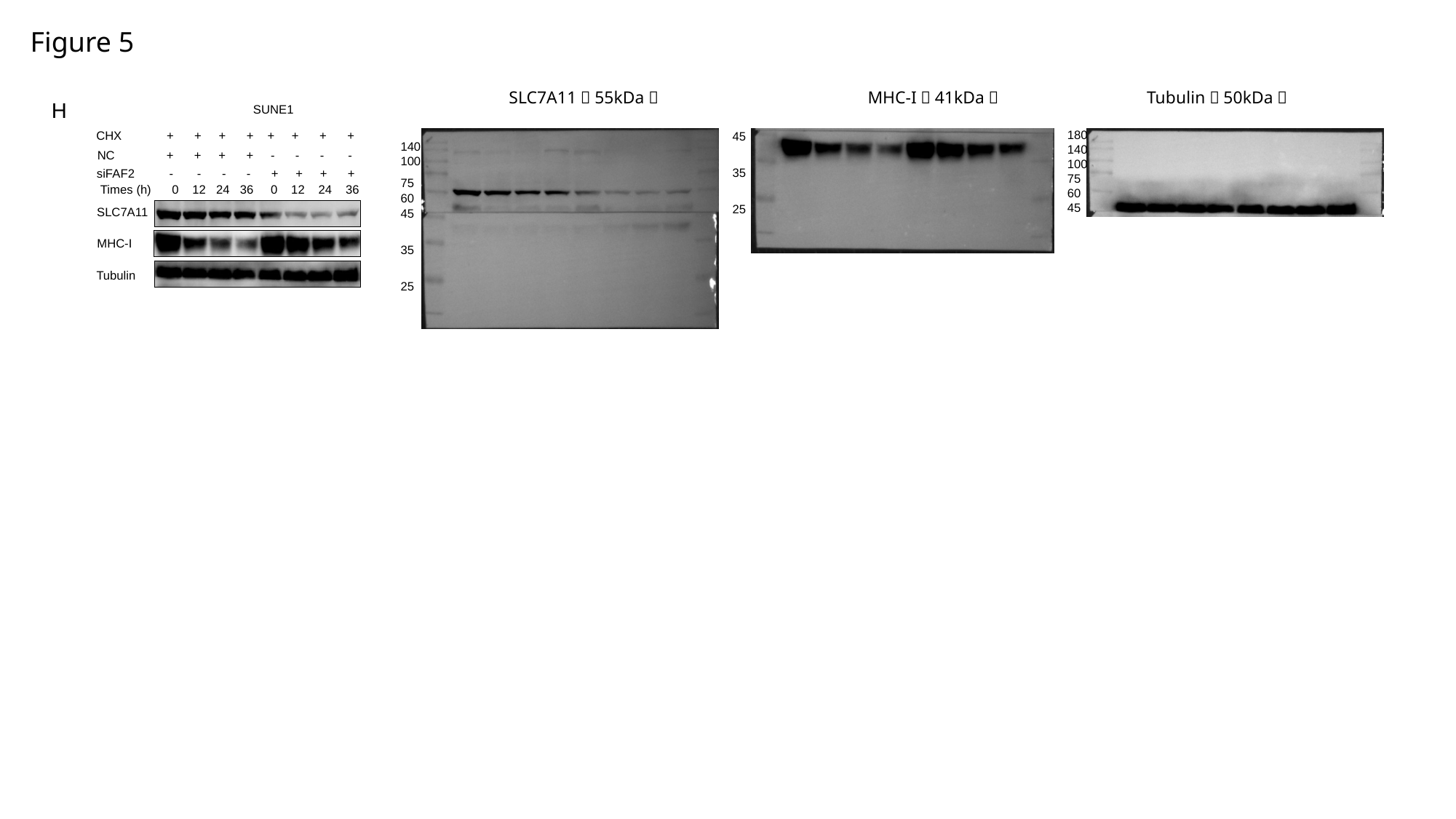

Figure 5
SLC7A11（55kDa） MHC-I（41kDa） Tubulin（50kDa）
H
SUNE1
siFAF2 - - - - + + + +
SLC7A11
MHC-I
Tubulin
CHX + + + + + + + +
 NC + + + + - - - -
Times (h) 0 12 24 36 0 12 24 36
45
35
25
180
140
100
75
60
45
140
100
75
60
45
35
25

## Slide 15
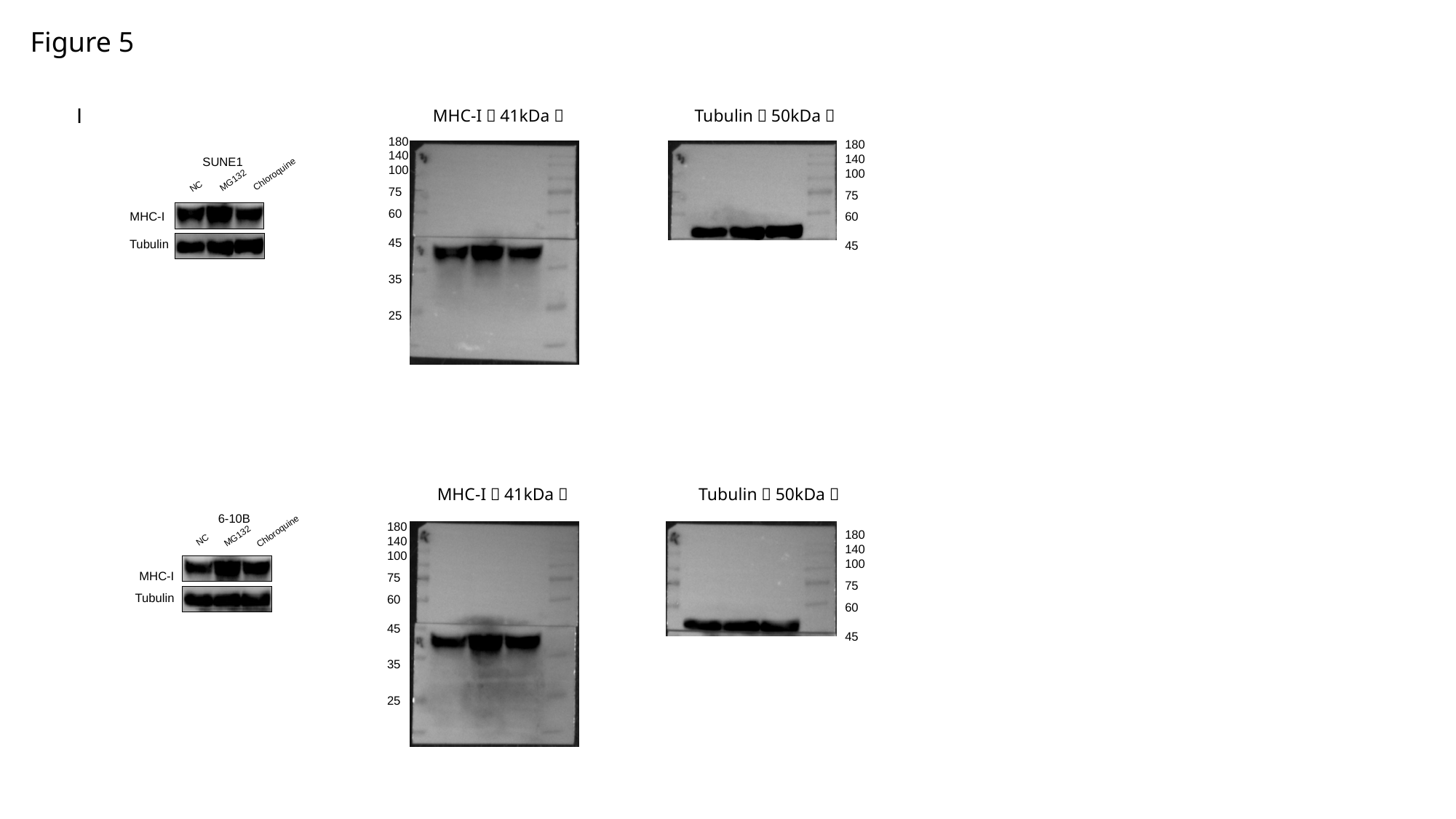

Figure 5
I
MHC-I（41kDa） Tubulin（50kDa）
180
140
100
75
60
45
35
25
180
140
100
75
60
45
SUNE1
MHC-I
Tubulin
Chloroquine
MG132
NC
MHC-I（41kDa） Tubulin（50kDa）
6-10B
MHC-I
Tubulin
Chloroquine
MG132
NC
180
140
100
75
60
45
35
25
180
140
100
75
60
45

## Slide 16
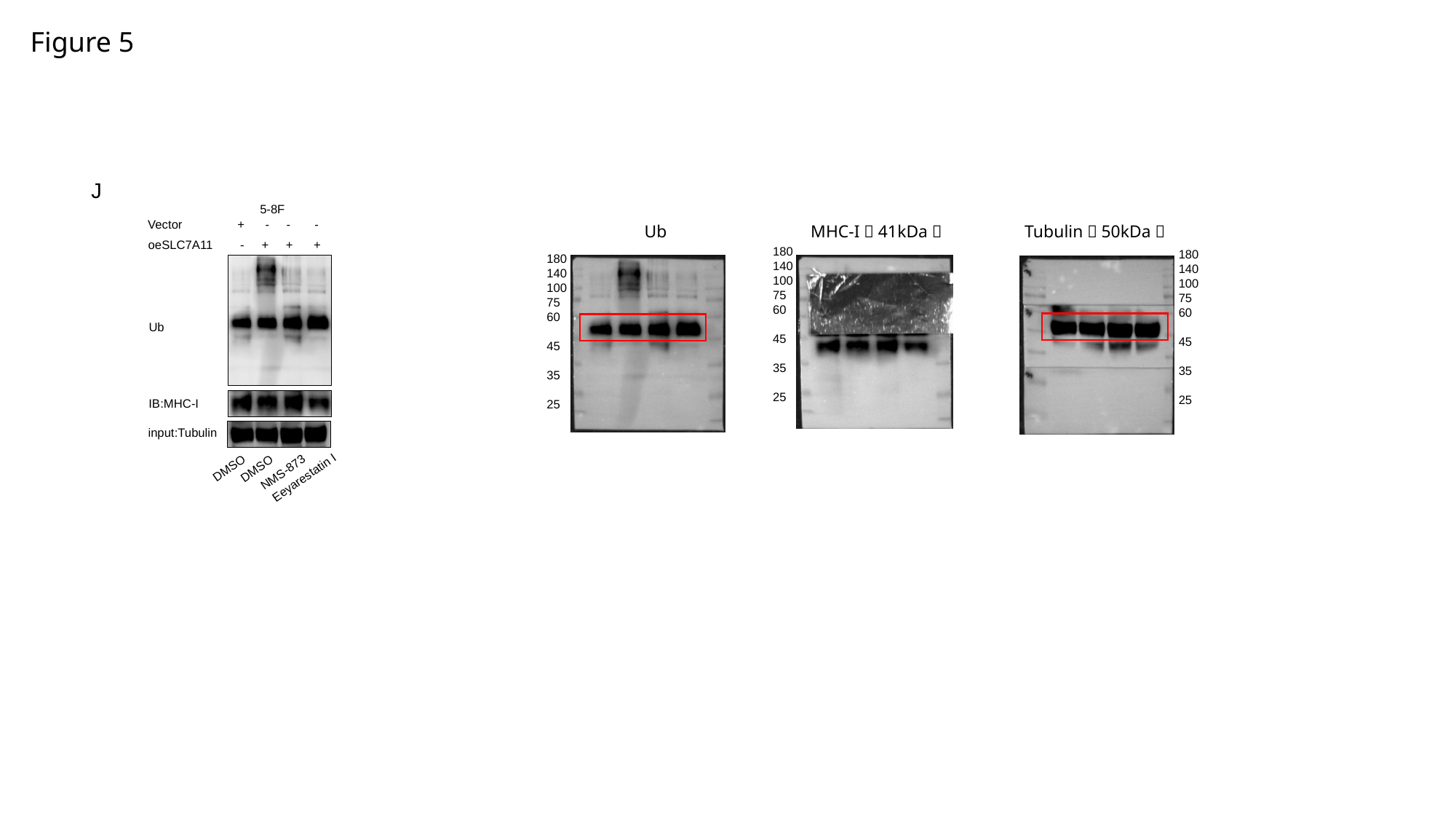

Figure 5
J
5-8F
Ub
DMSO
DMSO
oeSLC7A11 - + + +
NMS-873
Vector + - - -
Eeyarestatin I
input:Tubulin
IB:MHC-I
Ub MHC-I（41kDa） Tubulin（50kDa）
180
140
100
75
60
45
35
25
180
140
100
75
60
45
35
25
180
140
100
75
60
45
35
25

## Slide 17
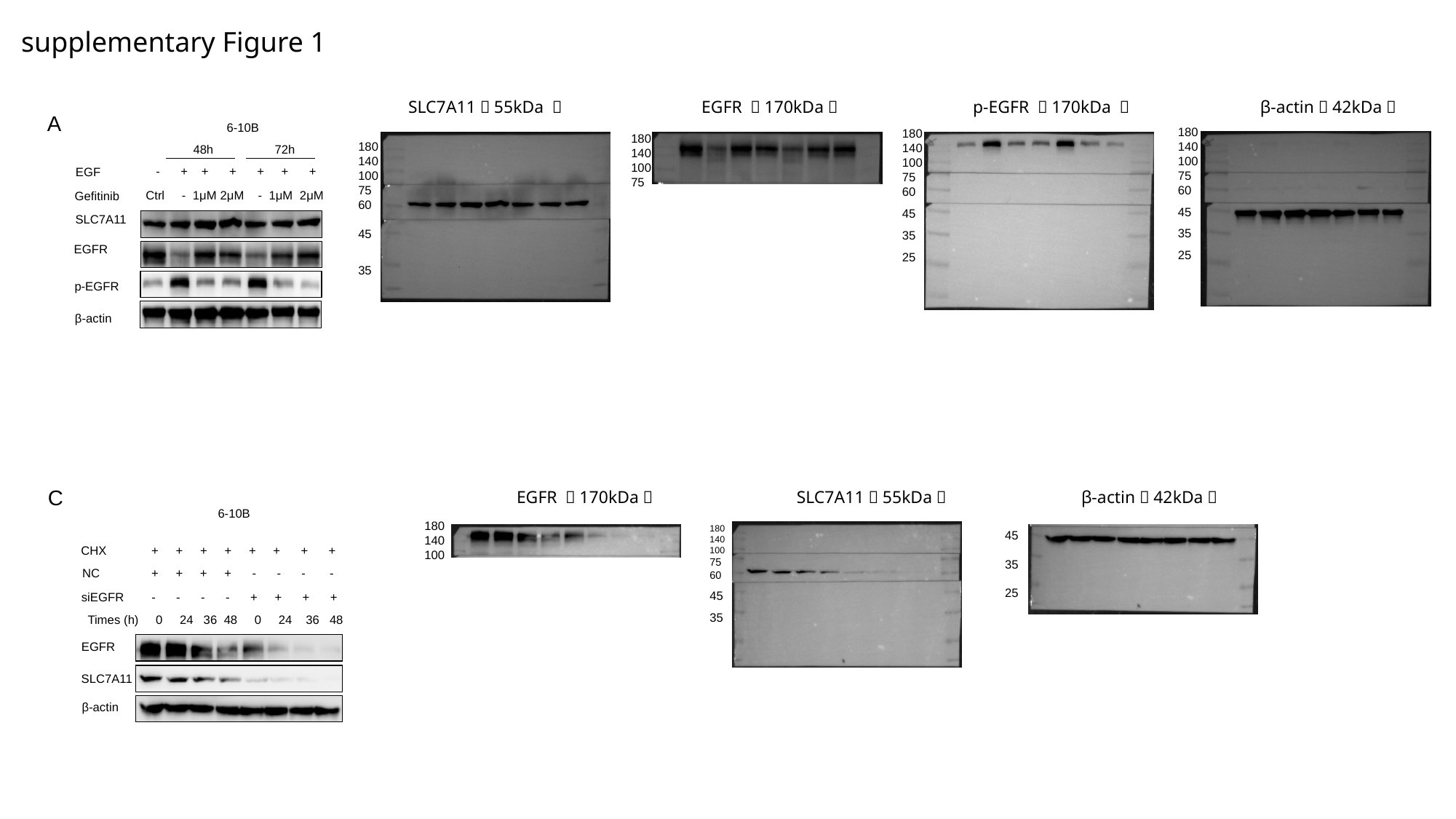

supplementary Figure 1
SLC7A11（55kDa ） EGFR （170kDa） p-EGFR （170kDa ） β-actin（42kDa）
A
6-10B
48h
72h
EGF
Gefitinib
SLC7A11
EGFR
p-EGFR
β-actin
 - + + + + + +
Ctrl - 1μM 2μM - 1μM 2μM
180
140
100
75
60
45
35
25
180
140
100
75
60
45
35
25
180
140
100
75
180
140
100
75
60
45
35
C
EGFR （170kDa） SLC7A11（55kDa） β-actin（42kDa）
6-10B
EGFR
SLC7A11
β-actin
CHX + + + + + + + +
NC + + + + - - - -
siEGFR - - - - + + + +
Times (h) 0 24 36 48 0 24 36 48
180
140
100
45
35
25
180
140
100
75
60
45
35

## Slide 18
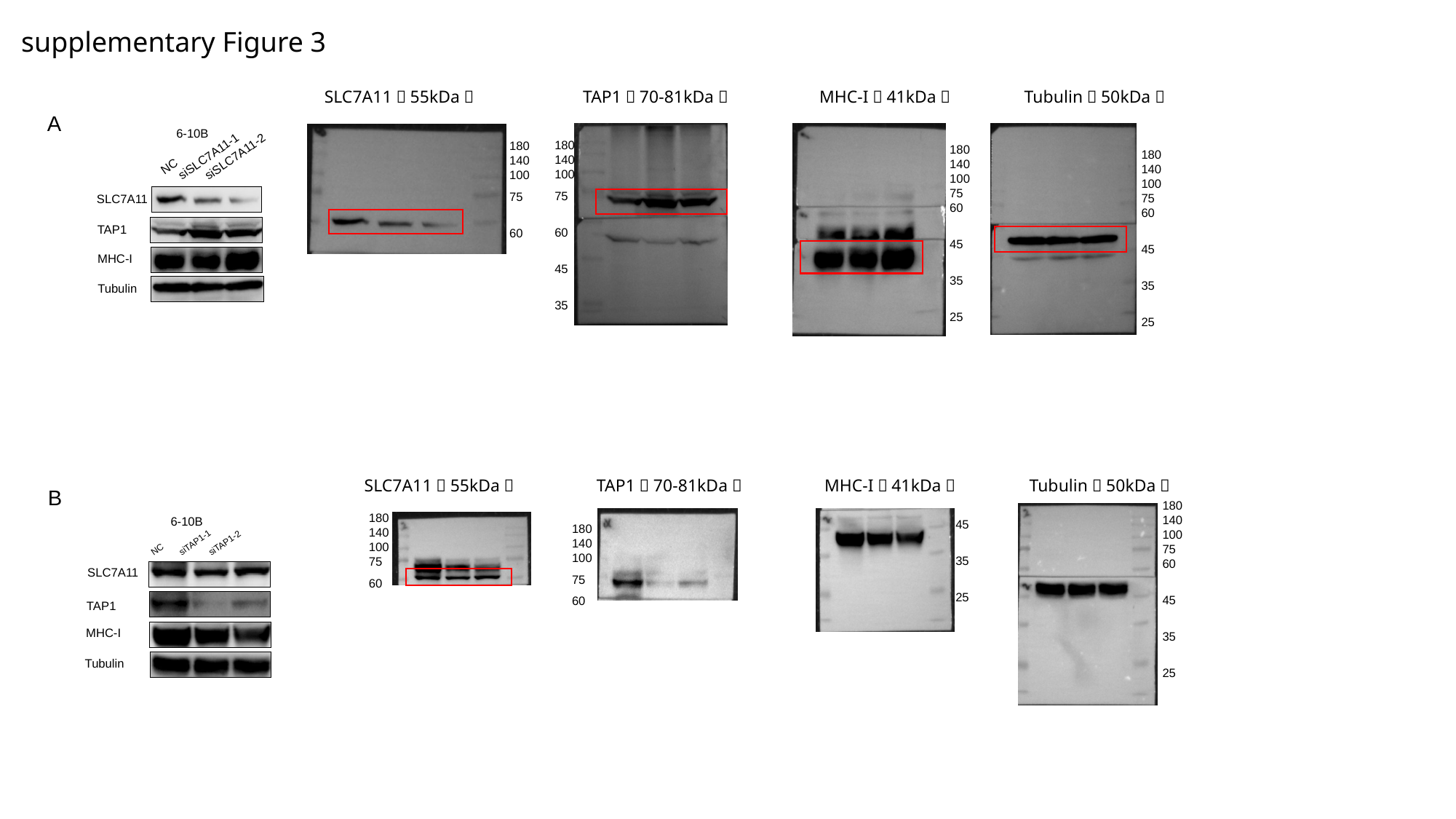

supplementary Figure 3
SLC7A11（55kDa） TAP1（70-81kDa） MHC-I（41kDa） Tubulin（50kDa）
A
6-10B
siSLC7A11-1
siSLC7A11-2
NC
SLC7A11
TAP1
MHC-I
Tubulin
180
140
100
75
60
180
140
100
75
60
45
35
180
140
100
75
60
45
35
25
180
140
100
75
60
45
35
25
SLC7A11（55kDa） TAP1（70-81kDa） MHC-I（41kDa） Tubulin（50kDa）
B
45
35
25
180
140
100
75
60
45
35
25
180
140
100
75
60
6-10B
siTAP1-1
siTAP1-2
NC
TAP1
MHC-I
Tubulin
SLC7A11
180
140
100
75
60

## Slide 19
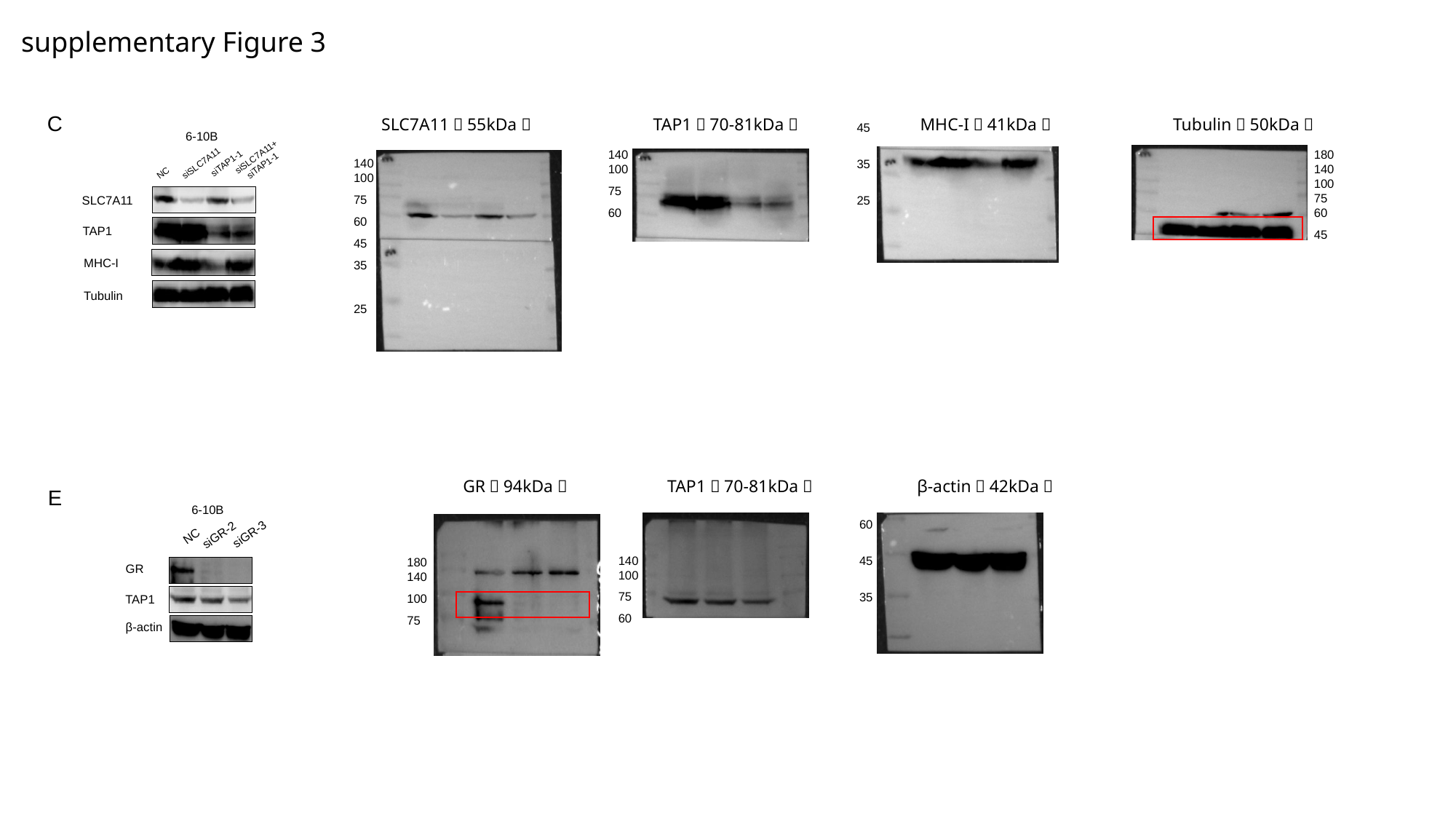

supplementary Figure 3
45
35
25
C
SLC7A11（55kDa） TAP1（70-81kDa） MHC-I（41kDa） Tubulin（50kDa）
6-10B
siSLC7A11+
siTAP1-1
siTAP1-1
siSLC7A11
NC
TAP1
MHC-I
Tubulin
SLC7A11
140
100
75
60
180
140
100
75
60
45
140
100
75
60
45
35
25
GR（94kDa） TAP1（70-81kDa） β-actin（42kDa）
E
60
45
35
6-10B
siGR-3
siGR-2
NC
GR
TAP1
β-actin
140
100
75
60
180
140
100
75

## Slide 20
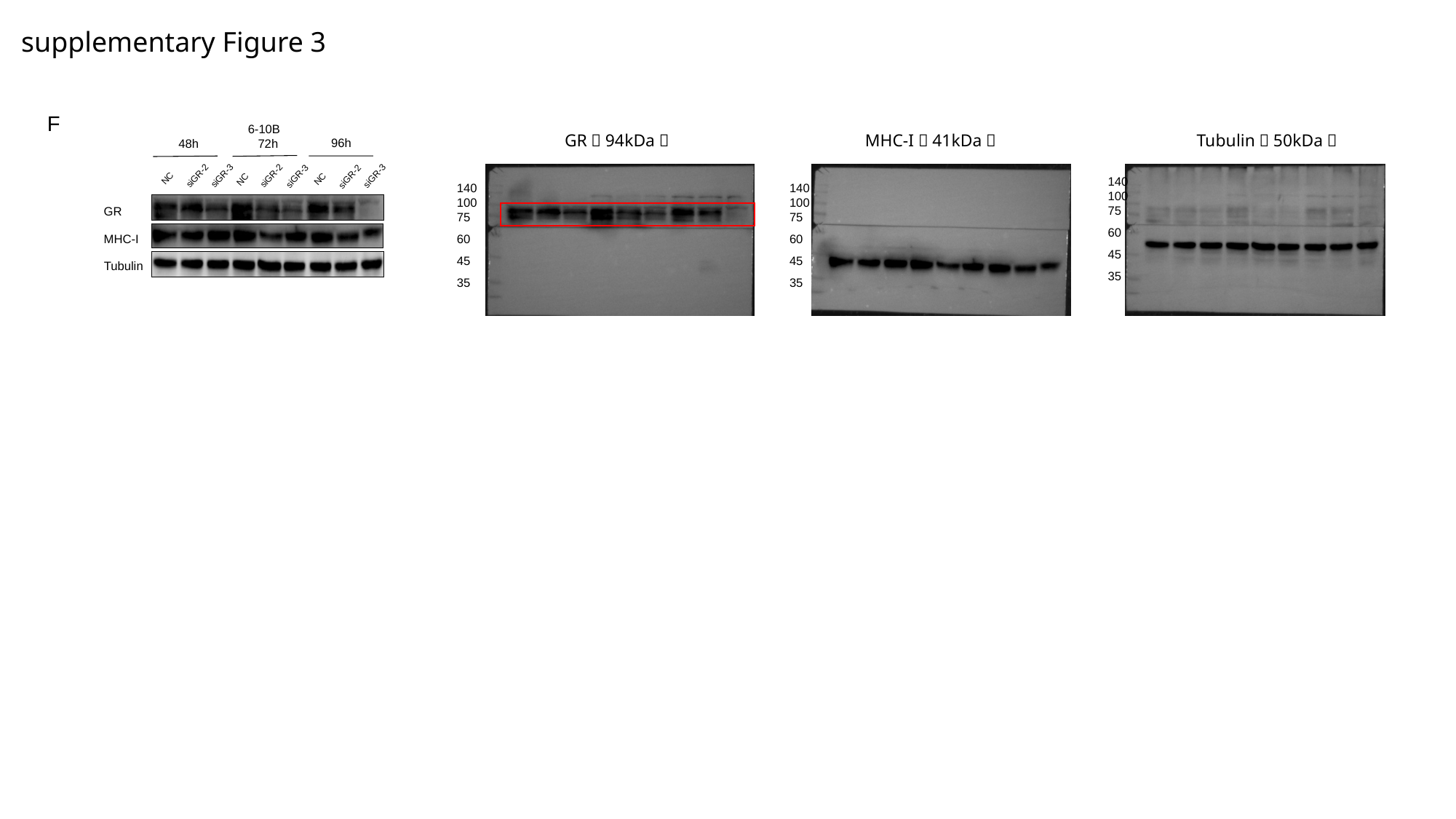

supplementary Figure 3
F
6-10B
96h
48h
72h
siGR-3
siGR-2
siGR-2
siGR-3
siGR-3
siGR-2
NC
NC
NC
GR
MHC-I
Tubulin
GR（94kDa） MHC-I（41kDa） Tubulin（50kDa）
140
100
75
60
45
35
140
100
75
60
45
35
140
100
75
60
45
35

## Slide 21
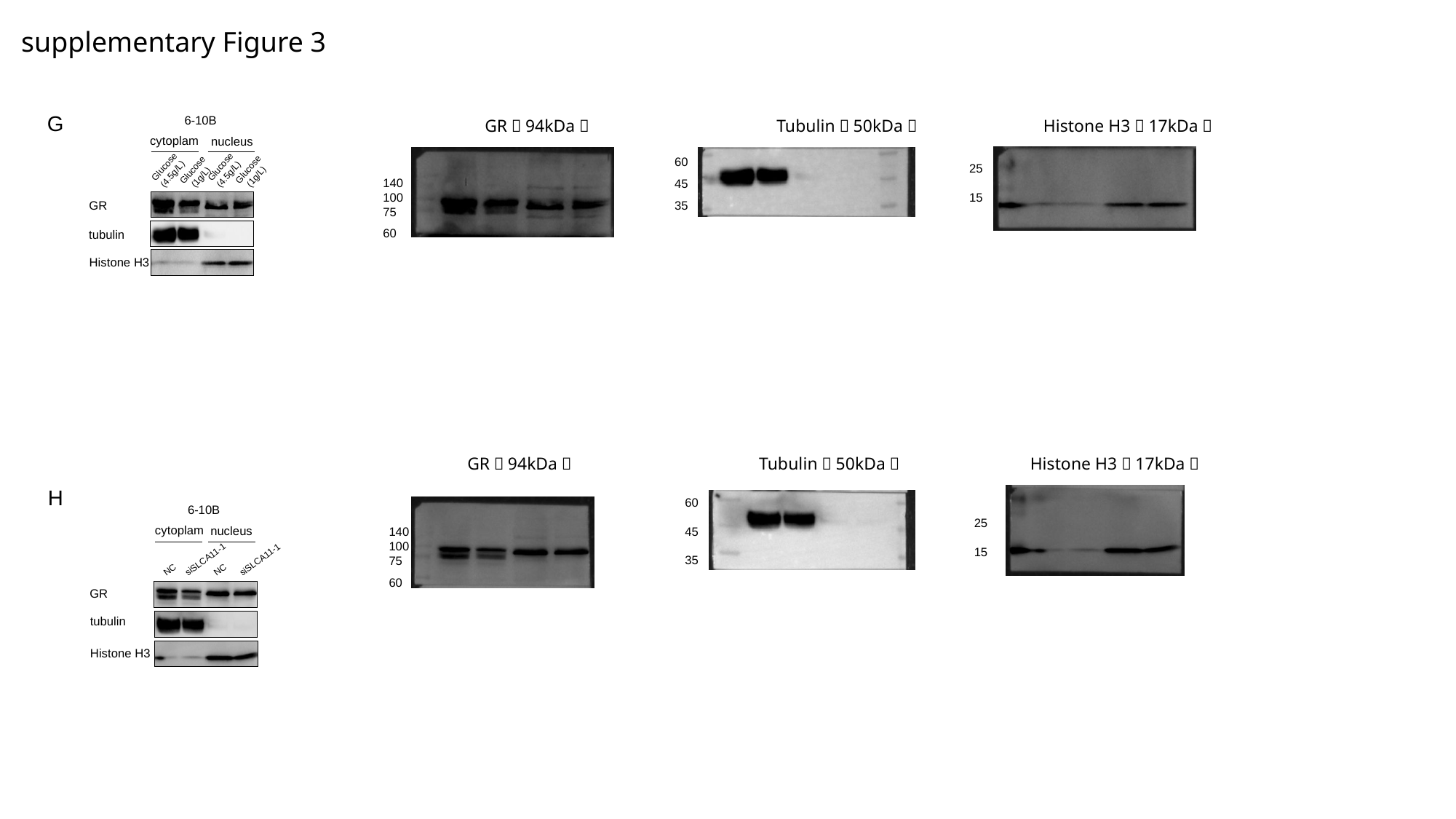

supplementary Figure 3
G
6-10B
cytoplam
nucleus
Glucose
(4.5g/L)
Glucose
(4.5g/L)
Glucose
(1g/L)
Glucose
(1g/L)
GR
tubulin
Histone H3
GR（94kDa） Tubulin（50kDa） Histone H3（17kDa）
60
45
35
25
15
140
100
75
60
GR（94kDa） Tubulin（50kDa） Histone H3（17kDa）
60
45
35
H
6-10B
cytoplam
nucleus
siSLCA11-1
siSLCA11-1
NC
NC
GR
tubulin
Histone H3
25
15
140
100
75
60

## Slide 22
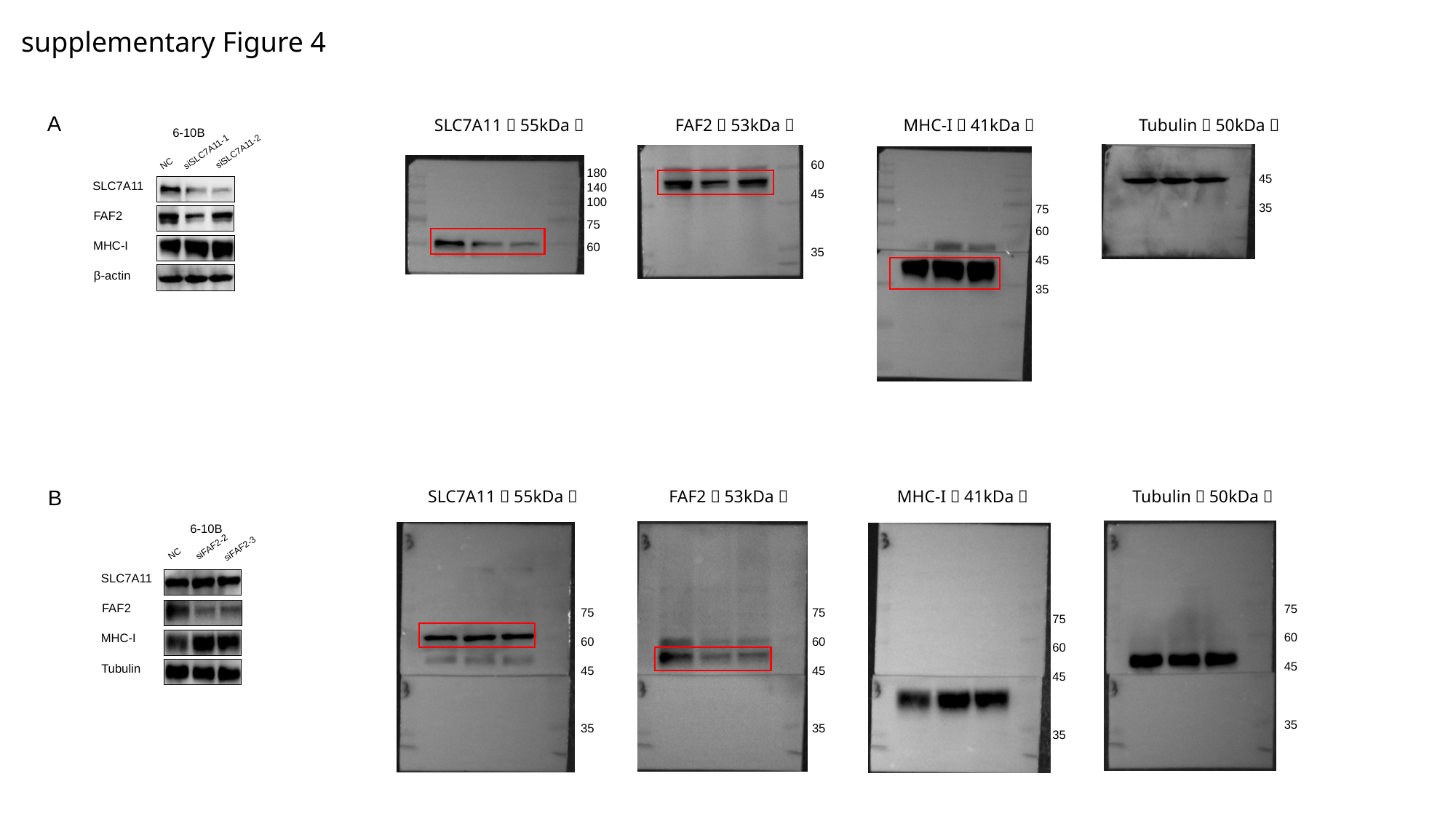

supplementary Figure 4
A
SLC7A11（55kDa） FAF2（53kDa） MHC-I（41kDa） Tubulin（50kDa）
6-10B
siSLC7A11-2
siSLC7A11-1
NC
SLC7A11
FAF2
β-actin
MHC-I
60
45
35
45
35
180
140
100
75
60
75
60
45
35
B
SLC7A11（55kDa） FAF2（53kDa） MHC-I（41kDa） Tubulin（50kDa）
6-10B
FAF2
MHC-I
Tubulin
siFAF2-2
siFAF2-3
NC
SLC7A11
75
60
45
35
75
60
45
35
75
60
45
35
75
60
45
35

## Slide 23
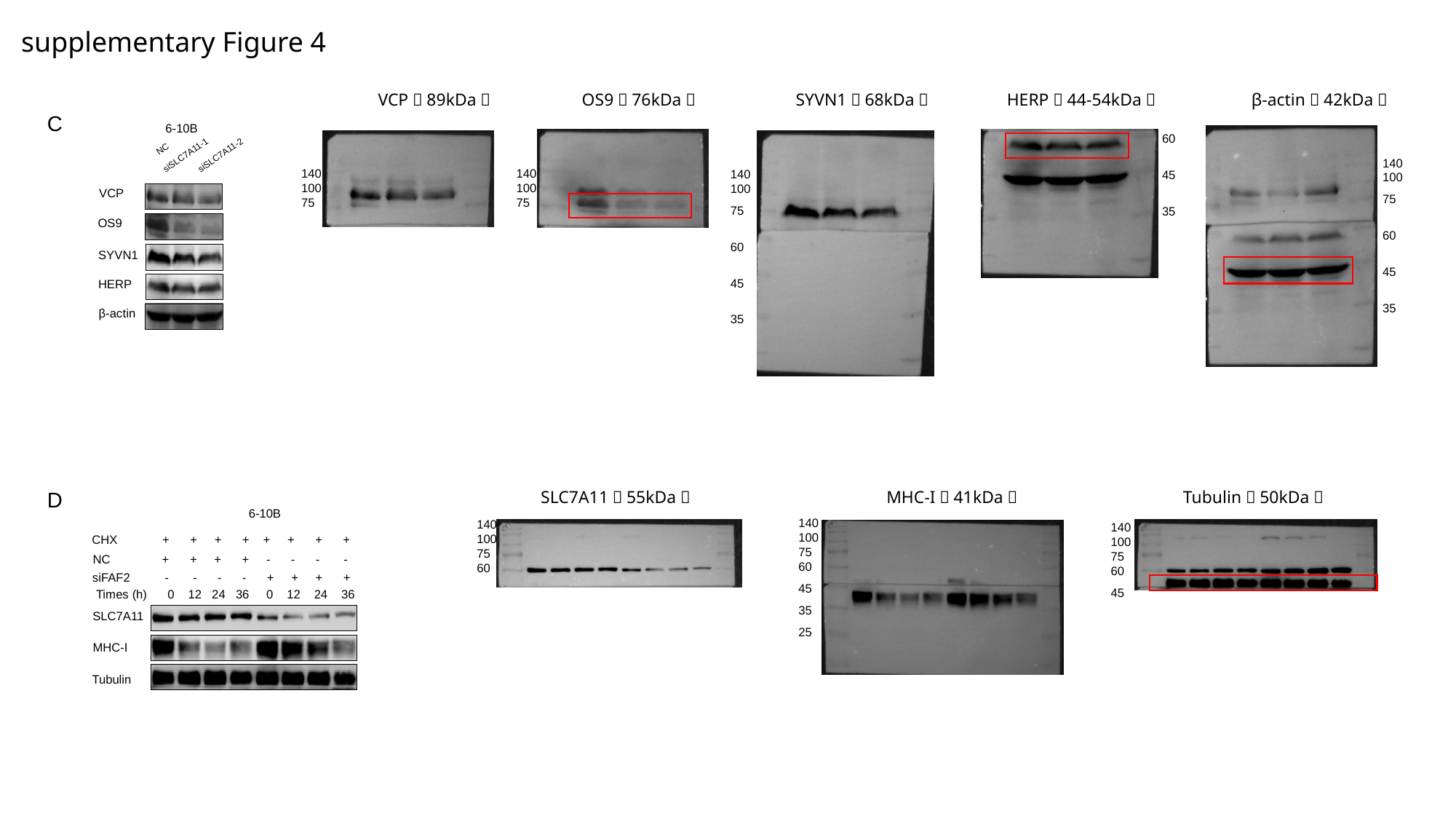

supplementary Figure 4
VCP（89kDa） OS9（76kDa） SYVN1（68kDa） HERP（44-54kDa） β-actin（42kDa）
60
45
35
C
6-10B
NC
siSLC7A11-2
siSLC7A11-1
VCP
OS9
SYVN1
HERP
β-actin
140
100
75
60
45
35
140
100
75
140
100
75
140
100
75
60
45
35
D
SLC7A11（55kDa） MHC-I（41kDa） Tubulin（50kDa）
6-10B
siFAF2 - - - - + + + +
SLC7A11
MHC-I
Tubulin
CHX + + + + + + + +
 NC + + + + - - - -
Times (h) 0 12 24 36 0 12 24 36
140
100
75
60
45
35
25
140
100
75
60
140
100
75
60
45
