# Supplementary material for: Targeting EGFR-binding protein SLC7A11 enhancing antitumor immunity of T cells via inducing MHC-I antigen presentation in nasopharyngeal carcinoma
Source: Cell Death Dis. 2025 Jan 16;16(1):21. doi: 10.1038/s41419-024-07327-9 (PMC11739652; doi:10.1038/s41419-024-07327-9)
Supplement: Supplementary file 2 — EGFR stabilizes SLC7A11 protein expression in NPC via a kinase-independent mechanism [file 41419_2024_7327_MOESM2_ESM.pptx]

## Slide 1
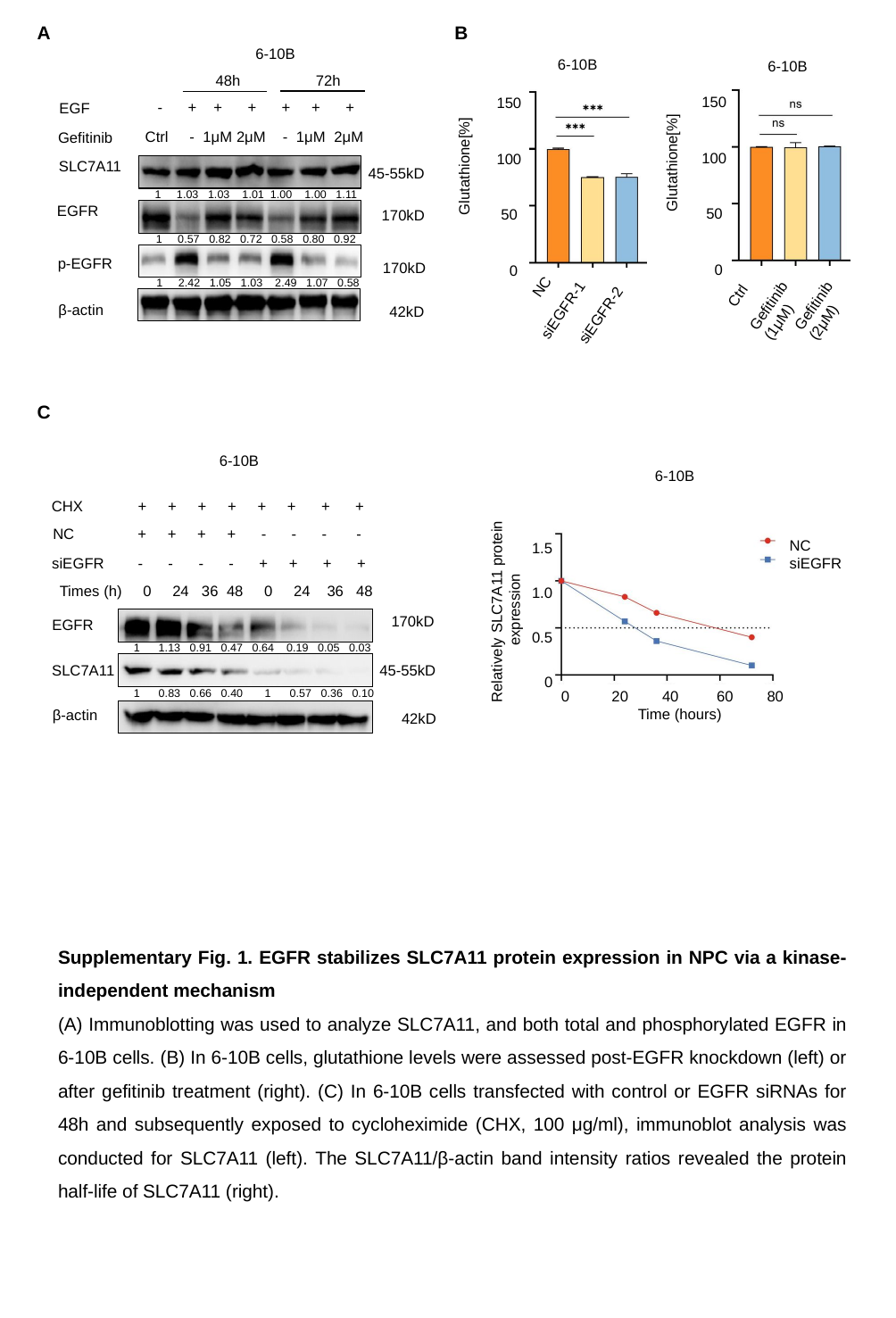

A B
C
6-10B
48h
72h
EGF
Gefitinib
SLC7A11
EGFR
p-EGFR
β-actin
 - + + + + + +
Ctrl - 1μM 2μM - 1μM 2μM
45-55kD
170kD
170kD
42kD
 1 1.03 1.03 1.01 1.00 1.00 1.11
 1 0.57 0.82 0.72 0.58 0.80 0.92
 1 2.42 1.05 1.03 2.49 1.07 0.58
150
100
 50
 0
6-10B
Glutathione[%]
Ctrl
Gefitinib
(2μM)
Gefitinib
(1μM)
6-10B
Glutathione[%]
NC
siEGFR-1
siEGFR-2
150
100
 50
 0
6-10B
EGFR
SLC7A11
β-actin
CHX + + + + + + + +
NC + + + + - - - -
siEGFR - - - - + + + +
Times (h) 0 24 36 48 0 24 36 48
170kD
45-55kD
42kD
 1 1.13 0.91 0.47 0.64 0.19 0.05 0.03
 1 0.83 0.66 0.40 1 0.57 0.36 0.10
6-10B
1.5
1.0
0.5
 0
NC
siEGFR
Relatively SLC7A11 protein
 expression
0 20 40 60 80
Time (hours)
Supplementary Fig. 1. EGFR stabilizes SLC7A11 protein expression in NPC via a kinase-independent mechanism
(A) Immunoblotting was used to analyze SLC7A11, and both total and phosphorylated EGFR in 6-10B cells. (B) In 6-10B cells, glutathione levels were assessed post-EGFR knockdown (left) or after gefitinib treatment (right). (C) In 6-10B cells transfected with control or EGFR siRNAs for 48h and subsequently exposed to cycloheximide (CHX, 100 μg/ml), immunoblot analysis was conducted for SLC7A11 (left). The SLC7A11/β-actin band intensity ratios revealed the protein half-life of SLC7A11 (right).
