# Supplementary material for: Targeting EGFR-binding protein SLC7A11 enhancing antitumor immunity of T cells via inducing MHC-I antigen presentation in nasopharyngeal carcinoma
Source: Cell Death Dis. 2025 Jan 16;16(1):21. doi: 10.1038/s41419-024-07327-9 (PMC11739652; doi:10.1038/s41419-024-07327-9)
Supplement: Supplementary file 3 — Histogram of predicted immune cell infiltrate levels in the GSE53819 dataset which with higher SLC7A11 levels in NPC tissues [file 41419_2024_7327_MOESM3_ESM.pptx]

## Slide 1
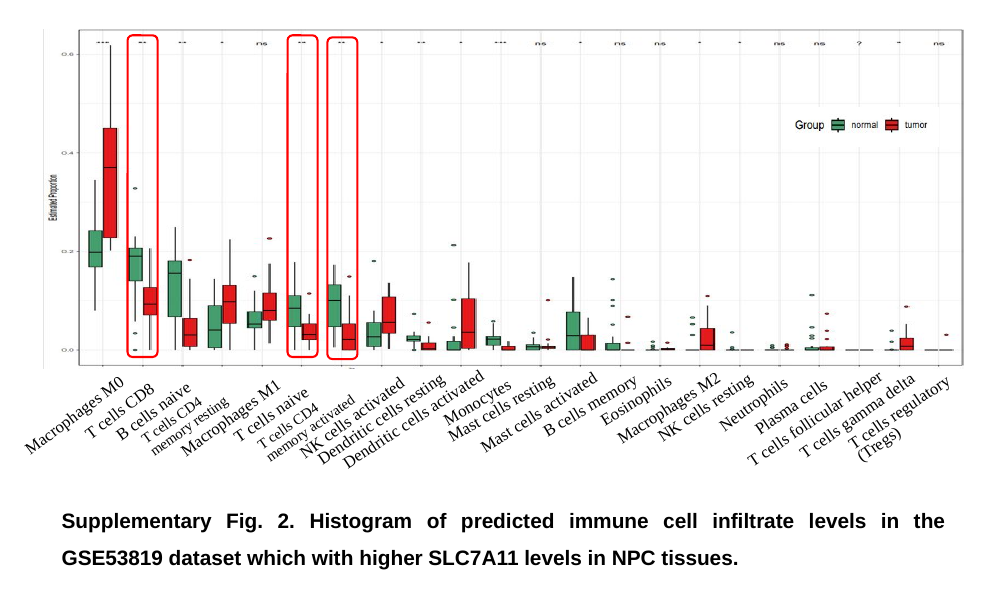

Eosinophils
Monocytes
Neutrophils
B cells memory
Plasma cells
NK cells resting
Mast cells resting
Macrophages M2
T cells CD8
B cells naive
Mast cells activated
T cells naive
T cells regulatory
(Tregs)
Macrophages M0
T cells gamma delta
T cells CD4
memory resting
NK cells activated
Macrophages M1
T cells follicular helper
Dendritic cells resting
T cells CD4
memory activated
Dendritic cells activated
Supplementary Fig. 2. Histogram of predicted immune cell infiltrate levels in the GSE53819 dataset which with higher SLC7A11 levels in NPC tissues.
