# Supplementary material for: Targeting EGFR-binding protein SLC7A11 enhancing antitumor immunity of T cells via inducing MHC-I antigen presentation in nasopharyngeal carcinoma
Source: Cell Death Dis. 2025 Jan 16;16(1):21. doi: 10.1038/s41419-024-07327-9 (PMC11739652; doi:10.1038/s41419-024-07327-9)
Supplement: Supplementary file 4 — Glucose-dependent properties of high SLC7A11 expression inhibit TAP1 transcription through GR, weakening MHC-I membrane expression in NPC cells [file 41419_2024_7327_MOESM4_ESM.pptx]

## Slide 1
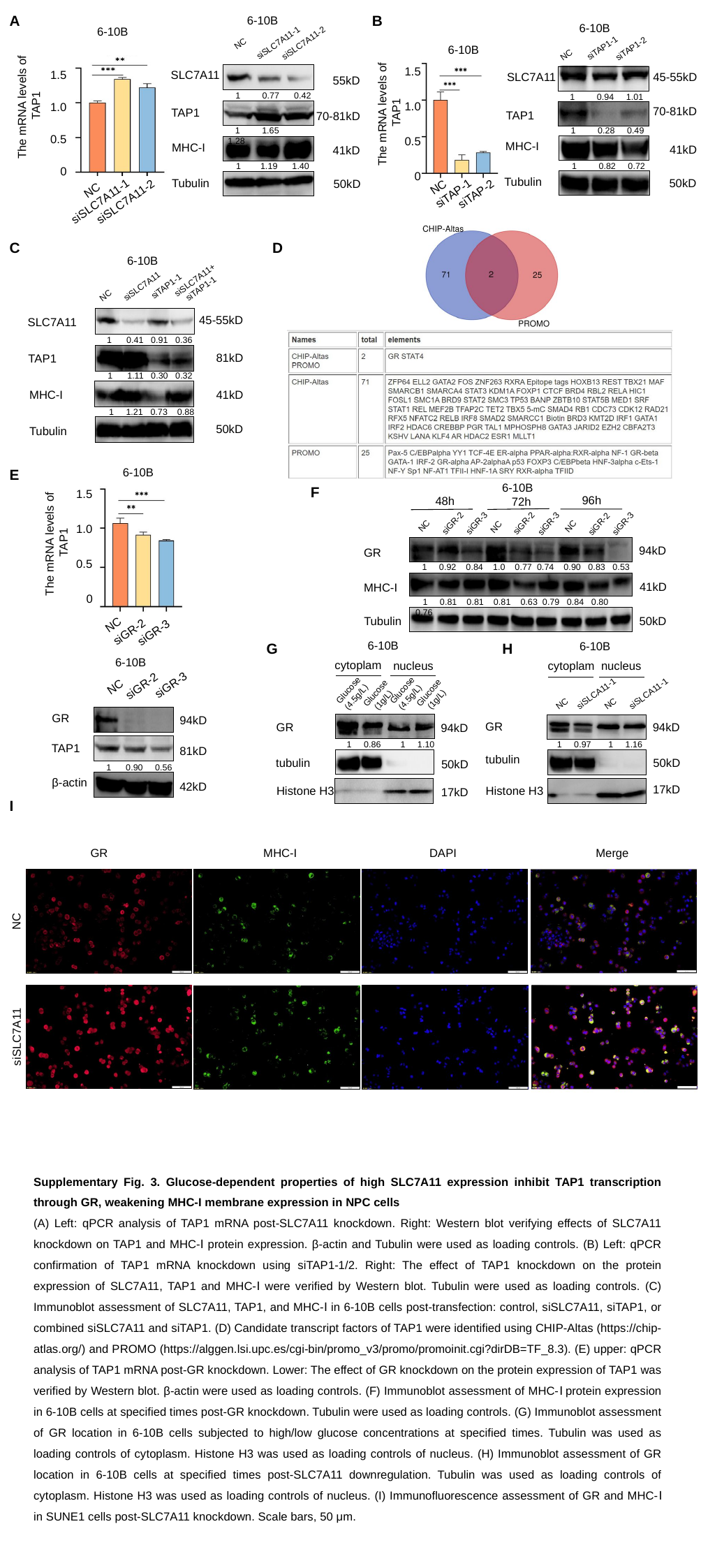

A B
C D
E
 F
 G H
I
6-10B
cytoplam
nucleus
Glucose
(4.5g/L)
Glucose
(4.5g/L)
Glucose
(1g/L)
Glucose
(1g/L)
GR
tubulin
Histone H3
94kD
50kD
17kD
 1 0.86 1 1.10
6-10B
siSLC7A11-1
siSLC7A11-2
NC
SLC7A11
TAP1
MHC-I
Tubulin
 55kD
70-81kD
41kD
50kD
 1 0.77 0.42
 1 1.65 1.28
 1 1.19 1.40
6-10B
siTAP1-1
siTAP1-2
NC
TAP1
MHC-I
Tubulin
SLC7A11
45-55kD
70-81kD
41kD
50kD
 1 0.94 1.01
 1 0.28 0.49
 1 0.82 0.72
6-10B
1.5
1.0
0.5
 0
The mRNA levels of
TAP1
NC
siSLC7A11-1
siSLC7A11-2
6-10B
1.5
1.0
0.5
 0
The mRNA levels of
TAP1
NC
siTAP-1
siTAP-2
6-10B
siSLC7A11+
siTAP1-1
siTAP1-1
siSLC7A11
NC
TAP1
MHC-I
Tubulin
SLC7A11
45-55kD
81kD
41kD
50kD
 1 0.41 0.91 0.36
 1 1.11 0.30 0.32
 1 1.21 0.73 0.88
6-10B
1.5
1.0
0.5
 0
The mRNA levels of
TAP1
NC
siGR-2
siGR-3
6-10B
96h
48h
72h
siGR-3
siGR-2
siGR-2
siGR-3
siGR-3
siGR-2
NC
NC
NC
GR
MHC-I
Tubulin
94kD
41kD
50kD
 1 0.92 0.84 1.0 0.77 0.74 0.90 0.83 0.53
 1 0.81 0.81 0.81 0.63 0.79 0.84 0.80 0.76
6-10B
cytoplam
nucleus
siSLCA11-1
siSLCA11-1
NC
NC
GR
tubulin
Histone H3
94kD
50kD
17kD
 1 0.97 1 1.16
6-10B
siGR-3
siGR-2
NC
GR
TAP1
β-actin
94kD
81kD
42kD
 1 0.90 0.56
GR MHC-I DAPI Merge
NC
siSLC7A11
Supplementary Fig. 3. Glucose-dependent properties of high SLC7A11 expression inhibit TAP1 transcription through GR, weakening MHC-I membrane expression in NPC cells
(A) Left: qPCR analysis of TAP1 mRNA post-SLC7A11 knockdown. Right: Western blot verifying effects of SLC7A11 knockdown on TAP1 and MHC-Ⅰ protein expression. β-actin and Tubulin were used as loading controls. (B) Left: qPCR confirmation of TAP1 mRNA knockdown using siTAP1-1/2. Right: The effect of TAP1 knockdown on the protein expression of SLC7A11, TAP1 and MHC-Ⅰ were verified by Western blot. Tubulin were used as loading controls. (C) Immunoblot assessment of SLC7A11, TAP1, and MHC-Ⅰ in 6-10B cells post-transfection: control, siSLC7A11, siTAP1, or combined siSLC7A11 and siTAP1. (D) Candidate transcript factors of TAP1 were identified using CHIP-Altas (https://chip-atlas.org/) and PROMO (https://alggen.lsi.upc.es/cgi-bin/promo_v3/promo/promoinit.cgi?dirDB=TF_8.3). (E) upper: qPCR analysis of TAP1 mRNA post-GR knockdown. Lower: The effect of GR knockdown on the protein expression of TAP1 was verified by Western blot. β-actin were used as loading controls. (F) Immunoblot assessment of MHC-Ⅰ protein expression in 6-10B cells at specified times post-GR knockdown. Tubulin were used as loading controls. (G) Immunoblot assessment of GR location in 6-10B cells subjected to high/low glucose concentrations at specified times. Tubulin was used as loading controls of cytoplasm. Histone H3 was used as loading controls of nucleus. (H) Immunoblot assessment of GR location in 6-10B cells at specified times post-SLC7A11 downregulation. Tubulin was used as loading controls of cytoplasm. Histone H3 was used as loading controls of nucleus. (I) Immunofluorescence assessment of GR and MHC-Ⅰ in SUNE1 cells post-SLC7A11 knockdown. Scale bars, 50 μm.
