# Supplementary material for: Targeting EGFR-binding protein SLC7A11 enhancing antitumor immunity of T cells via inducing MHC-I antigen presentation in nasopharyngeal carcinoma
Source: Cell Death Dis. 2025 Jan 16;16(1):21. doi: 10.1038/s41419-024-07327-9 (PMC11739652; doi:10.1038/s41419-024-07327-9)
Supplement: Supplementary file 5 — SLC7A11 enhances FAF2 expression, activating the ERAD pathway implicated in the ubiquitin-mediated degradation of MHC-I [file 41419_2024_7327_MOESM5_ESM.pptx]

## Slide 1
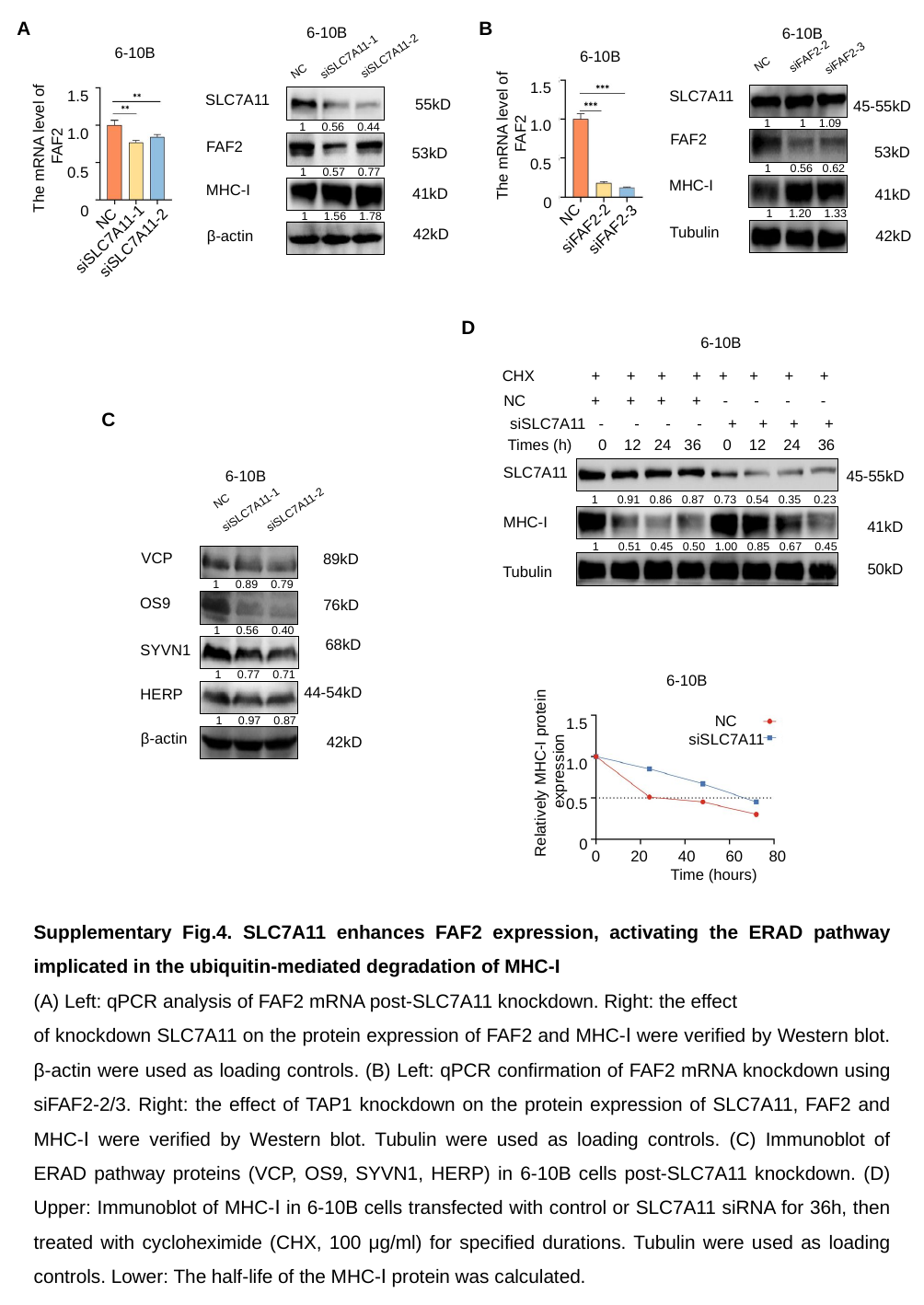

A B
 D
 C
6-10B
siSLC7A11-2
siSLC7A11-1
NC
SLC7A11
FAF2
β-actin
MHC-I
 55kD
53kD
41kD
42kD
 1 0.56 0.44
 1 0.57 0.77
 1 1.56 1.78
6-10B
FAF2
MHC-I
Tubulin
siFAF2-2
siFAF2-3
NC
SLC7A11
45-55kD
53kD
41kD
42kD
 1 1 1.09
 1 0.56 0.62
 1 1.20 1.33
6-10B
1.5
1.0
0.5
 0
The mRNA level of
 FAF2
NC
siSLC7A11-1
siSLC7A11-2
6-10B
1.5
1.0
0.5
 0
NC
siFAF2-2
siFAF2-3
The mRNA level of
 FAF2
6-10B
siSLC7A11 - - - - + + + +
SLC7A11
MHC-I
Tubulin
CHX + + + + + + + +
 NC + + + + - - - -
Times (h) 0 12 24 36 0 12 24 36
45-55kD
41kD
50kD
 1 0.91 0.86 0.87 0.73 0.54 0.35 0.23
 1 0.51 0.45 0.50 1.00 0.85 0.67 0.45
6-10B
NC
siSLC7A11-2
siSLC7A11-1
VCP
OS9
SYVN1
HERP
β-actin
89kD
76kD
68kD
44-54kD
42kD
 1 0.89 0.79
 1 0.56 0.40
 1 0.77 0.71
 1 0.97 0.87
6-10B
1.5
1.0
0.5
 0
 NC
 siSLC7A11
Relatively MHC-I protein
 expression
0 20 40 60 80
Time (hours)
Supplementary Fig.4. SLC7A11 enhances FAF2 expression, activating the ERAD pathway implicated in the ubiquitin-mediated degradation of MHC-I
(A) Left: qPCR analysis of FAF2 mRNA post-SLC7A11 knockdown. Right: the effect
of knockdown SLC7A11 on the protein expression of FAF2 and MHC-Ⅰ were verified by Western blot. β-actin were used as loading controls. (B) Left: qPCR confirmation of FAF2 mRNA knockdown using siFAF2-2/3. Right: the effect of TAP1 knockdown on the protein expression of SLC7A11, FAF2 and MHC-Ⅰ were verified by Western blot. Tubulin were used as loading controls. (C) Immunoblot of ERAD pathway proteins (VCP, OS9, SYVN1, HERP) in 6-10B cells post-SLC7A11 knockdown. (D) Upper: Immunoblot of MHC-Ⅰ in 6-10B cells transfected with control or SLC7A11 siRNA for 36h, then treated with cycloheximide (CHX, 100 μg/ml) for specified durations. Tubulin were used as loading controls. Lower: The half-life of the MHC-Ⅰ protein was calculated.
